# Altered Reward Processing in Obsessive–Compulsive Disorder: Insights From Active and Observational Learning

**DOI:** 10.1111/psyp.70142

**Published:** 2025-09-07

**Authors:** Julian Vahedi, Armin Bahic, Irini Chaliani, Leonhard Schilbach, Burkhard Ciupka‐Schön, Christian Bellebaum, Reinhard Pietrowsky, Jutta Peterburs

**Affiliations:** ^1^ Faculty of Mathematics and Natural Sciences Heinrich Heine University Düsseldorf Düsseldorf Germany; ^2^ Clinic for Psychiatry and Psychotherapy/LVR‐Clinic Düsseldorf, Medical Faculty and University Hospital Düsseldorf Heinrich Heine University Düsseldorf Düsseldorf Germany; ^3^ Medical Faculty Ludwig‐Maximilians‐Universität München München Germany; ^4^ Department of Human Medicine, Institute for Systems Medicine MSH Medical School Hamburg Hamburg Germany

**Keywords:** agency, FRN/RewP, learning, obsessive–compulsive disorder, performance monitoring, prediction error

## Abstract

Obsessive–compulsive disorder (OCD) has been associated with altered performance monitoring, reflected in enhanced amplitudes of the error‐related negativity in the event‐related potential. However, this is not specific to OCD, as overactive error processing is also evident in anxiety. Although similar neural mechanisms have been proposed for error and feedback processing, it remains unclear whether the processing of errors as indexed by external feedback, reflected in the feedback‐related negativity (FRN), is altered in OCD. Likewise, it is currently unknown whether performance monitoring in OCD differs between learning from self‐performed and observed outcomes. The present study compared OCD patients (*n* = 27) with healthy controls (HCs; *n* = 27) and patients with social anxiety disorder (SAD; *n* = 29) in an active and observational variant of a probabilistic feedback learning task while EEG was recorded. Compared to HCs, OCD patients showed generally impaired task performance across both active and observational learning, as well as more indecisive choice behavior. This was accompanied by generally more positive amplitudes of the FRN, with enhanced valence coding for active compared to observational learning, driven by more positive FRN amplitudes for wins. However, no differences were found for losses. Overall, these results suggest deficient reward—rather than punishment—processing in OCD. Similar performance monitoring alterations in OCD and SAD imply reliance on shared, disorder‐general mechanisms. Possible candidates for these mechanisms, such as intolerance of uncertainty, pessimism, and depressiveness are discussed.

## Abstract 2

This study is the first to reveal impairments in both active and observational learning in obsessive–compulsive disorder (OCD). This was accompanied by aberrant reward—rather than punishment—processing, reflected in more positive amplitudes of the feedback‐related negativity to positive outcomes, even after controlling for reward expectation. Comparing OCD patients to those with social anxiety disorder revealed largely similar alterations in performance monitoring, highlighting the involvement of broader, transdiagnostic mechanisms.

Obsessive–compulsive disorder (OCD) is a disabling mental disorder characterized by intrusive thoughts (*obsessions*) and/or repetitive behaviors (*compulsions*; American Psychiatric Association 2013). Neurobiological models suggest that dysregulated cortico‐striato‐thalamo‐cortical (CSTC) circuits play a pivotal role in the pathophysiology of OCD (Aouizerate et al. 2004; Milad and Rauch 2012; Saxena et al. 1998; Saxena and Rauch 2000), while these circuits also underlie performance monitoring and adaptive behavior (Ullsperger, Danielmeier, et al. 2014; Ullsperger and von Cramon 2006). Consistent with this, OCD patients often exhibit deficits in action monitoring and impulse control, with feelings of discomfort and incompleteness persisting until completing a compulsive or mental ritual (Coles et al. 2003, 2005; Wahl et al. 2008). Accordingly, obsessive–compulsive (OC) symptoms have been linked to persistent hyperactive error signals in the brain (Pitman 1987), suggesting impairments in the integration of action‐related feedback to flexibly guide behavior (Olley et al. 2007).

In support of this notion, neuroimaging studies have shown that activity within the posterior medial frontal cortex (pMFC), including the midcingulate cortex (MCC), is abnormally increased in OCD patients when committing an error (e.g., Fitzgerald et al. 2005; Grützmann et al. 2016; for an overview see Norman et al. 2019). An entire line of research has focused on error processing via the error‐related negativity (ERN), a component of the event‐related potential (ERP), characterized by a negative deflection peaking within 100 ms after error commission at fronto‐central electrode sites (Falkenstein et al. 1991; Gehring et al. 1993; for a review see Gehring et al. 2012). A similar, albeit markedly reduced, negative deflection is also seen for correct responses and is referred to as the correct‐related negativity (CRN).

Crucially, the MCC has been identified as the primary neural generator of the ERN (Debener et al. 2005; Dehaene et al. 1994; Fu et al. 2022). Not surprisingly, OCD has been associated with ERN enhancement (Endrass et al. 2008; Ruchsow et al. 2005; for a review see Riesel 2019). Although the ERN is not affected by OCD symptom severity (Nawani et al. 2018) or expression (Riesel et al. 2014), ERN hyperactivity in OCD appears context dependent. For instance, Riesel (2019) found ERN enhancement only during action monitoring under response conflict, but not during other tasks, including feedback learning.

While during error monitoring the correctness of specific behavior is known to the agent already during action execution based on *internal* information, feedback learning requires *external* feedback to signal whether current behavior is (un)favorable and should be (dis)continued. Feedback learning has been primarily linked to the mesencephalic dopamine (DA) system (Arias‐Carrión et al. 2010) and reward‐related frontostriatal circuits (Balleine et al. 2007; Cox and Witten 2019; Samejima et al. 2005). In ERP research, feedback processing has been most extensively studied through the feedback‐related negativity (FRN), a fronto‐central negative deflection peaking approximately 200–350 ms after feedback presentation that is typically more pronounced for negative compared to positive feedback (Gehring and Willoughby 2002; Miltner et al. 1997; Nieuwenhuis et al. 2004; Yeung and Sanfey 2004; but also see Faßbender et al. 2023). As for the ERN, source localization suggests that the FRN is primarily generated in the MCC (Hauser et al. 2014; Luu et al. 2003; Oerlemans et al. 2024), though the FRN has also been linked to striatal processing (Becker et al. 2014; Carlson et al. 2011; Foti et al. 2011).

An influential framework posits that both ERN and FRN reflect prediction error (PE) signals, mediated by phasic activity of mesencephalic DA neurons: Specifically, negative PE signals—marked by dips in DA firing for events worse than expected—have been assumed to enlarge the ERN/FRN (Holroyd and Coles 2002). The functional difference between the ERN and FRN has thus mainly been explained in terms of the ERN reflecting *predictive* error processing based on internal information, while the FRN may reflect *postdictive* error processing based on external information (Holroyd and Coles 2002; Maurer et al. 2021; Walsh and Anderson 2012). However, a purely dopaminergic mechanism in generating ERN/FRN amplitudes is controversial and has been repeatedly questioned, given the rapid changes in post‐synaptic potentials required (e.g., Jocham and Ullsperger 2009; Ullsperger, Fischer, et al. 2014).

No matter the neural mechanism, the FRN has been repeatedly shown to reflect reward expectancy (Bellebaum and Daum 2008; Holroyd et al. 2004; Pfabigan et al. 2011) and PEs (Burnside et al. 2019; Fischer and Ullsperger 2013; Hoy et al. 2021; Sambrook and Goslin 2015). Still, there is an ongoing debate about whether the difference waveform signal in the time window of the FRN—with more negative amplitudes for negative than positive feedback—is in fact evoked by an increased negativity to unfavorable events, thus reflecting external error processing, or rather by a reward‐related positivity (RewP) to favorable outcomes, thus reflecting reward processing (Holroyd et al. 2008; Proudfit 2015). Indeed, the FRN is not only modulated by negative (Huvermann et al. 2025; Ichikawa et al. 2010) but also positive PEs, indicating events better than expected (Berlijn et al. 2025; Kirsch et al. 2022; Weber and Bellebaum 2024), suggesting it to be involved more generally in feedback processing by reflecting both punishment and reward processing (but see Hoy et al. 2021). While the terms FRN and RewP are often used interchangeably, we use the term FRN throughout this work, in accordance with Faßbender et al. (2023). Notably, other studies reported that the FRN may not reflect a *signed* but rather an *unsigned* PE, i.e., general unexpectedness or salience (e.g., Hauser et al. 2014; Oliveira et al. 2007; Pfabigan et al. 2015; Sallet et al. 2013; Talmi et al. 2013).

While error monitoring and the ERN have been extensively studied in OCD patients (e.g., see Norman et al. 2019; Riesel 2019), relatively few studies have addressed feedback monitoring and the FRN in OCD. Endrass and Ullsperger (2014) suggested a functional dissociation between error and feedback monitoring in OCD, with hyperactive error processing but hypoactive processing of external feedback. However, previous work is rather inconsistent, with some studies reporting an attenuated FRN in OCD patients (Endrass et al. 2013) and subclinical OCD (Gründler et al. 2009; O'Toole et al. 2012; Simons 2010), while others found increased FRN amplitudes in OCD patients (Luo et al. 2020) and subclinical OCD (Zhu et al. 2014). Yet other studies found no differences in FRN amplitudes between OCD patients and healthy controls (Nieuwenhuis et al. 2005; Schüller et al. 2015). Therefore, whether monitoring of errors as indicated by external (negative) performance feedback, reflected in the FRN, is altered in OCD remains unclear. Moreover, to our best knowledge, no previous study has investigated the relationship between the FRN and coding of PEs in OCD. Previous neuroimaging studies in OCD patients suggested enhanced PE coding in the anterior cingulate cortex (ACC) and putamen (Hauser et al. 2017; Murray et al. 2019), whereas Suzuki et al. (2023) reported attenuated PE coding. Given the shared neural basis for error and feedback processing, as well as the close link between overactive error monitoring and OCD, investigating feedback monitoring in OCD constitutes an important step toward a deeper understanding of OCD psychopathology.

Aside from monitoring one's own behavior and consequences, observing others provides valuable information to the performance monitoring system, particularly in social contexts. OCD has been linked to impaired social cognitive functioning, including increased affective responsiveness and empathic distress during social observation (Jansen et al. 2020). Altered social cognition may contribute to how OCD patients perceive and process information related to themselves or others, for example, how they process feedback related to other people's actions. However, to our best knowledge, there are no studies yet that explicitly examine observational performance monitoring in OCD.

Interestingly, observing others' errors or action–outcomes elicits activation of brain areas resembling those involved in the processing of one's own, including the (p)MFC, and extending from the ACC to the MCC (de Bruijn et al. 2009; Hill et al. 2016; Mobbs et al. 2009; Newman‐Norlund et al. 2009; Shane et al. 2008; but also see Morelli et al. 2015). Observed outcomes also evoke FRN‐like (e.g., Bellebaum et al. 2010; Yu and Zhou 2006) deflections in the ERP with comparable topography and shared neural origin in the MCC (Koban et al. 2012). However, amplitudes of the observer FRN are typically reduced (Koban et al. 2012) and less distinctive for positive versus negative feedback (Bellebaum and Colosio 2014; Bellebaum et al. 2010). Importantly, PE coding in the FRN appears agency‐dependent, with Burnside et al. (2019) showing that the FRN reflected PEs only for own but not observed outcomes. These results generally corroborate findings by Kobza et al. (2012) which suggest a less prominent role of the striatum in observational relative to active learning. Although many mental disorders have been linked to altered performance monitoring, no previous clinical studies have investigated feedback processing in an observational learning context. However, results by de la Asuncion et al. (2015) on error observation indicate that performance monitoring systems dissociate depending on agency in schizophrenic patients. It is thus conceivable that similar dissociations may also occur during feedback processing in OCD.

Recent developments in precision medicine and psychiatry have led to a rethinking of rigid categorical symptom‐based diagnostic classification, moving toward an integrative, transdiagnostic‐dimensional model of psychiatric nosology (Insel et al. 2010; Morris and Cuthbert 2012). The research domain criteria (RDoC) framework therefore aims to identify fundamental neurocognitive features underlying multiple phenotypically heterogeneous disorders (Morris and Cuthbert 2012). In line with that, overactive error monitoring as indexed by enhanced ERN amplitudes is not exclusive to OCD but has also been observed in various anxiety disorders (e.g., Carrasco et al. 2013; Endrass et al. 2014; Riesel et al. 2017; Weinberg et al. 2010). As a consequence, a better understanding of OCD requires identifying both disorder‐general, i.e., transdiagnostic, and disorder‐specific mechanisms, emphasizing the need for comparing OCD with nosologically distinct disorders, most promisingly within the anxiety spectrum. Here, social anxiety disorder (SAD) seems to be a reasonable candidate. SAD is among the most frequent co‐occurring disorders in OCD (Assuncao et al. 2012; Lochner et al. 2014; Ruscio et al. 2010) while sharing several overlapping symptoms, including doubt (Carpita et al. 2020), intolerance of uncertainty (Boelen and Reijntjes 2009), perfectionism, and fear of negative evaluation (Rudy et al. 2014). Both OCD and SAD have been linked to overactive monitoring of self‐committed errors (Endrass et al. 2014). However, no previous studies have directly compared OCD and SAD regarding feedback monitoring and the FRN.

The present study thus aimed to examine performance monitoring during active and observational learning in OCD. Following recent calls for transdiagnostic research, we additionally sought to identify disorder‐general and/or disorder‐specific alterations in OCD in comparison to SAD. To this end, patients with OCD, SAD, and healthy individuals completed an active and an observational variant of a probabilistic learning task. While our primary focus was on feedback processing, reflected in the FRN, we also examined action monitoring in active learning, through the ERN/CRN given their assumed conceptual and mechanistic overlap. This aligns with the broader OCD literature, which has predominantly focused on the ERN/CRN. Beyond that, as alterations in feedback processing in OCD have often been interpreted in terms of altered reward expectations (e.g., O'Toole et al. 2012), we additionally explored PE coding via model‐based analyses.

Given the importance of the striatum in outcome processing (e.g., Balleine et al. 2007; Samejima et al. 2005), we expected aberrant striatal functioning in OCD (e.g., Burguiere et al. 2015) to interfere with learning and feedback processing, particularly for active learning, thus possibly leading to a dissociation between performance monitoring in active and observational learning. Specifically, we expected impaired task performance in OCD to manifest in rigid, perseverative decision‐making (e.g., Moritz et al. 2009). At the neural level, this may be reflected in aberrant processing of negative feedback; although, due to inconsistent findings in previous FRN studies, the anticipated direction of this effect remained more speculative. If overactive error processing extends to the monitoring of external feedback, this should be expressed in enhanced, i.e., more negative FRN amplitudes to punishment feedback, whereas reduced striatal involvement in observational learning (e.g., Kobza et al. 2012) may lead to preserved, i.e., comparable FRN amplitudes between OCD patients and healthy controls. On the other hand, if the performance monitoring system in OCD is generally disrupted, patients should show similarly altered FRN amplitudes both during active and observational learning. Regarding response evaluation in active learning and consistent with findings by Riesel (2019) showing ERN enhancement only during tasks involving response conflict, we hypothesized that OCD patients would show comparable ERN/CRN amplitudes relative to healthy controls.

Given shared neural mechanisms in OCD and SAD, we hypothesized task performance to be similarly impaired in SAD and OCD patients. Moreover, as both disorders have been linked to overactive error processing (Endrass et al. 2014), we expected comparable amplitudes of the ERN/CRN. Accordingly, non‐differences between groups may also be plausible for the FRN. Alternatively, OCD and SAD patients may be differently sensitive to positive and negative feedback. Thus, while SAD patients may be particularly impaired in reward processing, possibly reflected in enhanced FRN amplitudes to positive feedback (e.g., Cao et al. 2015), OCD patients may rather show alterations in punishment processing.

## 1 Materials and Methods

### 1.1 Participants

The required sample size was estimated based on previous studies that tested a group of OCD patients together with a clinical as well as a healthy control group (e.g., Endrass et al. 2014 with 24 participants per group). Accordingly, we aimed to test a sample of approximately 30 participants per group, which we assumed to have sufficient statistical power even in case of dropouts or exclusions. In total, we recruited 90 volunteers between 18 and 65 years of age. OCD and SAD patients were recruited from the Psychotherapeutic Institute Outpatient Clinic at the Heinrich Heine University Düsseldorf, Germany, the Outpatient and Day Clinic at the Department of General Psychiatry 2 at the University Hospital Düsseldorf, Germany, and an independent collaborating psychotherapy practice in Krefeld, Germany. Further patients were recruited through regional self‐help groups and through disorder‐specific online forums and counseling platforms. Data exclusion (for details, see Supplement) left a sample of 83 participants including 27 HCs, 27 OCD patients and 29 SAD patients (see Table 1).

**TABLE 1:** Demographic and clinical characteristics of study participants after exclusions (*N* = 83).

	HC (*n* = 27)	OCD (*n* = 27)	SAD (*n* = 29)
Demographics			
Age (in years)	32.93 (12.40)	32.37 (9.88)	31.55 (11.34)
Sex (*n* female:male)	15:12	16:11	23:6
Handedness (*n* right:left:ambidextrous)	23:3:1	24:2:1	27:1:0^a^
Clinical features			
Verbal IQ^b^	110.52 (14.92)	114.52 (12.04)	113.62 (17.19)
Comorbidities (*n* with:without a history of comorbid disorders)^c^	—	20:7	21:8
Medication status (*n* medicated:unmedicated)^d^	—	18:9	14:15
BDI‐II	3.00 (4.54)	17.52 (9.68)	16.52 (10.59)
OCI‐R	7.67 (6.58)	28.37 (14.50)	13.24 (9.85)
OBQ‐D	2.93 (0.87)	4.38 (1.16)	3.87 (1.10)
Y‐BOCS	—	21.15 (7.65)	—
LSAS	—	—	83.66 (21.44)

*Note:* Data are presented as means with standard deviations (SD) in parentheses (except for sex, handedness, comorbidities and medication status, which are presented as count data). All data were obtained from self‐report.

Abbreviations: BDI‐II=Beck Depression Inventory‐II; HC=healthy controls; LSAS=Liebowitz Social Anxiety Scale; OBQ‐D=Obsessive‐Beliefs Questionnaire; OCD=obsessive–compulsive disorder; OCI‐R=Obsessive–Compulsive Inventory‐Revised; SAD=social anxiety disorder; Y‐BOCS=Yale‐Brown Obsessive–Compulsive Scale.

^a^ Missing data on handedness for one patient in the SAD group.

^b^ Verbal IQ was estimated using the Mehrfachwahl‐Wortschatz‐Test B (MWT‐B).

^c^ Detailed overview about the history of comorbid disorders can be obtained from Table S1 in the Supplement.

^d^ Detailed overview about the current use of psychotropic medication can be obtained from Table S2 in the Supplement.

Patients had either a primary diagnosis of OCD or SAD, according to self and clinical judgment, as assessed with the Structured Clinical Interview for DSM‐5—Clinician Version SCID‐5‐CV; First et al. 2016, German Version: (Beesdo‐Baum et al. 2019). Accordingly, patients with comorbid OCD and SAD were not included in the present study. Further exclusion criteria for clinical groups were a lifetime history of substance use or bipolar disorder as well as psychotic symptoms. Twenty OCD patients and 21 SAD patients reported a history of at least one comorbid mental disorder. Moreover, 18 OCD and 14 SAD patients were receiving current psychopharmacological treatment. Details on comorbid diagnoses and prescribed (psychotropic) medications are provided in Tables S1 and S2. HCs were only eligible to participate if they were not taking any psychotropic medication and did not report a history of any mental or neurological disorders (including OC or social anxiety symptoms) as assessed by a customized abbreviated version of the SCID‐5. Furthermore, as previous studies showed altered performance monitoring in asymptomatic first‐degree relatives of OCD patients (e.g., Cavedini et al. 2010; Chamberlain et al. 2007; Riesel et al. 2011), only individuals with no family history of OCD were included in the HC group.

All participants had normal or corrected‐to‐normal vision and were naïve to the study's intent. The study procedures conformed to the Declaration of Helsinki and received ethical approval by the Ethics Board of the Faculty of Mathematics and Natural Sciences at Heinrich Heine University Düsseldorf, Germany. Written informed consent was obtained from all participants prior to participation. Participants were paid monetary compensation (50€).

### 1.2 Self‐Report Questionnaires

All participants completed a short multiple‐choice German vocabulary test (Mehrfachwahl‐Wortschatz‐Test B, MWT‐B; Merz et al. 1975) that allowed estimating (verbal) intelligence (Lehrl et al. 1995). Furthermore, the Beck Depression Inventory II (BDI‐II; Beck et al. 1996; German Version: Hautzinger et al. 2006) was administered to assess depressive symptoms. Obsessive–compulsive symptoms and beliefs were assessed in all participants using the Obsessive–Compulsive Inventory Revised (OCI‐R; Foa et al. 2002; German Version: Gönner et al. 2008) and the abbreviated German version of the Obsessive‐Beliefs Questionnaire (OBQ‐D; Ertle et al. 2008; original version: Obsessive Compulsive Cognitions Working Group 2001). Symptom severity in OCD patients was assessed using a self‐report version of the Yale‐Brown Obsessive Compulsive Scale (Y‐BOCS; Goodman et al. 1989; German Version: Hand and Büttner‐Westphal 1991). In SAD patients, symptom severity was assessed using the self‐report version of the Liebowitz Social Anxiety Scale (LSAS; Liebowitz 1987; German Version: Stangier and Heidenreich 2003).

### 1.3 Experimental Task and Procedure

**FIGURE 1:**
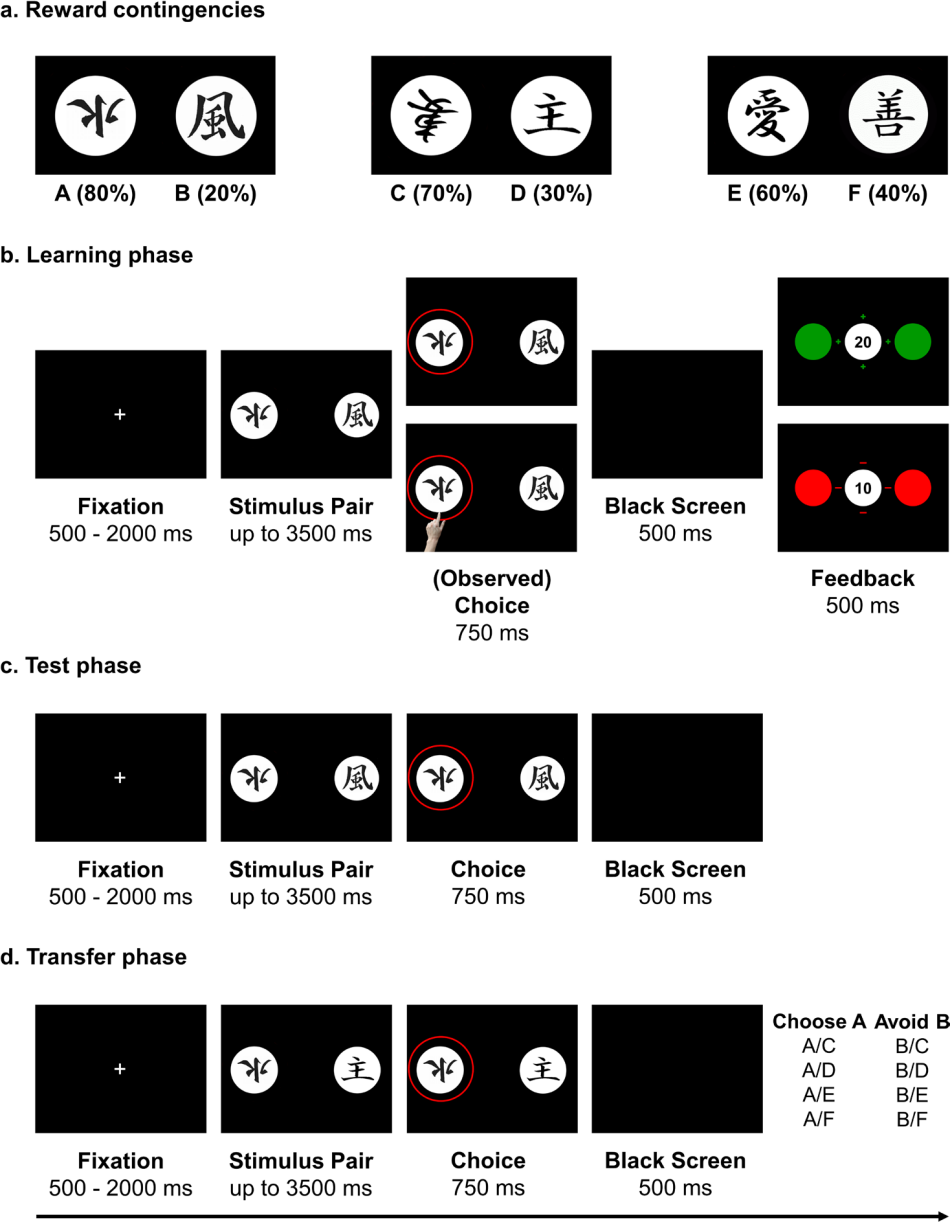
Probabilistic learning task. (a) Reward contingencies for each stimulus in the probabilistic learning task. On each trial, selecting the stimulus with the higher reward contingency, i.e., higher probability to receive positive feedback, was considered the correct response. (b) Trial sequence in the learning phase. In the active learning condition, participants had to choose stimuli and received performance feedback for self‐performed choices. In the observational learning condition, participants observed choices made by another participant, as indicated by a hand symbol, and the corresponding feedback for the observed choices. (c) Trial sequence in the test phase. In the test phase both participants in the active and observational learning condition had to actively choose between stimuli, based on the knowledge gained from the preceding learning phase. However, no feedback was shown for the choices made during the test phase. (d) Trial sequence in the transfer phase. The transfer phase was nearly identical to the test phase; however, new stimulus pairs were presented. Specifically, stimulus A (with the highest reward probability) and stimulus B (with the lowest reward probability) were combined with all remaining stimuli to assess the propensity to seek reward (by choosing A) and to avoid punishment (by avoiding B).

Participants completed two versions of a probabilistic feedback learning task (Frank et al. 2004; see Bellebaum, Rustemeier, et al. 2012 for the extended version used in this study): an active task, where they learned from feedback on their own choices, as well as an observational task, where they learned from vicarious feedback on another participant's choices. Both tasks were as closely matched as possible (see Figure 1) and comprised 400 trials, including four learning blocks of 60 trials, alternating with four test blocks of 30 trials, plus a 40‐trial transfer phase. Crucially, the observational task differed from the active task only during learning. Thus, during test blocks and the transfer phase, the active and observational tasks were fully identical. Note that this study primarily focused on performance in learning and test blocks, with details on the transfer phase provided in the Supplement.

In learning blocks, on each trial, participants were presented with one of three stimulus pairs (A/B, C/D, E/F) consisting of modified Japanese characters, shown on the left and right sides of a computer screen. Each stimulus was associated with a unique reward probability, while within stimulus pairs, reward probabilities were kept opposing and reciprocal (i.e., A/B = 80%/20%, C/D = 70%/30%; E/F = 60%/40%). In the active task version, participants had to actively choose one character by pressing the left or right Ctrl key on a standard USB keyboard, whereas in the observational task version, they had to observe choices made by a previous participant. The task was fully computerized so that observers did not observe another participant's task performance in their physical presence. Instead, a previous active learner's choices were presented virtually, indicated by the picture of a hand in a yoked design. Once active or observed learners indicated their decision, the chosen character was shortly highlighted by a surrounding red circle. After a short delay, participants were provided with performance feedback indicating that they had either gained 20 points (*win*) or lost 10 points (*loss*). Test blocks required active responding from both active and observational learners. Trial structure in test blocks closely resembled that of learning blocks, although trials ended after choice selection, without feedback being presented. Still, participants were encouraged to continue choosing the characters they predicted would lead to reward and avoid those they expected would lead to punishment, as their current score would still be updated and revealed at the end of each test block. Finally, in the transfer phase, both active and observational learners had to choose between novel stimulus pairs combining stimulus A and B with all other stimuli they had not previously been paired with (Figure 1d; but see Supplement for details).

Participants were instructed to learn the relationship between stimulus selection and feedback by evaluating feedback related to their own or observed choices. Since in learning trials during observational learning participants did not actively choose stimuli, they were instructed that the observed participant collected points for both themselves and the observing participant, thus in a cooperative manner. Conversely, for the test (and transfer phase) participants collected points only for themselves. To keep participants motivated during learning, they were instructed that they would need to use the knowledge gained during learning to guide decision‐making in subsequent test blocks. To specifically ensure attentiveness during observational learning, we implemented catch trials after randomly selected trials, requiring observers to indicate which stimulus the observed participant had chosen or whether they had gained or lost points in the immediately preceding trial.

Task versions were held in separate experimental sessions approximately 1 week apart, and participants were randomly assigned to start as an active or observational learner. To preclude stimulus–outcome associations from the first session to bias task performance in the second session, a different stimulus set was introduced in the second session. Participants were tested in the Department of Biological Psychology at Heinrich Heine University Düsseldorf, Germany. However, note that two OCD patients of the final sample were tested at a collaborating psychotherapeutic practice in Krefeld, Germany. The completion of each task took approximately 30 min, including 10 practice trials at the beginning of each task. Stimulus presentation was controlled using Presentation software (Version 20.1, Neurobehavioral Systems Inc., Albany, CA, USA).

### 1.4 EEG Acquisition and Data Processing

#### 1.4.1 EEG Acquisition

EEG data were acquired from 29 scalp sites, i.e., FCz (used as online reference), F7, F3, Fz, F4, F8, FC5, FC1, FC2, FC6, T7, C3, Cz, C4, T8, CP5, CP1, CP2, CP6, P7, P3, Pz, P4, P8, PO9, PO10, O1, Oz, and O2, via active silver/silver‐chloride electrodes that were attached to an elastic textile electrode cap (actiCAP; Brain Products GmbH, Gilching, Germany) according to the extended 10–20 system (Chatrian et al. 1985, 1988). The ground electrode was placed at electrode site AFz, and two additional electrodes were attached, one to each mastoid. Horizontal and vertical electro‐oculograms (EOGs) were recorded with one electrode attached lateral to the left outer canthus (hEOG), and one electrode placed above the left eye (vEOG), respectively. Impedances were kept below 20 kΩ. EEG data were amplified using a Brain Amp DC amplifier (Brain Products GmbH, Gilching, Germany) and recorded via BrainVision Recorder software (version 1.20.0506, Brain Products GmbH, Gilching, Germany) with a sampling rate of 1000 Hz and an online low‐pass filter of 100 Hz on a Windows workstation.

#### 1.4.2 Preprocessing

EEG data were preprocessed offline using BrainVision Analyzer software (version 2.2.0, Brain Products GmbH, Gilching, Germany). First, for each participant, the raw EEG data were visually inspected to identify electrodes with excessive noise or flat lines. If present, the signal of these channels was interpolated (active task: mean number of channels interpolated = 0.31, SD = 0.68, range = 0–3; observational task: mean number of channels interpolated = 0.30, SD = 0.66, range = 0–3). Next, to restore the signal from electrode FCz, EEG data were re‐referenced to the average signal of the mastoid channels. Data were then filtered for frequencies below 0.1 Hz and above 30 Hz. Additionally, we applied a 50 Hz notch filter. Signal portions contaminated by ocular artifacts were corrected using an independent component analysis (ICA) based ocular correction approach implemented in the BrainVision Analyzer software (Plank 2013). Subsequently, the continuous EEG data were segmented into epochs spanning the time from 200 ms before to 600 ms after feedback (win or loss) onset. Similarly, we additionally extracted response‐locked (correct or incorrect) epochs for active learning data with a duration of 800 ms (including a 200‐ms pre‐response interval). For both feedback and response‐locked epochs, baseline correction was applied based on the pre‐stimulus or pre‐response period.

Epochs in which sampling points exceeded the minimal or maximal allowed amplitude of ±100 μV or exhibited activity below 0.5 μV (within 100 ms), a voltage difference of 200 μV (within 200 ms), or voltage steps > 50 μV between consecutive sampling points were removed during artifact rejection (for details on mean percentage of removed segments, see Table S3). The remaining, artifact‐free segments of electrodes Fz, FCz, FC1, FC2, and Cz were exported for further processing in MATLAB (version R2020a; The MathWorks Inc., Natick, Massachusetts, USA).

#### 1.4.3 Scoring Procedure

For the ERP analyses, we opted for a trial‐level approach (see e.g., Volpert‐Esmond et al. 2018). In line with our research question, we therefore focused on the feedback‐locked FRN in active and observational learning, whereas the response‐locked ERN/CRN was explored in active learning only. Both FRN and ERN/CRN were scored in a predefined fronto‐central electrode cluster (Fz, FCz, FC1, FC2, Cz). The scoring procedure was based on a hierarchical data‐driven approach (for a similar procedure, see, e.g., Rodrigues et al. 2024). Importantly, visual inspection of the feedback‐locked grand average (GA) waveforms indicated that the observer FRN was slightly delayed. Hence, in order to allow for adequate assessment of the FRN, this procedure was applied separately for active and observational learning data.

For both response‐ and feedback‐locked ERPs, we first computed a composite waveform (COMP) by calculating the mean of the condition‐wise GAs of the pooled signal from the predefined (fronto‐central) electrode cluster, for example:

$${\text{COMP}}_{\text{feedback}-\text{locked}}=\frac{\text{pooled}\;{\mathrm{GA}}_{\mathrm{win}}+\text{pooled}\;{\mathrm{GA}}_{\text{loss}}}{2}$$

Thus, this composite waveform represented the *condition‐unweighted average signal*, ensuring that conditions were weighted equally. Next, the composite waveform was used to determine each component's peak latency—i.e., the local minimum (due to the negative polarity of both FRN and ERN/CRN)—within a predefined, literature‐backed a priori search window. These windows were 200–350 ms post‐feedback for the FRN (Faßbender et al. 2023), and 0–100‐ms post‐response for the ERN/CRN (Gehring et al. 2018). ERP components were then scored as the mean amplitude in an extraction time window ±30 ms around the identified peak for each electrode within its (fronto‐central) cluster in each trial. Accordingly, the FRN was scored as the mean amplitude within 231–291 ms following feedback for active learning, and 241–301 ms for observational learning. The ERN/CRN was scored as the mean amplitude within −11‐49 ms relative to response onset.

### 1.5 Statistical Analyses

All statistical analyses were performed using R statistical software (version 4.1.0; R Core Team 2021). We opted for a trial‐level approach using (generalized) linear mixed‐effects models ([G]LMMs). Binary choice behavior was analyzed using GLMMs implemented in the R package *afex* (Singmann et al. 2022). LMMs as implemented in the *lme4* package (Bates et al. 2015) were used for the analysis of single‐trial ERP data. Contrast weights for all categorical predictors were deviation coded (e.g., −0.5, 0.5) and all continuous predictors were centered around zero such that the intercept represented the grand mean. Importantly, when using (G)LMMs for categorical factors with more than two levels (e.g., diagnostic group), main and interaction effect terms are calculated as *n‐1* comparisons (where *n* is the number of factor levels), dependent on a predefined reference level (Clopper 2013). These planned contrasts are preferable to traditional omnibus tests as they directly incorporate the hypotheses of interest into the statistical model (Hays 1978; Schad et al. 2020). Given our focus on OCD‐related alterations in performance monitoring, all group‐related main and interaction effects were therefore estimated in reference to OCD (i.e., in comparison to HCs and SAD).

For all models, we attempted to incorporate the maximum possible random‐effects structure, including random intercepts and slopes for all within‐participant main and interaction effects as well as their correlation (Barr et al. 2013). For EEG analyses, a random intercept by electrode site was also included. In case of non‐convergence or singular fit, we used the *buildmer* package (Voeten 2020) to find the maximal model that still converged. The threshold for defining statistically significant results was set to *p* < 0.050. *P*‐values for GLMMs were calculated via likelihood ratio tests (LRT) based on type III sums of squares, and for LMMs they were based on Satterthwaite approximated degrees of freedom implemented in the *lmerTest* package (Kuznetsova et al. 2017). Significant interaction effects were resolved using the *emmeans* package (Lenth 2022). Adjusted *p*‐values for multiple comparisons were obtained by controlling the false‐discovery rate (FDR; Benjamini and Hochberg 1995). Prior to model fitting, we removed invalid trials, including no‐response trials as well as trials with rushed responses (< 200 ms) resulting in the exclusion of 1.91% of trials.

#### 1.5.1 Choice Behavior

##### 1.5.1.1 Task Performance in Test Trials

To examine learning performance in the experimental task, we conducted GLMM analysis on binary single‐trial choice accuracy data (0 = incorrect, 1 = correct). Choice accuracy was defined as selecting the stimulus with the higher reward probability. Given that choices were merely observed but not performed in learning blocks during observational learning, this analysis was restricted to test trials. Categorical fixed‐effect predictors included the between‐subjects factor group (*OCD [=reference], HC, SAD*) and the within‐subjects factor agency (*active [=reference], observational*). Reward contingency for each stimulus pair (A/B: 80%, C/D: 70%, E/F: 60%, coded as −1, 0, 1) was implemented as a continuous predictor. Furthermore, to assess learning‐related effects of task duration, we included block (1–4) as an additional continuous predictor, centered around 2.5 (corresponding to the midpoint of the total number of test blocks). The GLMM was specified according to the following Wilkinson notation formula:

$$\text{Choice Accuracy}∼\text{Group}*\text{Agency}*\text{Contingency}*\text{Block}+\left(1+\text{Agency}*\text{Contingency}*\text{Block}|\text{Participant}\right)$$

##### 1.5.1.2 Win‐Stay/Lose‐Shift Behavior in Learning Trials

Choice shift (0 = stay, 1 = shift) was set as the dependent variable in the GLMM analysis of win‐stay/lose‐shift behavior. Since feedback was only presented during learning trials, and active responding was only required in active learning, this analysis was restricted to active learning trials. Here, choice shifting reflected whether participants maintained (*stay*) or switched (*shift*) their choice compared to their previous choice when encountering the same stimulus pair again. To this end, we included the between‐subjects factor group (*OCD [=reference], HC, SAD*) and the within‐subjects factor previous feedback valence (*win [=reference], loss*) as categorical fixed‐effect predictors. Due to the probabilistic nature of the task, changing the response strategy following loss feedback and maintaining the response strategy following win feedback was not always adaptive. Therefore, and motivated by Kirschner et al. (2024), we additionally included the categorical fixed‐effect predictor previous feedback authenticity (*authentic [=reference]*, *misleading*), allowing us to distinguish between authentic (i.e., feedback that aligns with the choice accuracy) and misleading feedback (i.e., probabilistic deviations in feedback, thus not in alignment with the choice accuracy), which, depending on feedback valence, required opposite adjustments (i.e., shifting following authentic loss or misleading win feedback as well as staying following authentic win or misleading loss feedback). Again, block (1–4) was included as a continuous predictor, centered around 2.5. The GLMM was specified as the following Wilkinson notation formula:

$$\text{Choice Shift}∼\text{Group}*\text{Previous Feedback Valence}*\text{Previous Feedback Authenticity}*\text{Block}+\left(1+\text{Previous Feedback Valence}*\text{Block}|\text{Participant}\right)$$

#### 1.5.2 EEG

##### 1.5.2.1 Feedback‐Related Negativity (FRN)

LMM analysis on the feedback‐locked FRN component used single‐trial FRN amplitudes as the dependent variable. Categorical fixed‐effect predictors included the between‐subjects factor group (*OCD [=reference], HC, SAD*), and the within‐subjects factors agency (*active [=reference], observational*) and feedback valence (*win [=reference], loss*). The model formula in Wilkinson notation was:

$${\mathrm{FRN}}_{\mathrm{amp}}∼\;\text{Group}*\text{Agency}*\text{Feedback Valence}+\left(1+\text{Agency}*\text{Feedback Valence}\right|\;\text{Participant})+\left(1|\text{Electrode}\right)$$

##### 1.5.2.2 Error‐ and Correct‐Related Negativity (ERN/CRN)

LMM analysis for the response‐locked ERN/CRN in active learning was conducted with single‐trial ERN/CRN amplitudes set as the dependent variable. Categorical fixed‐effect predictors included the between‐subjects factor group (*OCD [=reference], HC, SAD*) and the within‐subjects factor choice accuracy (*correct [=reference], incorrect*). Additionally, on each trial and for each participant and stimulus pattern, we examined the current learning status, i.e., whether the participant had sufficiently learned the reward contingency. To this end, we first determined when participants first exceeded a mean choice accuracy of 90% in the preceding 10 trials for each stimulus pair separately, using a sliding window approach. Once this initial criterion was met, we then ensured that learning was stable by checking whether the mean choice accuracy for the remaining trials remained above 65%. Crucially, we assumed that participants had sufficiently learned the reward contingency for that stimulus pair only if learning was stable for the remainder of the task. If learning was not stable, we searched for the next potential breakpoint. Once a valid breakpoint was identified, trials were divided into a pre‐ and post‐learning phase. Note, however, that by applying these criteria, some participants did not sufficiently learn the contingencies for all stimulus pairs. For these instances, all trials of these specific pairs were assumed to persist in the pre‐learning phase. Learning status (*pre‐learning [=reference]*, *post‐learning*) was finally entered as an additional categorical predictor in the LMM analysis. The LMM was specified according to the following Wilkinson notation formula:

$$\begin{array}{c} \mathrm{ERN}⁄{\mathrm{CRN}}_{\mathrm{amp}}∼\text{Group}*\text{Choice}\;A\text{ccuracy}*\text{Learning}\;S\text{tatus}\\ +\left(1+\text{Choice}\;A\text{ccuracy}*\text{Learning}\;\text{Status}\right|\;\text{Participant})+\left(1|\text{Electrode}\right) \end{array}$$

#### 1.5.3 Exploratory Analyses

##### 1.5.3.1 Model‐Based ERP Analyses

###### 1.5.3.1.1 Computational Modeling of Behavioral Data

To ensure that potential processing differences were not solely driven by differences in learning performance, we aimed to explore the relationship between participants' underlying reward expectations and the FRN and ERN/CRN. To obtain single‐trial estimates of participants' reward expectations and PEs, we fitted different reinforcement learning (RL) models to each participant's choice sequence and reinforcement history (Sutton and Barto 2018; Watkins and Dayan 1992). Models were fitted using the *fmincon* function implemented in the Optimization Toolbox in MATLAB (see, e.g., Raab and Hartley 2020) and compared using the Bayesian information criterion (BIC).

For a comparable fitting procedure and assessment of reward expectancy in both active and observational learning, BIC scores were based on the negative loglikelihood (NLL) to identify the model that best predicted actual choice behavior in the test and transfer phases (as motivated by Schultner et al. 2024), from which we eventually extracted latent reward expectancies (*Q*‐values) and PE estimates. Models were fitted separately to active and observational learning data, though the winning model for both active and observational learning included separate learning rates for positive $\left(\alpha^{+}\right)$ and negative feedback ($\alpha^{-}$) as well as an inverse temperature parameter (β). Additionally, the winning model contained a choice‐induced preference change (CIPC) parameter (ε) used to update *Q*‐values even in the absence of external feedback in test trials, consistent with recent findings suggesting internal value updating when feedback is not available (e.g., Ptasczynski et al. 2022). Note that this model outperformed models which assumed static or decaying action values in the absence of feedback. For further details including model validation with parameter identifiability and recovery, see the Supplement.

###### 1.5.3.1.2 Exploratory EEG Analyses

For the model‐based FRN analysis, we extended the LMM formula reported for the model‐free analysis by including the absolute value of the PE as a continuous within‐subjects predictor. This unsigned PE hence indexes the general unexpectedness or surprise of the obtained performance feedback, and has thereby also been referred to as a salience PE (SPE; Heydari and Holroyd 2016; Talmi et al. 2013). Thus, including both the SPE and feedback valence allowed us to disentangle whether the FRN signaled general unexpectedness (in this case to be reflected in a main effect of SPE) or a signed, i.e., positive and/or negative, PE (in this case to be reflected in an SPE × feedback valence interaction effect). SPE values were centered around zero, with negative values indicating expected and positive values indicating unexpected feedback. The LMM was specified using the following Wilkinson notation formula:

$${\mathrm{FRN}}_{\mathrm{amp}}∼\text{Group}*\text{Agency}\times \text{Feedback Valence}*\mathrm{SPE}+\left(1+\text{Agency}*\text{Feedback Valence}*\mathrm{SPE}\right|\;\text{Participant})+\left(1|\text{Electrode}\right)$$

Model‐based ERN/CRN analysis was based on the difference in *Q*‐values for the chosen and unchosen option ($\Delta Q=Q_{c}-Q_{\bar{c}})$. Accordingly, ΔQ indexes *predictive accuracy* where positive values indicate internally anticipated correct choices (i.e., choosing the option with the higher reward expectation) and negative values indicate internally anticipated incorrect choices (i.e., choosing the option with the lower reward expectation). Thus, continuous ΔQ replaced the categorical predictor *objective* choice accuracy, but should predict ERN/CRN amplitude regardless of the current learning status. As ΔQ was already meaningfully centered around zero (0 = equal reward expectations for both options), we did not need to further transform ΔQ before model fitting. The model formula in Wilkinson notation was:

$$\mathrm{ERN}/{\mathrm{CRN}}_{\mathrm{amp}}∼\text{Group}*\Delta Q*\text{Learning Status}+\left(1+\Delta Q*\text{Learning Status}\right|\;\text{Participant})+\left(1\;|\;\text{Electrode}\right)$$

##### 1.5.3.2 Transdiagnostic‐Dimensional Analyses

We further explored OCD specificity by conducting transdiagnostic‐dimensional (G)LMM analyses based on OCD symptom severity. Specifically, all (G)LMMs that showed significant group effects were re‐fitted, replacing the categorical predictor group with the continuous predictor OCI‐R. The OCI‐R was chosen because it measures OC symptoms relatively independently from symptoms of depression, anxiety, worry, and perfectionism (Gönner et al. 2008). Transformed scores were then centered around the sample mean prior to model fitting. Comparable analyses using the BDI‐II as an alternative dimensional predictor are reported in the Supplement.

## 2 Results

### 2.1 Choice Behavior

Detailed results of all GLMM analyses as well as all parameter estimates and inferential statistics are provided in Tables S4–S6. Exploratory results on transfer phase performance as well as on the modulating role of depressive symptoms on choice behavior can be found in the Supplement.

#### 2.1.1 Task Performance in Test Trials

**FIGURE 2:**
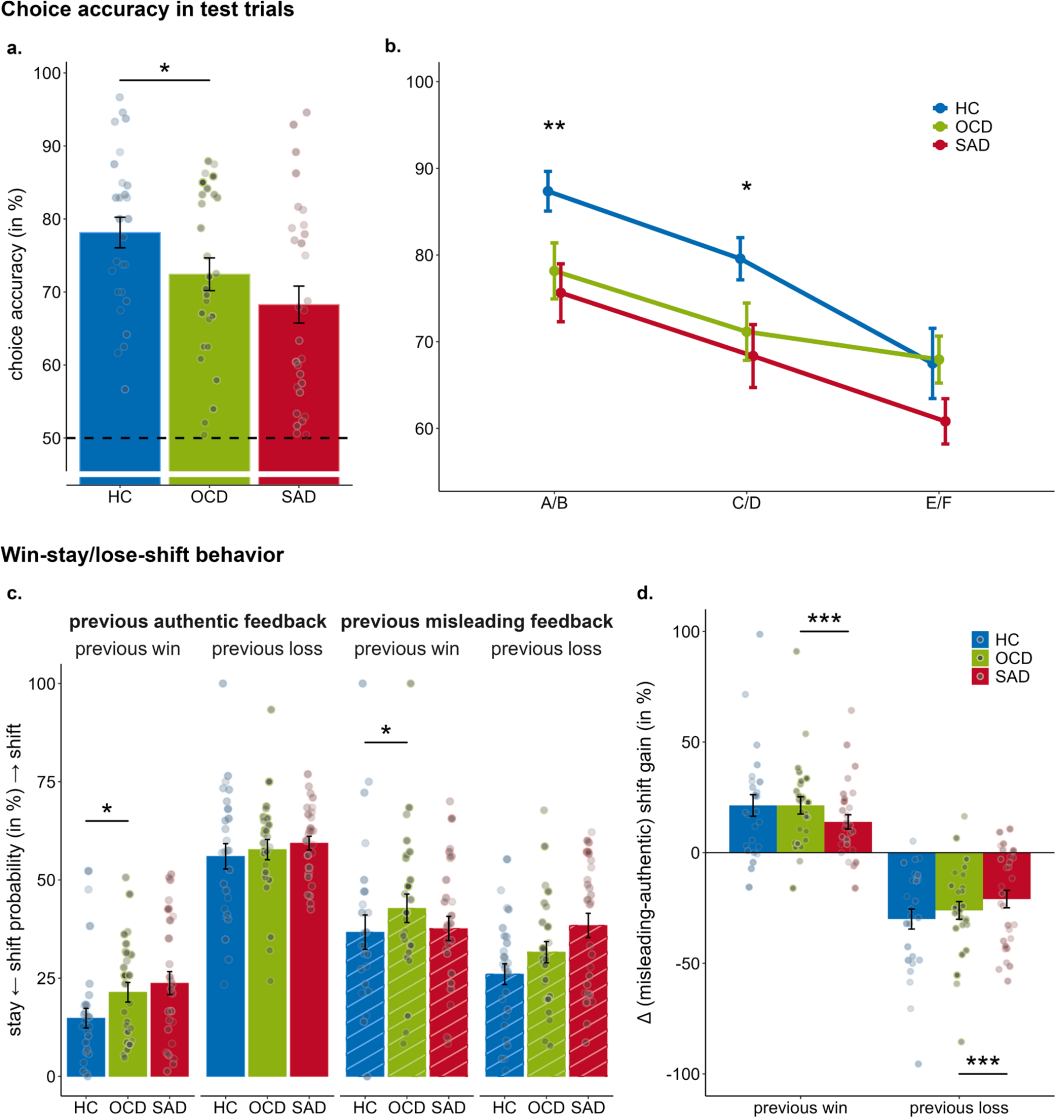
Choice behavior. (a) and (b) Show learning performance in test blocks. Plots (c) and (d) show win‐stay/lose‐shift behavior in learning blocks. Plots show aggregated empirical means and individual data points, and are therefore presented on the response scale. Darker shaded individual data points indicate higher OCD symptom severity, that is, higher OCI‐R scores. Asterisks indicate significant group differences as revealed by planned group contrasts (OCD vs. HC; OCD vs. SAD) in generalized linear mixed‐effects model analyses. Error bars represent the standard error of the mean. Abbreviations: HC=healthy controls; OCD=obsessive–compulsive disorder; SAD=social anxiety disorder. *p < 0.050, **p < 0.010, ***p < 0.001.

Task performance was assessed by analyzing choice accuracy in test trials. Results revealed significant main effects of block, *z* = 5.19, *p* < 0.001, *b* = 0.24 (95% CI = 0.15 to 0.33), and contingency, *z =* −7.66, *p* < 0.001, *b* = −0.71 (95% CI = −0.89 to −0.53), indicating that choice accuracy linearly increased across blocks and across reward contingencies. Planned contrasts suggested task performance was generally impaired in OCD patients relative to HCs, *z* = 2.47, *p* = 0.014, *b* = 0.73 (95% CI = 0.15 to 1.31), whereas no difference was found in learning performance between OCD and SAD patients (*p* = 0.734; see Figure 2a). Moreover, planned contrasts for the group × contingency interaction effect showed a larger contingency effect in HCs compared to OCD, *z* = 2.46, *p* = 0.014, *b* = −0.56 (95% CI = −1.01 to −0.11; see Figure 2b). Specifically, while both OCD patients and HCs performed similarly when reward contingency was low (E/F), *z* = 0.60, *p*_adj_ = 0.549, *b* = 0.17 (95% CI = −0.39 to 0.73), there were increasing differences for medium (C/D), *z* = 2.47, *p*_adj_ = 0.021, *b* = 0.73 (95% CI = 0.15 to 1.31), and high (A/B) reward contingency, *z* = 2.90, *p*_adj_ = 0.011, *b* = 1.29 (95% CI = 0.42 to 2.17). Again, for the comparison of OCD and SAD patients, planned interaction contrasts did not yield significant results (*p* = 0.227).

Transdiagnostic‐dimensional GLMM analysis mirrored the results reported above. Interestingly, there was a significant OCI‐R × contingency interaction effect, *z* = 2.09, *p* = 0.037, *b* = 0.01 (95% CI = 0.00 to 0.03), suggesting that the higher the reward contingency, the more strongly OCI‐R scores affected learning performance by (descriptively) predicting lower choice accuracy.

#### 2.1.2 Win‐Stay/Lose‐Shift Behavior in Learning Trials

To test whether impaired task performance was due to maladaptive choice perseveration, we analyzed win‐stay/lose‐shift behavior in active learning trials. Results yielded significant main effects of block, *z* = −3.49, *p* < 0.001, *b* = −0.13 (95% CI = −0.20 to −0.06) and previous feedback valence, *z* = 10.30, *p* < 0.001, *b* = 0.86 (95% CI = 0.70 to 1.02), suggesting generally decreased shifting with task progression and overall increased shifting behavior following losses compared to wins, thus confirming win‐stay/lose‐shift behavior. Furthermore, results revealed a previous feedback valence × previous feedback authenticity interaction effect, *z* = 21.57, *p* < 0.001, *b* = −1.75 (95% CI = −1.91 to −1.59). Shifting was increased following authentic compared to misleading losses, *z* = −18.10, *p*_adj_ < 0.001, *b* = −0.93 (95% CI = −1.03 to −0.83), but decreased following authentic compared to misleading wins, *z* = 13.08, *p*_adj_ < 0.001, *b* = 0.82 (95% CI = 0.70 to 0.94). Further comparisons showed no differences in choice shifting when feedback was misleading (*p*_adj_ = 0.865). However, increased shifting was observed for losses versus wins when feedback was authentic, *z* = 20.19, *p*_adj_ < 0.001, *b* = 1.74 (95% CI = 1.57 to 1.90). Furthermore, there was a significant previous feedback valence × previous feedback authenticity × block interaction effect, *z* = −3.11, *p* = 0.002, *b* = −0.22 (95% CI = −0.37 to −0.08). Shifting behavior progressively decreased for authentic, *z* = −4.08, *p*_adj_ < 0.001, *b* = −0.19 (95% CI = −0.28 to −0.10), but not misleading wins (*p*_adj_ = 0.651), suggesting overall successful learning to adapt to positive feedback. Decreased learning‐related shifting was also found for misleading losses, *z* = −4.03, *p*_adj_ < 0.001, *b* = −0.18 (95% CI = −0.27 to −0.09), as well as decreased maladaptive shifting for authentic losses, *z* = −2.72, *p*_adj_ = 0.009, *b* = −0.12 (95% CI = −0.20 to −0.03).

Most importantly, choice shifting differed between groups, with planned contrasts showing generally increased shifting—not perseveration—in OCD compared to HCs, *z* = −2.48, *p* = 0.013, *b* = −0.47 (95% CI = −0.85 to −0.10), but not SAD (*p* = 0.813). Furthermore, while planned contrasts for the group × previous feedback valence × previous feedback authenticity interaction did not reveal significant differences between OCD and HCs (*p* = 0.500), the interaction coefficient for the feedback valence × previous feedback authenticity effect was increased for OCD compared to SAD, *z* = 3.07, *p* = 0.002, *b* = 0.58 (95% CI = 0.21 to 0.94). To unravel the underlying result pattern, we examined the patients' sensitivity to misleading feedback, calculated as Δ (misleading‐authentic), reflecting how effectively patients adapted to feedback by distinguishing between misleading and authentic feedback. If adjusting effectively, Δ‐scores should be positive for wins and negative for losses where higher absolute values indicate better differentiation between misleading and authentic feedback. Comparing Δ‐scores for wins and losses between patients indicated that SAD patients were more insensitive to misleading feedback. Accordingly, the (absolute) Δ value was decreased in SAD compared to OCD for both previous wins, *z* = −2.21, *p*_adj_ = 0.031, *b* = −0.32 (95% CI = −0.60 to −0.04), and losses, *z* = 2.16, *p*_adj_ = 0.031, *b* = 0.26 (95% CI = 0.23 to 0.49; see Figure 2c,d).

Transdiagnostic‐dimensional GLMM analysis suggested that OCD symptom severity predicted the overall tendency to shift, *z* = 2.14, *p* = 0.032, *b* = 0.01 (95% CI = 0.00 to 0.02). A significant OCI‐R × previous feedback valence interaction effect, *z* = −2.07, *p* = 0.038, *b* = −0.01 (95% CI = −0.02 to −0.00), further revealed that higher OCI‐R scores predicted increased choice shifting only following wins, *z* = 2.34, *p*_adj_ = 0.039, *b* = 0.02 (95% CI = 0.00 to 0.03), but not losses (*p*_adj_ = 0.171).

### 2.2 EEG

**FIGURE 3:**
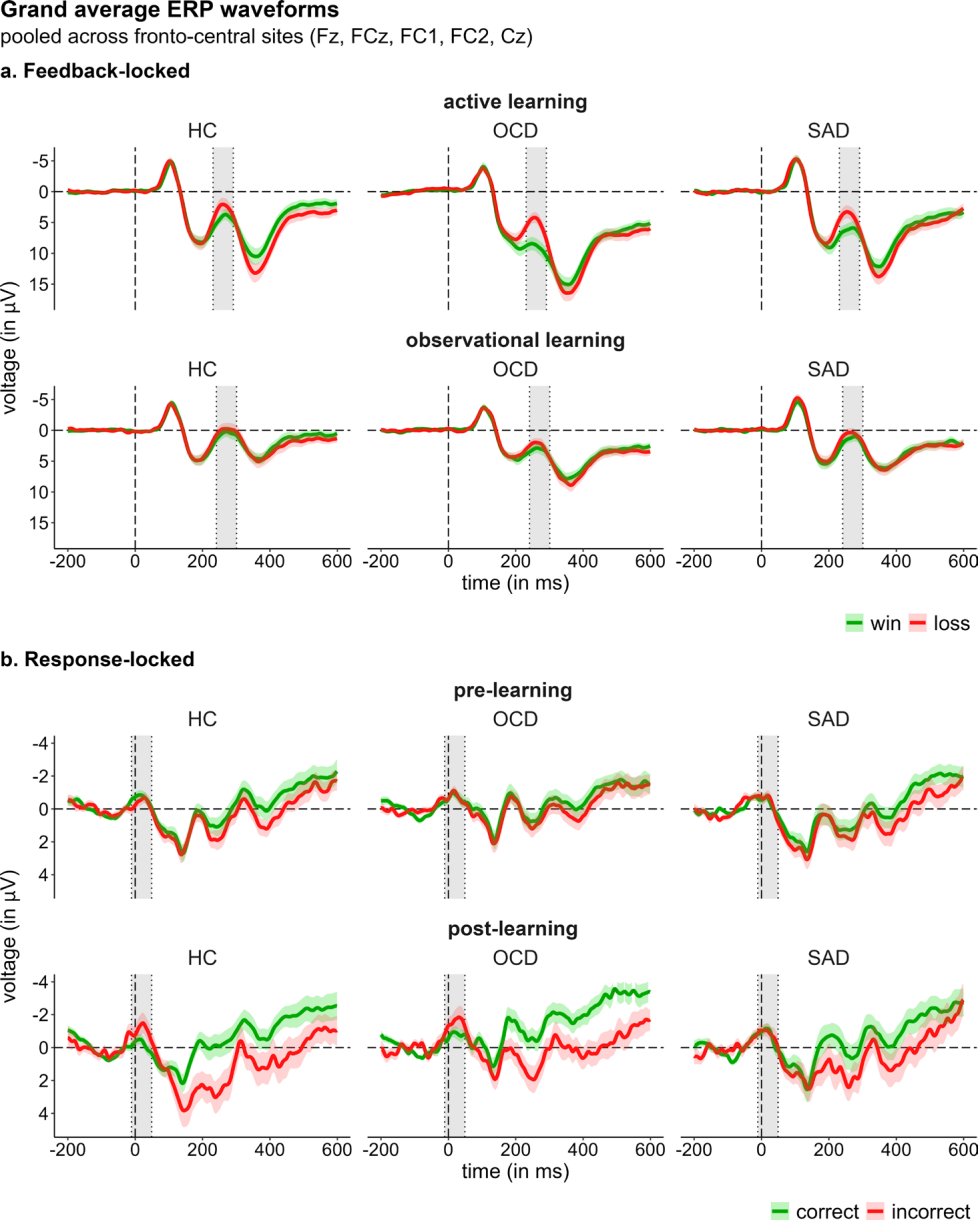
Grand average ERP waveforms pooled across fronto‐central sites. (a) Shows feedback‐locked ERP waveforms separately for active and observational learning. (b) Shows response‐locked ERP waveforms in the pre‐ and post‐learning stage for active learning data. Data represent the mean ± standard error. Zero points on the *x*‐axis refer to feedback or response onset, respectively. Gray‐shaded areas indicate the measurement windows used for scoring the feedback‐related negativity (FRN) as well as the error‐ and correct‐related negativity (ERN/CRN). Abbreviations: ERP=event‐related potential; HC=healthy controls; OCD=obsessive–compulsive disorder; SAD=social anxiety disorder.

Feedback‐ and response‐locked GA ERPs are provided in Figure 3. Detailed LMM results, including all parameter estimates and inferential statistics, are provided in the supplementary Tables S7–S10. Exploratory results on the modulating role of depressive symptoms on feedback processing are provided in the Supplement.

### 2.3 Model‐Free ERP Analyses

#### 2.3.1 Feedback‐Related Negativity (FRN)

Results yielded significant main effects of feedback valence, *t*(77.78) = −6.95, *p* < 0.001, *b* = −1.62 (95% CI = −2.09 to −1.16), and agency, *t*(75.21) = −9.05, *p* < 0.001, *b* = −3.81 (95% CI = −4.64 to −2.97), indicating more negative FRN amplitudes for losses than wins and in observational compared to active learning. Importantly, the significant agency × feedback valence interaction indicated enhanced valence coding (loss‐win) for active compared to observational learning, *t*(77.43) = 5.70, *p* < 0.001, *b* = 1.86 (95% CI = 1.21 to 2.51).

**FIGURE 4:**
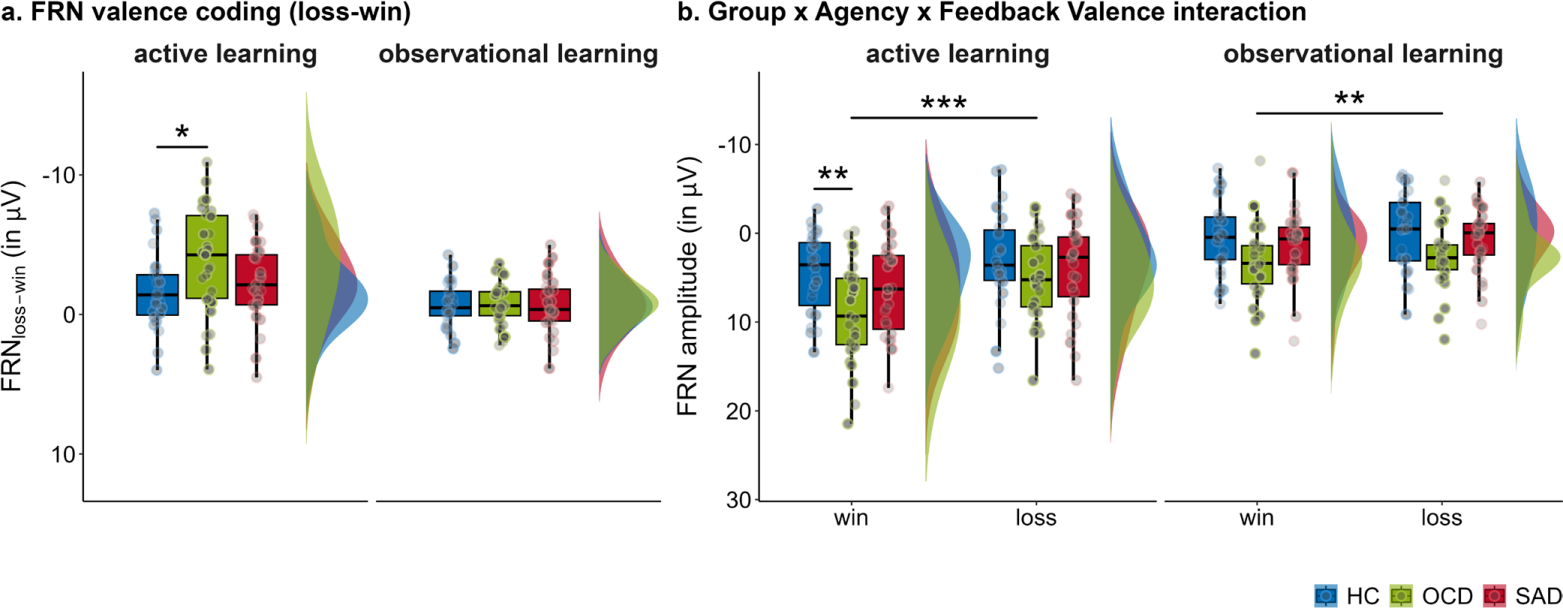
FRN amplitudes. (a) Depicts FRN valence coding. (b) Shows FRN amplitudes separately for win and loss feedback. Plots show aggregated empirical means and individual data points, and are therefore presented on the response scale. Darker shaded individual data points indicate higher OCD symptom severity, i.e., higher OCI‐R scores. Asterisks indicate significant (group) differences as revealed by planned (group) contrasts (i.e., OCD vs. HC; OCD vs. SAD) in model‐free linear mixed‐effects model analysis. Abbreviations: HC=healthy controls; OCD=obsessive–compulsive disorder; SAD=social anxiety disorder. *p < 0.050, **p < 0.010, ***p < 0.001.

Planned contrasts showed that FRN amplitudes were generally attenuated in OCD patients relative to HCs, *t*(51.48) = −4.60, *p* = 0.012, *b* = −2.87 (95% CI = −5.11 to −0.64), but not relative to SAD patients (*p* = 0.109). Moreover, planned interaction contrasts suggested that the agency × feedback valence interaction (i.e., larger loss–win difference for active vs. observational learning) was stronger in OCD patients relative to HCs, *t*(78.10) = 2.61, *p* = 0.011, *b* = 2.10 (95% CI = 0.50 to 3.70), whereas, although descriptively enhanced in OCD compared to SAD, this difference did not reach statistical significance (*p* = 0.059). To further elucidate the effect for the OCD versus HC subsample, we examined simple effects of feedback valence, separately by agency. For active learning, we found larger FRN amplitudes for losses compared to wins in both OCD patients, *t*(78.00) = −6.32, *p*_adj_ < 0.001, *b* = −3.83 (95% CI = −5.04 to −2.63), and HCs, *t*(78.04) = −2.61, *p*_adj_ = 0.011, *b* = −1.59 (95% CI = −2.79 to −0.38), but with enhanced valence coding in OCD patients, *t*(78.02) = 2.62, *p* = 0.011, *b* = 2.25 (95% CI = 0.54 to 3.96; see Figure 4a). Crucially, this was driven by more positive FRN amplitudes in OCD patients relative to HCs for wins, *t*(78.03) = −3.23, *p*_adj_ = 0.004, *b* = −4.55 (95% CI = −7.36 to −1.74; see Figure 4b), while no group differences emerged for losses (*p*_adj_ = 0.107). Conversely, for observational learning, the FRN did not differentiate between wins and losses in either OCD patients or HCs (both *p*_adj_ ≥ 0.061), and valence coding did not significantly differ between groups (*p* = 0.762). Transdiagnostic‐dimensional analysis showed that these FRN modulations were not predicted by OCI‐R scores (*p*s ≥ 0.085).

#### 2.3.2 Error‐ and Correct‐Related Negativity (ERN/CRN)

To investigate response evaluation in active learning, analysis of trial‐level ERN/CRN amplitudes yielded a significant main effect of learning status, *t*(60.34) = −2.03, *p* = 0.047, *b* = −0.27 (95% CI = −0.53 to −0.00), indicating generally more negative ERN/CRN amplitudes post‐ compared to pre‐learning. More interestingly, there was a significant accuracy × learning status interaction effect, suggesting increased differentiation between incorrect and correct choices post‐ versus pre‐learning, *t*(64.13) = −2.01, *p* = 0.049, *b* = −0.59 (95% CI = −1.17 to −0.00). Post‐hoc comparisons with FDR‐correction, conducted separately for pre‐ and post‐learning trials, indicated comparable ERN/CRN amplitudes for incorrect and correct choices pre‐learning (*p*_adj_ = 0.834). However, also post‐learning incorrect choices elicited only marginally larger—though non‐significant—ERN amplitudes compared to correct choices (*p*_adj_ = 0.098). There were no significant group effects (*p*s ≥ 0.211).

### 2.4 Model‐Based ERP Analyses

#### 2.4.1 Feedback‐Related Negativity (FRN)

To examine whether abnormally positive FRN amplitudes for win feedback were driven by impaired learning and correspondingly reduced reward expectations, model‐based FRN analysis was conducted. Adding the SPE as an additional predictor led to an increase in the proportion of variance explained, as indicated by the conditional *R* ^2^ statistic (from 0.225 to 0.280, ΔBIC = 1520). Results showed a significant feedback valence × SPE interaction effect, *t*(67.29) = −2.91, *p* = 0.005, *b* = −3.73 (95% CI = −6.29 to −1.17). Resolving this effect revealed that the FRN signaled a positive, *t*(66.53) = 2.74, *p*_adj_ = 0.011, *b* = 2.24 (95% CI = 0.36 to 4.12) and negative PE, *t*(62.29) = −2.63, *p*_adj_ = 0.011, *b* = −1.49 (95% CI = −2.79 to −0.19) with more positive FRN amplitudes for feedback better than expected and more negative FRN amplitudes for feedback worse than expected. PE coding was modulated by agency, reflected in an agency × feedback valence × SPE interaction effect, *t*(67.22) = 3.13, *p* = 0.003, *b* = 7.52 (95% CI = 2.73 to 12.31). Resolving this effect showed that while for active learning, the FRN coded both a positive, *t*(61.43) = 3.14, *p*_adj_ = 0.003, *b* = 4.33 (95% CI = 1.17 to 7.50), and negative PE, *t*(58.33) = −3.43, *p*_adj_ = 0.002, *b* = −3.16 (95% CI = −5.27 to −1.04), no PE coding was found for observational learning (*p*s_adj_ ≥ 0.847).

As for the model‐free analysis, planned a priori contrasts revealed generally more positive FRN amplitudes in OCD patients compared to HCs, *t*(77.69) = −2.27, *p* = 0.026, *b* = −2.74 (95% CI = −5.14 to −0.34), whereas no differences emerged between OCD and SAD patients (*p* = 0.117). In addition, planned interaction contrasts showed enhanced valence coding in OCD compared with HCs, *t*(69.64) = 2.13, *p* = 0.037, *b* = 1.28 (95% CI = 0.08–2.48), with abnormally positive FRN amplitudes for win, *t*(78.64) = −2.70, *p*_adj_ = 0.017, *b* = −3.38 (95% CI = −5.87 to −0.88), but no difference for loss feedback (*p*_adj_ = 0.093). Interestingly, when controlling for the SPE, planned interaction contrasts additionally indicated enhanced valence coding in OCD relative to SAD, *t*(69.05) = 2.23, *p* = 0.029, *b* = 1.34 (95% CI = 0.14 to 2.53), again driven by more positive FRN amplitudes for win, *t*(77.85) = −2.06, *p*_adj_ = 0.085, *b* = −2.58 (95% CI = −5.07 to −0.09), though FDR‐correction prevented this effect from reaching statistical significance.

When substituting the categorical predictor group by the continuous predictor OCI‐R, results revealed a significant OCI‐R × feedback valence interaction effect, *t*(71.20) = −2.03, *p* = 0.046, *b* = −0.04 (95% CI = −0.07 to −0.00). This interaction suggested that the positive relationship between OCI‐R and FRN amplitude was stronger for wins compared to losses; however, note that individual tests for each outcome type did not show a significant relationship between OCI‐R for neither wins nor losses (*p*_adj_ ≥ 0.198).

#### 2.4.2 Error‐ and Correct‐Related Negativity (ERN/CRN)

To better account for *internal* response valuation, model‐based ERN/CRN analysis was conducted. Model‐based ERN/CRN analysis showed an increased proportion of variance explained compared to the model‐free LMM, as indicated by the conditional *R* ^2^ statistic (from 0.043 to 0.068, ΔBIC = 178). Results revealed a significant main effect of Δ*Q*, *t*(29.13) = 2.22, *p* = 0.034, *b* = 0.63 (95% CI = 0.05 to 1.22), suggesting that ERN amplitudes increased with lower predictive accuracy (Δ*Q*). Thus, choosing a stimulus with lower subjective reward expectancy compared to the alternative stimulus predicted more pronounced ERN amplitudes, whereas choosing a stimulus with a larger subjective reward expectancy compared to the alternative stimulus predicted less pronounced ERN amplitudes. Importantly, there were no effects of learning status or group (*p*s ≥ 0.171).

## 3 Discussion

In the present study, we investigated performance monitoring in active and observational learning in OCD. Results showed widespread alterations in feedback learning and outcome processing in OCD patients compared to HCs, but only minor differences compared to SAD patients. Overall, we found that OCD patients showed equally impaired task performance when learning from their own versus observed actions and their outcomes. Notably, this was accompanied by abnormally positive FRN amplitudes following wins, especially during active learning.

### 3.1 Impaired Feedback Learning in OCD

Results on choice behavior were only partially in line with our hypotheses. As expected, task performance was impaired in OCD patients relative to HCs, reflected in reduced learning and lower overall choice accuracy. However, while learning impairments in OCD were observed in various experimental paradigms (e.g., Endrass et al. 2013; Luo et al. 2020; Nielen et al. 2009), these deficits were often rather nuanced. For instance, Endrass et al. (2013) found that OCD patients performing in a reversal learning task were impaired only after reversals. Moreover, learning in OCD was particularly impaired for negative feedback compared to positive feedback (Endrass et al. 2013; Nielen et al. 2009). However, in studies using probabilistic selection tasks similar to the one used in this study, previous studies did not indicate any impairments (Endrass et al. 2011; Nieuwenhuis et al. 2005). Notably, in the study by Endrass et al. (2011), participants did not collect points (or money); thus, increased motivational significance of feedback in our study may partly explain the diverging results.

Another key distinction is that the present study assessed both active and observational learning; therefore, it included test blocks with no external feedback. This required additional flexibility in recalling and maintaining knowledge about action–outcome associations gained during earlier learning blocks and may have added an additional layer of complexity and uncertainty. However, exploratory analysis of learning trial data similarly indicated impaired learning in OCD (for details see Supporting Information S1). Moreover, task performance in OCD patients was especially impaired when the reward contingency was high, as evidenced by the significant group‐by‐contingency interaction contrast. This may be explained by patients' unrealistic pessimism about obtaining rewards (Niemeyer et al. 2013; Zetsche et al. 2015), which may reduce the motivational impact of positive feedback, thereby particularly interfering with learning high‐contingency pairs.

Crucially, given the null effect of agency, our results expand previous findings by showing that OCD‐related deficits in feedback learning are evident across both active and observational learning contexts. This suggests that performance monitoring in OCD may not dissociate between active and observational learning. Although at least partially distinct neural systems have been discussed to underlie active and observational learning (Morelli et al. 2015), previous research has shown that overall learning performance is typically unaffected by agency in healthy individuals (Bellebaum and Colosio 2014; Bellebaum, Jokisch, et al. 2012; Peterburs et al. 2021; Rak et al. 2013). Comparable performance for active and observational learning in OCD therefore suggests neural systems supporting both forms of learning to be equally disrupted in OCD.

Impaired learning performance reflects only the surface level of aberrant choice behavior, and hence may be driven by distinct and potentially opposing maladaptive mechanisms. We hypothesized that deteriorated learning performance in OCD may arise from an increased tendency toward choice perseveration (Moritz et al. 2009). However, results on win‐stay/lose‐shift behavior demonstrated overall choice vacillation with overall *increased* shifting in OCD compared to HCs. What seems counterintuitive at first may relate to heightened intolerance of uncertainty in OCD (Hauser et al. 2017; Stern et al. 2013). Fradkin et al. (2020) recently proposed that both perseverative and shifting behavior can be observed in OCD but that they rely on the degree of perceived certainty in a given situational context. Thus, they predicted choice perseveration in well‐established and predictable situations, while under more ambiguous conditions with high uncertainty, OCD patients should rather tend to response shifting. In fact, Apergis‐Schoute et al. (2024) recently confirmed these predictions by showing more perseverative behavior in OCD patients than in controls in a deterministic reversal task, i.e., when outcomes were highly predictable. However, when the same patients were engaged in a probabilistic reversal task, thus inducing outcome uncertainty, OCD patients showed more indecisive behavior. This observation is also in line with various other studies reporting generally less perseveration but more choice shifting under uncertainty in OCD (e.g., Hauser et al. 2017; Kanen et al. 2019; Pushkarskaya et al. 2015). The probabilistic design in our study may likewise have induced uncertainty in OCD patients, thus promoting more indecisive, over‐exploratory behavior. This behavioral strategy closely resembles random exploration (e.g., Wilson et al. 2014), a low computational decision heuristic which may serve as a compensatory mechanism under uncertainty when inferring action–outcome relationships is hard to achieve. On the other hand, increased shifting may be a form of evidence gathering aimed at reducing uncertainty (Stern et al. 2013).

### 3.2 Altered Reward Processing in OCD

FRN amplitudes were generally attenuated in OCD patients relative to HCs, aligning with evidence suggesting a functional dissociation between error and feedback monitoring (see Endrass and Ullsperger 2014). Still, FRN studies in OCD are sparse and reveal rather inconsistent results, which may be at least partly due to differences in how the FRN was quantified. For instance, studies reporting FRN enhancement in OCD often rely on the FRN difference signal (loss‐win), therefore isolating valence coding (e.g., Luo et al. 2020; also see Bellato et al. 2021). Importantly, enhanced valence coding can in theory result from an enhanced FRN for negative feedback, a more positive FRN for positive feedback, or both. Therefore, enhanced valence coding and (general) FRN reduction may not necessarily be mutually exclusive. Indeed, our results show that the FRN distinguishes more strongly between loss and win feedback during active (but not observational) learning in OCD patients compared to HCs, as reflected in the significant group‐by‐agency‐by‐feedback‐valence interaction contrast. To our surprise, this effect was selectively driven by abnormally positive FRN amplitudes for positive feedback, especially for active learning, suggesting that reward—rather than punishment processing—may be disrupted in OCD.

This finding sharply contrasts with the widely held notion of hyperactive error monitoring in OCD (e.g., Pitman 1987). Instead, this result aligns with increasing evidence suggesting aberrant functioning of the reward system in OCD (Alves‐Pinto et al. 2019; Ferreira et al. 2017; Figee et al. 2011; Kaufmann et al. 2013; Koch et al. 2018; Marsh et al. 2015). Reward processing has been shown to mainly rely on the striatum (e.g., Balleine et al. 2007; Delgado et al. 2000, 2003; O'Doherty et al. 2004), which is substantially implicated in OCD pathophysiology (Burguiere et al. 2015). For instance, ventral striatal hypoactivation in OCD has been shown during reward anticipation (Figee et al. 2011), whereas other studies reported decreased activation in various brain areas including the striatum also during reward receipt (Koch et al. 2018; Remijnse et al. 2006), though the exact role of the reward system in OCD is still being explored (Bragdon et al. 2023).

Reduced FRN amplitudes in OCD have mainly been interpreted in terms of aberrant outcome expectations (e.g., O'Toole et al. 2012). Specifically, the FRN has previously been explained in terms of an over‐optimistic bias, i.e., the tendency toward anticipating positive outcomes (Oliveira et al. 2007). This bias is thought to be reduced in OCD, such that patients rather overestimate the occurrence of negative events (Moritz and Pohl 2009; Zetsche et al. 2015), which therefore has been assumed to contribute to the FRN reduction in OCD (O'Toole et al. 2012). Generally reduced FRN amplitudes in OCD may therefore reflect uncertainty and pessimistic reward expectations during learning. Notably, results from exploratory model‐based FRN analysis suggest that PE‐encoding was unaffected in OCD. This contrasts with previous studies showing enhanced (Hauser et al. 2017; Murray et al. 2019) or attenuated (Suzuki et al. 2023) PE signaling in OCD. However, these studies were based on estimates of the *signed* PE only and hence were unable to fully orthogonalize effects of general expectancy violations (i.e., SPE) from common feedback valence effects. Thus, when disentangling SPE and feedback valence in this study, our results do not support altered sensitivity for unexpected events in OCD per se, but suggest that dysfunctional reward anticipation in OCD may rather reflect impaired learning. Specifically, with successful learning, reward expectations for correct choices increase, while PE signals monotonically decrease. However, when learning is impaired, PE signals do not (or only slowly) decrease. Accordingly, when being rewarded, persistently high positive PE signals may lead to FRN attenuation (see Eppinger et al. 2008). This provides a reasonable explanation for why OCD patients show generally more positive FRN amplitudes, particularly following positive feedback, while FRN amplitudes for negative feedback were less affected. On the other hand, due to impaired learning in OCD, positive outcomes may not only be perceived as more unexpected but were in fact experienced less frequently, possibly further contributing to the observed processing differences.

Importantly, when expectancy violations were held constant in model‐based FRN analyses, planned contrasts still showed enhanced valence coding in OCD when compared to HCs, due to more positive FRN amplitudes for positive feedback. However, model‐based analysis indicated that this effect was agency‐invariant. This suggests that besides altered reward anticipation, aberrant reward evaluation and integration may also contribute to differential sensitivity to positive feedback. Thus, besides hyperactive error monitoring (Pitman 1987), hypoactive reward processing in OCD appears to be a reasonable explanation for the phenotypic expression of the disease. Accordingly, a reward can be understood as an indicator that an action has been successfully completed, thus serving as a stop signal. Notably, OCD patients typically report feelings of incompleteness including not‐just‐right experiences (Coles et al. 2003, 2005), and difficulties in stopping behaviors (Hinds et al. 2012; Wahl et al. 2008). Szechtman and Woody (2004) suggested that these difficulties may be due to a reduced signal of satiety and goal attainment, such that OCD patients seek continuous reaffirmation (but also see Stern and Taylor 2014). Reduced FRN amplitudes may reflect such attenuated feedback evaluation.

However, it has to be noted that FRN amplitudes to positive feedback have been interpreted differently in the context of depression: Here, enhanced as opposed to reduced FRN amplitudes for positive feedback have been suggested to indicate reward hyposensitivity in depression (e.g., Nelson et al. 2016; Proudfit 2015). Following this interpretation, FRN attenuation for positive feedback would rather indicate reward hypersensitivity, i.e., an enhanced reward signal in OCD. Indeed, it has also been argued that OC symptoms such as repetitive behaviors and mental acts may actually arise from being excessively rewarding (see Bragdon et al. 2023). Still, enhanced reward signals can result in maladaptive behaviors when they cannot be properly translated into adaptive adjustment strategies, perhaps related to cognitive inflexibility. Alternatively, biased attentional processing of threat‐related stimuli (Moritz and Pohl 2009) possibly makes rewards more salient. This would also be supported by increased P3 amplitudes for positive feedback in OCD (see the Supplement). Either way, altered reward processing in OCD aligns well with our behavioral results, such as impaired learning when reward contingency is high or maladaptive choice shifting following (authentic) wins, suggesting dysfunctional integration of positive outcomes in OCD.

### 3.3 Processing of Vicarious Feedback in OCD

While FRN amplitudes were generally attenuated in OCD patients compared to HCs across both active and observational learning conditions, greater differentiation between positive and negative feedback in the FRN was observed in OCD patients only during active, but not observational learning. This finding aligns with prior evidence of striatal dysfunction in OCD (Burguiere et al. 2015) and the notion that active and observational learning rely on similar yet distinct neural mechanisms, particularly suggesting a minor role of the striatum for processing of vicarious feedback (Kobza and Bellebaum 2015; Kobza et al. 2012). However, note that even though the striatum may be less involved in observational learning, it does not appear to operate according to an all‐or‐nothing principle, but still contributes to the processing of observed outcomes (e.g., see Bellebaum, Jokisch, et al. 2012; Burke et al. 2010; Cooper et al. 2012).

Importantly, the FRN has been shown to reflect striatal (Becker et al. 2014; Carlson et al. 2011; Foti et al. 2011) and/or midcingulate processing (Hauser et al. 2014; Oerlemans et al. 2024). In line with decreased striatal involvement in observational learning, FRN studies typically report reduced FRN valence coding for observational versus active learning (Bellebaum and Colosio 2014; Bellebaum et al. 2010), which we could replicate in the present study. Moreover, the FRN has recently been shown to reflect a (signed) PE signal only in active but not observational learning (Burnside et al. 2019), consistent with our findings from exploratory model‐based FRN analysis.

Crucially, as OCD patients were similarly impaired in active and observational learning, greater differentiation of the FRN for win and loss feedback in active but not observational learning further suggests differential contributions of the striatum to the learning from self‐ versus other‐related feedback. Hence, the FRN appears to reflect impaired learning, i.e., attenuated choice valuation and thus persistently enhanced (positive) PEs only for active but not (or to a lesser extent) for observational learning. This suggests further reliance on other brain areas *not* captured by the FRN that may contribute to impaired learning also in observational contexts. For instance, activation of the ventromedial prefrontal cortex (vmPFC) has been reported similarly for both processing personal and observed performance feedback (Morelli et al. 2015) and has been linked to the computation of subjective value during feedback‐based learning (Bartra et al. 2013; Gläscher et al. 2009). However, unlike the striatum, the vmPFC tracks subjective values agency‐invariantly, i.e., similarly for both active and observational learning (Zaki et al. 2014). OCD has additionally been associated with vmPFC hyperactivation (e.g., Apergis‐Schoute et al. 2017; Fitzgerald et al. 2005; Stern et al. 2011; also see Robbins et al. 2019). Given its ubiquitous role in the computation of subjective value, alterations in vmPFC functioning in OCD may contribute to deficient action–outcome learning and encoding of expected reward both during active and observational learning. However, this assumption remains highly speculative, as the role of the vmPFC in value encoding and feedback learning could not be tested in this study. However, model‐based FRN analysis indicated that altered reward processing in OCD does not depend on agency when controlling for reward expectancy. Therefore, processing differences between active and observational learning in model‐free analyses may instead reflect (subtle) differences in learning performance and reward anticipation.

### 3.4 Normalized Action Monitoring for Decision‐Making Under Uncertainty in OCD

Although we did not find the ERN/CRN to be more negative for incorrect (ERN) compared to correct choices (CRN) post‐learning, the significant accuracy‐by‐learning‐stage interaction showed that the ERN/CRN distinction was stronger post‐learning, generally in line with our hypothesis. This finding is consistent with prior evidence showing learning‐related increases in ERN amplitude (e.g., Bellebaum and Colosio 2014; Gawlowska et al. 2018), thus supporting the notion that the ERN reflects predictive action evaluation (Holroyd and Coles 2002; Maurer et al. 2021; Walsh and Anderson 2012). Results from our model‐based ERN/CRN analysis provide further evidence for this idea by showing that lower individual predictive accuracy (Δ*Q*) successfully predicted higher ERN amplitudes, regardless of the current learning status.

Moreover, ERN amplitudes did not differ between OCD patients and HCs. Although this finding contradicts prior evidence showing overactive error monitoring in OCD, this result aligns with meta‐analytical evidence suggesting ERN enhancement in OCD only under conditions involving response conflict (Riesel 2019). Moreover, this suggests distinct underlying mechanisms when monitoring simple motor errors during response conflict and more complex decision‐making during feedback‐based learning (Gründler et al. 2009). Importantly, it has been suggested that action monitoring demands in OCD may decrease when external feedback is available by reducing anxiety at the time of action execution, as there is no need for internal response evaluation (Nieuwenhuis et al. 2005). Alternatively, the results may again be explained in terms of uncertainty in outcome prediction. Thus, when actions are unmistakably incorrect, such as in simple response time tasks, action monitoring may become hyperactive in OCD. However, when associations between actions and outcomes are more ambiguous, such as in probabilistic learning, increased uncertainty about the response outcome may mask otherwise overactive error monitoring, and hence normalize action monitoring. Thus, deficits in outcome prediction under uncertainty would interfere with the implementation of adaptive, goal‐directed behavior, and could therefore explain impaired task performance in OCD. However, note that compared to other tasks showing ERN enhancement in OCD, ERN amplitudes in the present task were generally rather small (< 2 μV). Accordingly, low amplitudes combined with the relatively high variance in the data may also have contributed to the lack of significant group effects in our data.

### 3.5 Role of Transdiagnostic Mechanisms

Comparisons between OCD and SAD patients revealed that performance monitoring alterations were generally similar in both disorders, suggesting shared, disorder‐general mechanisms. This is not surprising given the shared pathophysiology including abnormal cingulate (Brennan et al. 2015; Nutt et al. 1998; Phan et al. 2005) and striatal (Burguiere et al. 2015; Manning et al. 2015) functioning as well as the involvement of the dopaminergic and serotonergic neurotransmitter system in both disorders (Denys et al. 2004; Hesse et al. 2005; Stein et al. 2002).

Shared pathophysiology is also reflected at the phenomenological level by numerous overlapping features (Carpita et al. 2020). Our results suggest that deficient value assignment and uncertainty in outcome prediction may constitute potential transdiagnostic mechanisms (e.g., Cavanagh and Shackman 2015; but also see Rosser 2019). Moreover, abnormal predictive processing under uncertainty in clinical anxiety has been implicated in an integrative neurobiological model, relying on the MCC (Grupe and Nitschke 2013). Accordingly, we argue that specifically outcome uncertainty may have hindered learning and may therefore account for similar performance monitoring alterations in OCD and SAD in the present study. In support of this assumption, intolerance of uncertainty has not only been linked to OCD but also to SAD (Boelen and Reijntjes 2009; Carleton et al. 2010). Beyond that, both OCD and SAD patients have been linked to biased attentional processing of threat‐related stimuli (Mathews and Mackintosh 1998; Moritz and Pohl 2009). Supplementary analyses examining modulation by depressive symptoms showed that impaired task performance and indecisiveness, as well as altered feedback processing—specifically, disrupted PE coding in the FRN during active learning—were associated with higher BDI‐II scores (for details, see Supplement). Note, however, that OCI‐R and BDI‐II scores were moderately positively correlated, suggesting that the symptom scores used in this study may have been influenced by shared, transdiagnostic mechanisms, making it difficult to clearly disentangle OC‐ and depression‐related contributions.

Despite extensive similarities in performance monitoring between OCD and SAD, some minor OCD‐specific effects were observed. This particularly concerns choice behavior and adaptations to feedback. However, these effects likely represent rather nuanced expressions of the same transdiagnostic mechanisms. Interestingly, controlling for expectancy in model‐based FRN analyses showed valence coding in OCD not only to be enhanced relative to HCs but also to SAD patients, suggesting early processing differences in stimulus valuation. Processing differences between OCD and SAD were also found for supplementary model‐free P3 analysis, though group differences were absent in our exploratory model‐based P3 analysis, therefore suggesting that this effect was entirely driven by expectancy (see Supplement). Collectively, however, while there may be processing differences between OCD and SAD, these effects appear to be rather subtle and not action‐relevant. Still, OC symptoms also modulated performance monitoring, both at the behavioral and neural level. However, there were no clear relationships that could fully account for the OCD group effects reported for the categorical analyses, again highlighting the role of transdiagnostic mechanisms.

### 3.6 Strength, Limitations and Future Directions

We observed widespread alterations in feedback monitoring in OCD patients compared to neurotypical controls. This evidence stems from comprehensive data analyses using single‐trial (G)LMMs, which have been proposed to be the preferred analysis method compared to traditional averaging approaches (such as ANOVAs; Quené and Van den Bergh 2004; Volpert‐Esmond et al. 2018, 2021). A major strength of the present study was the recruitment of a transdiagnostic sample, including OCD and SAD patients, which allowed us to additionally examine disorder‐general or ‐specific mechanisms that may contribute to the psychopathology of OCD.

Nevertheless, several limitations must be acknowledged. First, patients with comorbid disorders were included in this study. While this may not compromise the generalizability and external validity of our results, it introduces the possibility that the observed effects were neither driven solely by OCD nor SAD psychopathology (including shared pathophysiological mechanisms). Due to high rates of comorbidity in both patient groups, we were not able to further explore disorder‐unique contributions to feedback monitoring. Anxiety has also been shown to account for variations in (overactive) error processing (Meyer 2016), and may therefore explain alterations in feedback processing. Unfortunately, we did not collect estimates of trait anxiety across the whole sample. Moreover, most patients were on medication at the time of testing. Dopamine and serotonin have been implicated in the etiology of both OCD (Denys et al. 2004; Hesse et al. 2005; Pauls et al. 2014) and SAD (Nutt et al. 1998; Stein et al. 2002), and have been thought to substantially contribute to performance monitoring (Jocham and Ullsperger 2009). Crucially, Murray et al. (2019) showed that increased cingulate PE signaling was reduced under acute dopaminergic drug therapy using both the DA‐receptor agonist pramipexole and DA‐receptor antagonist amisulpride, while no modulatory effect on choice behavior was observed. On the other hand, the ERN in OCD has been shown to be insensitive to medication (Riesel 2019), suggesting that at least certain neurocognitive markers may be unaffected by medication use. However, potential modulatory effects of psychotropic drug intake can only be ruled out when testing medication‐free, ideally drug‐naïve patient populations. Again, as most of our patients were receiving medication, we were not able to conduct such subgroup analyses.

Despite these limitations, our results may have important implications. More research is needed on feedback and especially reward processing in OCD. Impaired value computation and reward expectation in OCD, as suggested by our data, need further validation and should be explicitly addressed in future work by using dedicated task designs which allow PE modeling for both active and observational learning. Moreover, our results strongly highlight the importance of extending research on performance monitoring to observational learning, especially in clinical populations (see Musco et al. 2023). Due to widespread impairments in social functioning in many mental disorders, it has been recently proposed to re‐conceptualize them as disorders of social interaction (Schilbach 2016; Schilbach et al. 2013). This view allows for a further breakdown of relevant (sub‐)processes of social interactions that may be impaired in mental disorders. Along these lines, future research in clinical populations should not be limited to (passive) observation but should be extended to more fine‐grained social interactions such as cooperation or competition. Last, our study highlights the importance of transdiagnostic research, encouraging direct comparison of more patient groups and/or dimensional approaches to further unveil disorder‐specific or ‐general mechanisms underlying psychopathology.

## 4 Conclusion

The present results suggest deficient feedback learning in OCD in active *and* observational learning, as reflected in overall impaired task performance and indecisive decision‐making compared to neurotypical individuals. At the neural level, impaired learning in OCD patients was reflected in abnormally reduced, i.e., more positive FRN amplitudes, most particularly for positive feedback during active learning, with enhanced valence coding for active but not observational learning. Conversely, there were no significant group differences in action monitoring reflected in the ERN/CRN. Similar results for SAD patients portend reliance on transdiagnostic mechanisms. Overall, our results suggest altered reward processing—not punishment processing—in OCD. As this conflicts with the prevailing notion of overactive error monitoring in OCD, future research is needed to clarify the significance of our results.

## Author Contributions

**Julian Vahedi:** investigation, methodology, formal analysis, data curation, visualization, writing – original draft, writing – review and editing. **Armin Bahic:** resources, writing – review and editing. **Irini Chaliani:** resources, writing – review and editing. **Leonhard Schilbach:** resources, writing – review and editing. **Burkhard Ciupka‐Schön:** resources, writing – review and editing. **Christian Bellebaum:** project administration, conceptualization, methodology, resources, supervision, writing – review and editing. **Reinhard Pietrowsky:** funding acquisition, project administration, conceptualization, methodology, resources, supervision, writing – review and editing. **Jutta Peterburs:** funding acquisition, project administration, conceptualization, methodology, supervision, writing – review and editing.

## Disclosure

AI‐assisted writing tools were occasionally used during internal revision of the original draft to proof the document for language errors and improve readability. AI‐generated rewording was carefully reviewed and adopted only if it contributed significantly to a better understanding of the content.

## Ethics Statement

All procedures performed in this study were in accordance with the ethical standards of the institutional and/or national research committee and with the 1964 Helsinki declaration and its later amendments or comparable ethical standards.

## Consent

Informed consent was obtained from all individual participants included in the present study.

## Conflicts of Interest

The authors declare no conflicts of interest.

## Supporting information


Data S1.

## Data Availability

Data and code to reproduce all analyses in this manuscript can be found on the Open Science Framework (https://osf.io/a6uzm/?view_only=aeaca475dcfb4465bff8c9f4071dd350).
