# Supplementary material for: Altered Reward Processing in Obsessive–Compulsive Disorder: Insights From Active and Observational Learning
Source: Psychophysiology. 2025 Sep 7;62(9):e70142. doi: 10.1111/psyp.70142 (PMC12415511; doi:10.1111/psyp.70142)
Supplement: Supplementary file 1 — Data S1. [file PSYP-62-e70142-s001.docx]

**Supplement to
Altered Reward Processing in Obsessive-Compulsive Disorder: Insights from Active and Observational Learning**

Julian Vahedi^1*^, Armin Bahic^2^, Irini Chaliani^2^, Leonhard Schilbach^2,3^, Burkhard Ciupka-Schön^1^, Christian Bellebaum^1^, Reinhard Pietrowsky^1^ & Jutta Peterburs^4^

^1^Heinrich Heine University Düsseldorf, Faculty of Mathematics and Natural Sciences, Düsseldorf, Germany

^2^Clinic for Psychiatry and Psychotherapy/LVR-Clinic Düsseldorf, Medical Faculty and University Hospital Düsseldorf, Heinrich Heine University Düsseldorf, Düsseldorf, Germany

^3^Ludwig-Maximilians-Universität München, Medical Faculty, München, Germany

^4^MSH Medical School Hamburg, Institute for Systems Medicine and Department of Human Medicine, Hamburg, Germany

*Corresponding author: Julian Vahedi ([julian.vahedi@hhu.de](mailto:julian.vahedi@hhu.de)), Heinrich Heine University Düsseldorf, Institute for Experimental Psychology, Department of Biological Psychology Universitätsstr. 1, D-40225 Düsseldorf, Germany

**Author contribution statement (CRediT):**

Julian Vahedi: Investigation, methodology; formal analysis; data curation; visualization; writing-original draft (lead); writing – review and editing. Armin Bahic: Resources; writing - review and editing. Irini Chaliani: Resources; writing - review and editing. Leonhard Schilbach: Resources; writing - review and editing. Burkhard Ciupka-Schön: Resources; writing - review and editing. Christian Bellebaum: Project administration; conceptualization; methodology; resources; supervision; writing – review and editing. Reinhard Pietrowsky: Funding acquisition; project administration; conceptualization; methodology; resources; supervision; writing – review and editing. Jutta Peterburs: Funding acquisition; project administration; conceptualization; methodology; supervision; writing – original draft (supporting); writing – review and editing.

**Conflict of interest statement:**

The authors declare no conflicts of interest.

**Table of contents**

[Supplementary methods 5](#_Toc204617633)

[Case exclusions 5](#_Toc204617634)

[Transfer phase details 5](#_Toc204617635)

[Task performance based on learning trials in active learning 5](#_Toc204617636)

[Propensity to seek reward/avoid punishment 6](#_Toc204617637)

[P3 analysis 7](#_Toc204617638)

[Brain-behavior relationships 8](#_Toc204617639)

[Computational modelling of behavioral data 8](#_Toc204617640)

[Modelling of test/transfer trial behavior 10](#_Toc204617641)

[Model fitting and comparison 11](#_Toc204617642)

[Model validation 12](#_Toc204617643)

[Supplementary results 13](#_Toc204617644)

[Self-report questionnaires 13](#_Toc204617645)

[Task performance based on learning trials in active learning 13](#_Toc204617646)

[Learning propensity based on transfer phase performance 14](#_Toc204617647)

[Model-free P3 analysis 15](#_Toc204617648)

[Brain-behavior relationships 16](#_Toc204617649)

[Computational modelling results 17](#_Toc204617650)

[Model comparison 17](#_Toc204617651)

[Model validation 17](#_Toc204617652)

[Parameter estimates 18](#_Toc204617653)

[Model-based P3 analysis 18](#_Toc204617654)

[Relationship with depressive symptoms 19](#_Toc204617655)

[Choice behavior 19](#_Toc204617656)

[EEG 20](#_Toc204617657)

[References 22](#_Toc204617658)

[Table S1: Comorbid diagnoses 24](#_Toc204617659)

[Table S2: Medication 25](#_Toc204617660)

[Table S3: Artifact rejection during EEG preprocessing 26](#_Toc204617661)

[Table S4a: GLMM results on choice accuracy (test phase) 27](#_Toc204617662)

[Table S4b: GLMM results on choice accuracy (learning phase in active learning) 31](#_Toc204617663)

[Table S5: GLMM results on win-stay/lose-shift behavior 34](#_Toc204617664)

[Table S6: GLMM results on learning propensity 39](#_Toc204617665)

[Table S7: LMM results for the FRN (model-free) 41](#_Toc204617666)

[Table S8: LMM results for the ERN/CRN (model-free) 44](#_Toc204617667)

[Table S9: LMM results for the FRN (model-based) 46](#_Toc204617668)

[Table S10: LMM results for the ERN/CRN (model-based) 51](#_Toc204617669)

[Table S11: LMM results for the P3 (model-free) 53](#_Toc204617670)

[Table S12: LMM results for the P3 (model-based) 56](#_Toc204617671)

[Table S13: GLMM results on brain-behavior relationships 60](#_Toc204617672)

[Table S14a: Supplementary BDI-II analyses (choice accuracy during test trials) 64](#_Toc204617673)

[Table S14b: Supplementary BDI-II analyses (choice accuracy during learning trials) 66](#_Toc204617674)

[Table S15: Supplementary BDI-II analyses (win-stay/lose-shift behavior) 67](#_Toc204617675)

[Table S16: Supplementary BDI-II analyses (model-free FRN analysis) 69](#_Toc204617676)

[Table S17: Supplementary BDI-II analyses (model-free P3 analysis) 70](#_Toc204617677)

[Table S18: Supplementary BDI-II analyses (model-based FRN analysis) 72](#_Toc204617678)

[Table S19: Supplementary BDI-II analyses (model-based P3 analysis) 74](#_Toc204617679)

[Figure S1: Task performance across blocks 76](#_Toc204617680)

[Figure S2a: Active reinforcement learning model 77](#_Toc204617681)

[Figure S2b: Active reinforcement learning model 78](#_Toc204617682)

[Figure S3: Observational reinforcement learning model 79](#_Toc204617683)

# Supplementary methods

## Case exclusions

One patient with obsessive-compulsive disorder (OCD) was excluded due to self-reported history of psychotic symptoms. Moreover, three participants dropped out after the first experimental session due to low task motivation (2 healthy controls [HCs], 1 patient with social anxiety disorder [SAD]), and one OCD patient was excluded because of difficulties in understanding the experimental task. Additionally, two participants (1 HC, 1 OCD) as well as electrophysiological data for observational learning of three participants (2 HC, 1 OCD) had to be excluded due to technical problems during data recording. Furthermore, two SAD patients had to be excluded from electrophysiological data analyses due to poor EEG data quality (excessive noise, >25% of segments discarded during artifact rejection).

## Transfer phase details

At the end of both the active and observational task variant, participants completed a 40-trial transfer phase. In the transfer phase, stimulus A and stimulus B were paired with all other stimuli to assess the propensity to seek reward (by preferring A over the alternative option in A/C, A/D, A/E, A/F), and to avoid punishment (by deciding against B in B/C, B/D, B/E, B/F). Like test trials, also transfer trials required active responding by both active and observational learners. Again, choices were not followed by feedback, but participants were instructed to keep choosing stimuli they thought would most likely maximize reward.

## Task performance based on learning trials in active learning

To allow for a better comparison with previous studies from active learning in OCD, we re-ran generalized linear mixed-effects model (GLMM) analysis on choice accuracy for active learning trial data. Fixed-effect predictors included the categorical between-subjects factor group (*OCD [=reference], HC, SAD*) as well as the continuous within-subjects factors contingency and block (both centered around 0), written in Wilkinson notation as:

$$Choice Accuracy \sim Group* Contingency*Block+$$

$$(1+Contingency*Block|Participant)$$

## Propensity to seek reward/avoid punishment

To assess the participants’ propensity to learn from positive and negative feedback, they completed a transfer phase at the end of the task (see e.g., Bellebaum et al., 2012; Frank et al., 2004). Learning propensity was assessed in reference to the easiest learnable stimuli, i.e., those with the highest (A=80%) and lowest (B=20%) reward probability. Hence, learning to prefer stimulus A over stimulus B may arise from learning that A is favorable, thus leading to reward (=positive learning, i.e., seek reward when choosing A) or learning that B is unfavorable, thus associated with punishment (=negative learning, i.e., avoid punishment when deciding against B).

Accordingly, to investigate propensity to learn to seek reward or avoid punishment, GLMM analysis on single-trial choice accuracy data (0=incorrect, 1=correct) was conducted for new stimulus pairings introduced in the transfer phase. As with other analyses using choice accuracy as the dependent variable, choice accuracy reflected selecting the stimulus with the higher reward probability (i.e., select stimulus A, avoid stimulus B). Moreover, note that this analysis was restricted to participants classified as learners (>65% for stimulus pair A in the last test block before entering the transfer phase, as determined by a one-tailed binomial test, α = .05). In order to minimize data loss, participants were classified as (non-)learners separately for active and observational learning. Accordingly, 27 OCD patients (26 active, 22 observational), 29 SAD patients (27 active, 27 observational), and 27 HCs (27 active, 23 observational) exceeded the learning criterion and were included in the GLMM analysis. Categorical fixed-effect predictors included the between-subjects factor group (*OCD [=reference], HC, SAD*) and the within-subjects factors agency (*active* *[=reference], observational*) and trial type (*choose A* *[=reference], avoid B*). The GLMM formula written in Wilkinson notation was:

$$Choice Accuracy \sim Group*Agency* Trial Type+$$

$$(1+Agency*Trial Type|Participant)$$

## P3 analysis

Analysis of feedback-locked P3 amplitudes followed the same procedure as that described for the FRN in the main manuscript. Single-trial P3 amplitudes were scored based on a hierarchical data-driven approach in a predefined centro-parietal cluster (Cz, CP1, CP2, Pz). To this end, P3 peaks were searched separately in composite P3 waveforms for active and observational learning data in an a priori search window 290-450 ms after feedback presentation (van Dinteren et al., 2014). The P3 was then scored as the mean amplitude within 321-381 ms for active learning, and 327-387 ms for observational learning. Linear mixed-effects model (LMM) specification was identical to the that reported for the FRN. Thus, the model-free P3 LMM was written as the following Wilkinson notation formula:

$${P3}_{amp} \sim Group*Agency* Feedback Valence+\left( 1+Agency*Feedback Valence \right| Participant)+(1|Electrode)$$

For the model-based P3 LMM analysis, this model was extended by including the salience prediction error (SPE, for details on the computational modelling of the behavioral data, see the respective section below), specified according to the following Wilkinson notation formula:

$${P3}_{amp} \sim Group*Agency* Feedback Valence*SPE+\left( 1+Agency*Feedback Valence*SPE \right| Participant)+(1|Electrode)$$

## Brain-behavior relationships

In an exploratory analysis, we investigated whether trial-level FRN and P3 amplitudes predicted subsequent choice behavior in active learning. Specifically, we were interested whether choice shifting varied as a function of the previous trial’s FRN or P3 amplitude. For this purpose, we performed a GLMM analysis, similar to that reported for behavioral win-stay/lose-shift behavior in the main manuscript. Importantly, here we were primarily interested in whether FRN /P3 amplitudes predicted lose-shift behavior, therefore restricting the analysis to post-punishment shifting, i.e., trials following negative feedback. In order to test brain-behavior relationships, we included FRN/P3 amplitudes as additional continuous predictors in separate GLMMs. As for those analyses we were primarily interested in the effect of within- but not between-participant variation in the FRN/P3 on behavioral change, FRN and P3 amplitudes were centered to the individual, subject-level mean and scaled (divided by 10) prior to model fitting (LoTemplio et al., 2023; also see Enders & Tofighi, 2007). The model formulae (with the maximal-possible random-effects structure) written in Wilkinson notation were specified as follows:

$${M1}_{BBR}: Choice shift \sim Group*Previous Feedback Authenticity*Block*Previous FRN+ (1+Previous Authenticity*Block|Participant)$$

$${M2}_{BBR}: Choice shift \sim Group*Previous Feedback Authenticity*Block*Previous P3+ (1+Previous Authenticity*Block+Previous P3|Participant)$$

## Computational modelling of behavioral data

We fitted different reinforcement learning (RL) models to each participants’ choice sequence and reinforcement history (Sutton & Barto, 2018). All models incorporated a Q-value $Q_{c}\left( t \right)$ for each given choice *c* in trial *t* as an estimate of the expected sum of future reward, thus reflecting the reward expectation, when choosing *c* (Sutton & Barto, 2018; Watkins & Dayan, 1992). Q-values ranged from 0 to 1, with values close to zero reflecting low and values close to 1 reflecting high reward expectation for a given choice. Q-values were initially set to 0.5 $(Q_{0})$ for each stimulus, assuming that participants were neither expecting to win nor lose when first encountering a new stimulus pair. In feedback trials, Q-values were updated based on the prediction error $\delta$ (PE), calculated as the difference between the reward expectation for the current choice and the actually received outcome ($r$) on a given trial

$$\delta(t)= r(t)-Q_{c}\left( t \right)$$

where $r\in\{$0,1}, with losses coded as 0s and wins coded as 1s. Therefore, $\delta$ > 0 represents a positive PE, i.e., outcomes that are better than expected, and $\delta$ < 0 represents a negative PE, i.e., outcomes that are worse than expected. Q-values for the chosen option $c$ were updated according to an extended Q-learning algorithm with dual learning rates $\alpha^{+/-}$(0 ≤ $\alpha^{+/-}$≤ 1):

$$Q_{c}\left( t+1 \right)= \left\{ \begin{aligned} Q_{c}\left( t \right)+ \alpha^{+}\delta(t) if \delta(t)>0 \\ Q_{c}\left( t \right)+ \alpha^{-} \delta(t) if \delta(t)<0 \end{aligned} \right.$$

Therefore, $\alpha^{+}$ reflects increased sensitivity to recent positive, and thus confirmatory feedback, and $\alpha^{-}$ suggests increased sensitivity to recent negative, and thus disconfirmatory feedback (see e.g., Burnside et al., 2019; Frank et al., 2007; Palminteri et al., 2017). Importantly, implementing separate learning rates for positive and negative feedback allowed us to explicitly test the presence of optimistic belief updating (i.e., $\alpha^{+}$ > $\alpha^{-}$), which has been associated with the unrealistic optimism bias (Lefebvre et al., 2017; Sharot & Garrett, 2016), thought to be altered in OCD (Moritz & Pohl, 2009; Zetsche et al., 2015).

### Modelling of test/transfer trial behavior

While the choice sequence during learning trials reflected participants' own decision-making in active learning, in observational learning they observed another participant's choices, which may therefore not necessarily reflect how their own reward expectancies would have been translated into stimulus selection. Thus, we sought to include test and transfer trials in the modelling of choice behavior (also see e.g., Dercon et al., 2024; Kurtenbach et al., 2022; Ptasczynski et al., 2022), which, although not including trial-level feedback, required active responding in both active and observational learning.

Building on recent findings suggesting that reward expectations may update even when no external feedback is provided (Ptasczynski et al., 2022), we fitted and compared different RL models based on three alternative hypotheses about how reward expectancies might evolve in the absence of feedback:

First, we assumed that reward expectancies may not update, and be fixed to the Q-value of the last feedback trial (see Dercon et al., 2024; Ptasczynski et al., 2022). Accordingly, for M_Static_, Q-values remained static throughout test and transfer trials, i.e.,

$$Q_{c}\left( t+1 \right)= Q_{c}\left( t \right)$$

Second, when explicit trial-wise feedback is omitted, decision-making may alternatively be prone to decay or devaluation. Thus, in M_Dev_, Q-values were allowed to decay toward the initial action value $(Q_{0})$ based on the devaluation learning rate parameter $\varphi$ (0 ≤ $\varphi$ ≤ 1):

$$Q_{c}\left( t+1 \right)=Q_{c}\left( t \right)+{\varphi(Q}_{0}-Q_{c}\left( t \right))$$

where values of $\varphi$close to 1 indicate large devaluation speed and values close to 0 indicate no/little devaluation of reward expectations (Collins, 2018).

Finally, motivated by findings on choice-induced preference change (CIPC) when there is no external feedback (Luettgau et al., 2020; Zhu et al., 2021; Zhu et al., 2024), we assumed that Q-values may increase, even without explicit feedback information. Note, that such choice-induced value updating has previously also been considered to reduce cognitive dissonance (Festinger, 1957) between the chosen and unchosen stimulus (also see Colosio et al., 2017; Ptasczynski et al., 2022). Therefore, M_CIPC_ used a CIPC parameter $\varepsilon$ (0 ≤ $\varepsilon$ ≤ 1) to update Q-values of both the chosen option $c$ while devaluating the unchosen option $\bar{c}$, as suggested by Zhu et al. (2021):

$$Q_{c}\left( t+1 \right)=Q_{c}\left( t \right) +\varepsilon(1-Q_{c}\left( t \right))$$

$$Q_{\bar{c}}\left( t+1 \right)=Q_{\bar{c}}\left( t \right)-{\varepsilon Q}_{\bar{c}}\left( t \right)$$

Here, values of $\varepsilon$ close to zero indicate slow and values close to 1 indicate high updating speed of the (un)chosen option.

### Model fitting and comparison

To obtain unique parameter estimates for active and observational learning, models were fitted separately to active and observational learning data. From then on, model fitting was completely identical such that Q-values were updated in learning trials based on the (observed) actions and outcomes. Crucially, these Q-values were then used to fit the model to predict choice behavior in the test and transfer phases (for a similar approach to modeling observational learning data see Schultner et al., 2024). Thus, Q-values were converted into choice probabilities $p$, i.e., the likelihood of choosing one symbol over another using a softmax function with an inverse temperature parameter $\beta$ (0 ≤ $\beta$ ≤ 50) representing the degree to which the stimulus value determines stimulus selection.

$$p_{c}\left( t \right)= \frac{\exp(Q_{c}\left( t \right) \beta)}{\sum_{c'} \exp\left( Q_{c'}\left( t \right) \beta\right)}$$

Higher values of $\beta$ indicate more deterministic choices, while lower values indicate more explorative decision-making.

For a comparable assessment of model parameters and latent reward expectations in both active and observational learning, model parameters were estimated by minimizing the negative log likelihood (NLL) based on the predicted choice probabilities for test and transfer trials.

$$NLL= -\sum_{t} log(p_{c}(t))$$

For each model and participant, we repeatedly started optimization from 100 random starting points to minimize the risk of convergence to local optima. Models were finally compared to determine the winning model that best predicted the actual test/transfer phase behavior based on the Bayesian information criterion (BIC):

$$BIC=2NLL+ n_{params} log(N)$$

where $n_{params}$ is the numbers of free parameters for a given model and $N$ the number of observations and lower BIC scores indicate a better model fit.

### Model validation

To validate the winning models, we assessed parameter identifiability through inter-correlations and conducted parameter recovery using simulated data, following Palminteri et al. (2017). Inter-correlations were calculated using Pearson’s correlation coefficient, where low correlations (|r| ≤ 0.50) indicate good parameter identifiability. For parameter recovery, we re-fitted the winning models to 25 synthetic datasets per participant, simulated using the parameter estimates derived from the true experimental data (Wilson & Collins, 2019). Parameter recovery was assessed by calculating Pearson’s correlations between the true (empirical) and recovered (synthetic) parameter estimates, with strong correlations (r ≥ 0.70) indicating successful recovery. To reduce noise, recovered parameters were averaged across simulations for each participant.

# Supplementary results

## Self-report questionnaires

Age, verbal intelligence (MWT-B), and clinical scores (BDI-II, OCI-R, OBQ-D) were compared between diagnostic groups using (univariate) analyses of variance (ANOVAs; Scheffe, 1999; also see Schmider et al., 2010) where separate ANOVAs were run for each self-report measure set as the dependent variable, and diagnostic group as the independent between-subjects factor. Post-hoc *t*-tests with Bonferroni adjustment (Hochberg, 1987) were used to resolve significant main effects of diagnostic group.

There were no group differences regarding age, *F*(2, 80) = 0.16, *p* = .856, and verbal intelligence, *F*(2, 80) = 0.54, *p* = .587. However, significant group differences emerged for the BDI-II, *F*(2, 80) = 23.47, *p* < .001, with higher BDI-II scores in OCD, *t*(80) = -6.10, *p_adj_* < .001, and SAD, *t*(80) = -5.78, *p_adj_* < .001, compared to HC. Moreover, there were significant group differences regarding the OCI-R, *F*(2, 80) = 26.71, *p* < .001, with larger OCI-R scores in OCD compared to both HC, *t*(80) = -7.05, *p_adj_* < .001, and SAD, *t*(80) = 5.24, *p_adj_* < .001. Last, group differences were found for the OBQ-D, *F*(2, 80) = 12.99, *p* < .001, where higher scores were found in OCD relative to HCs, *t*(80) = -5.02, *p_adj_* < .001, and in SAD relative to HCs, *t*(80) = -3.31, *p_adj_* = .004.

## Task performance based on learning trials in active learning

GLMM analysis results for the active learning trial data largely aligned with those for the test trial data. Deviating from the results on test trials, there was a significant block × contingency interaction effect, *z* = -2.94, *p* = .003, *b* = -0.14 (95% CI = -0.23 – -0.05). Resolving this interaction showed that the strength of the contingency effect varied with task progression. Specifically, post-hoc comparisons of the contingency slope estimates between learning blocks, revealed that the effect of contingency was increasingly stronger for later blocks (*p_adj_* ≤ .003), hence indicating successful learning to dissociate high and low contingency conditions across blocks.

Group differences emerged by planned contrasts for the group × block interaction effect showing impaired learning across blocks in OCD compared to HCs, *z* = 2.02, *p* = .043, *b* = 0.24 (95% CI = 0.01 – 0.47), but no differences between OCD and SAD (*p* = .702). Moreover, planned contrasts for the group × contingency interaction effect revealed a larger contingency effect in HCs relative to OCD patients, *z* = -1.91, *p* = .049, *b* = --0.39 (95% CI = --0.78 – -0.00). Further elucidation showed that performance differences were most pronounced for the high contingency pair (A/B), *z* = 2.35, *p_adj_* = .057, *b* = 0.88 (95% CI = 0.15 – 1.63), and decreased for the medium (C/D), *z* = 1.90, *p_adj_* = .068, *b* = 0.50 (95% CI = -0.02 – 1.01), and low (E/F) contingency pair, *z* = 0.40, *p_adj_* = .688, *b* = 0.11 (95% CI = -0.42 – 0.63), although *p*-values were all non-significant after applying FDR-correction. Transdiagnostic-dimensional GLMM analysis did not yield a significant effect involving the predictor OCI-R (*p*s ≥.089) suggesting that the effects described above may not be OCD-specific (also see [Table S4b](#_Table_S4b:_GLMM)).

## Learning propensity based on transfer phase performance

The propensity to seek reward and/or avoid punishment was assessed for participants classified as learners (for details, see above). GLMM analysis yielded a significant main effect of trial type, *z* = -2.59, *p* = .010, *b* = -0.37 (95% CI = -0.64 – -0.09), indicating overall better performance in seeking reward (i.e., choosing A) than avoiding punishment (i.e., avoiding B). All other effects failed to reach statistical significance (all *p*s ≥ .082, but see [Table S6](#_Table_S6:_GLMM) for detailed GLMM results).

## Model-free P3 analysis

LMM analysis for the centro-parietal P3 revealed a significant main effect of agency, *t*(77.22) = -11.99, *p* < .001, *b* = -6.74 (95% CI = -7.86 – -5.62), indicating overall increased, i.e., more positive P3 amplitudes in active compared to observational learning. Agency was further qualified by significant agency × feedback valence interaction effect, suggesting that the P3 difference for loss versus win feedback was larger for active compared to observational learning, *t*(78.61) = -2.12, *p* = .037, *b* = -0.75 (95% CI = -1.44 – -0.05).

Planned a priori contrasts revealed marginally larger P3 amplitudes in OCD patients compared to controls, *t*(78.25) = -2.00, *p* = .049, *b* = -2.72 (95% CI = -5.42 – -0.01). Moreover, planned interaction contrasts showed that the agency × feedback valence interaction effect reflecting enhanced valence coding (loss-win) for active versus observational learning was larger in HCs compared with OCD patients, *t*(79.19) = -2.34, *p* = .022, *b* = -2.02 (95% CI = -3.74 – -0.30), as well as in SAD compared to OCD patients, *t*(77.90) = -2.61, *p* = .039, *b* = -1.80 (95% CI = -3.51 – -0.09). Note, that attenuated P3 valence coding can theoretically be driven by enlarged P3 amplitudes for positive feedback, reduced P3 amplitudes for negative feedback, or by both. Although attempts to unravel this underlying mechanism by controlling for multiple comparisons did not reveal any significant effects (*p*s*_adj_* ≥ .057), uncorrected comparisons suggested a subtle P3 increase for win (*p*_uncorr._ = .029) but not loss feedback (*p*_uncorr._ = .195) in OCD compared to HCs.

Transdiagnostic-dimensional analysis yielded a significant OCI-R × agency × feedback valence interaction effect, *t*(81.55) = 2.32, *p* = .023, *b* = 0.06 (95% CI = 0.01 – 0.11). Although OCI-R scores did not predict P3 amplitude in either valence × agency combination (*p*s*_adj_* ≥ .467), spotlight analysis for high versus low OCI-R participants suggested that the agency × feedback valence interaction effect described above was only evident in participants scoring low on the OCI-R, *t*(80.25) = 3.14, *p_adj_* = .005, *b* = 1.70 (95% CI = 0.62 – 2.78). Detailed LMM output is provided in [Table S11](#_Table_S10:_LMM).

## Brain-behavior relationships

In a first GLMM including previous FRN amplitudes as a predictor of choice switching, results yielded a significant main effect of previous FRN, *z* = 2.50, *p* = .013, *b* = 0.07 (95% CI = 0.01 – 0.12), where generally reduced FRN amplitudes predicted higher probability to shift the choice policy. Beyond that, there were significant main effects of block, *z* = -2.80, *p* = .005, *b* = -0.10 (95% CI = -0.16 – -0.03), and previous feedback authenticity, *z* = -10.29, *p* < .001, *b* = -1.26 (95% CI = -1.50 – -1.02), as well as their interaction, *z* = -2.72, *p* = .006, *b* = -0.20 (95% CI = -0.34 – -0.05). Resolving this effect revealed that, while participants showed an overall increased probability of shifting their previous choice after authentic (versus misleading) losses, this tendency remained stable across blocks (p = .971). In contrast, after misleading losses, participants learned that the feedback was non-informative, leading to a gradual decrease in shifting probability across blocks, *z* = -4.09, *p* < .001, *b* = -0.19 (95% CI = - -0.29 – -0.10). Last, planned contrasts indicated generally more switching in OCD patients compared to HC, *z* = -1.97, *p* = .049, *b* = -0.26 (95% CI = -0.52 – -0.00), but no differences between OCD and SAD patients (*p* = .151). The second GLMM including previous P3 amplitude as a predictor mirrored these results, although here planned a priori comparisons between OCD patients and HCs as well as between OCD SAD patients did not reach statistical significance (*p*s ≥ .052). Here, the significant main effect of previous P3 indicated that larger P3 amplitudes predicted higher probability to shift, *z* = 2.35, *p* = .019, *b* = 0.06 (95% CI = 0.01 – 0.10).

Comparing the proportion of variance explained, as assessed by the (conditional) *R^2^* statistic, suggested that the FRN model accounted for a marginally greater proportion of variance in choice shifting (FRN: conditional *R^2^* = .226; P3: conditional *R^2^* = .225). This was further supported by the difference in BIC (ΔBIC = 4.99), which slightly favored the FRN model over the P3 model (see [Table S13](#_Table_S13:_GLMM) for detailed GLMM results).

## Computational modelling results

### Model comparison

For the active learning data, model selection based on BIC scores revealed that M_CIPC_ explained choice behavior best (mean BIC = 152.52.79) with ΔBIC ≥ 5.08, suggesting positive to strong evidence that M_CIPC_ predicted choice behavior better than the alternative models (Raftery, 1995). Also for the observational learning data, M_CIPC_ was the best fitting model (mean BIC = 147.97), with ΔBIC ≥ 9.12 indicating strong evidence that this model explained choice behavior better than the other tested models. Thus, the winning active (M_act_) and observational model (M_obs_) contained separate learning rates for win and loss feedback ($\alpha^{+/-}$), a CIPC parameter for choice updating in no-feedback trials ($\varepsilon$), as well as an inverse temperature parameter ($\beta$).

### Model validation

For both M_act_ and M_obs_, Pearson correlations between free model parameters were generally quite low (M_act_: |*r*| ≤ .36; M_obs_: |*r*| ≤ .37), suggesting overall good parameter identifiability. Moreover, parameter recovery for M_act_ suggested that free model parameters were overall well recovered (0.79 ≤ *r* ≤ .94), with no spurious inter-correlations (|*r*| ≤ .44, see [Figure 2a](#_Figure_S2a:_Active)). Importantly, these results remained unchanged when applying model validation for fully simulated data sets, i.e., both simulated learning and test/transfer trial performance (see [Figure S2b](#_Figure_S2b:_Active)). Moreover, parameter recovery for M_obs_ likewise suggested good recoverability for M_obs_ (0.79 ≤ *r* ≤ .98), with inter-correlations again generally suggesting adequate parameter identifiability (|*r*| ≤ .51; but see [Figure 3](#_Figure_S2:_Observational)).

### Parameter estimates

Free model parameters were compared using linear model analyses. As with LMMs, also linear models involve a priori contrast coding. In accordance with the (G)LMM analyses, the categorical predictor group was deviation coded, with OCD set as the reference level. This allowed testing whether parameter estimates differed between OCD patients and HCs or OCD patients and SAD patients. Moreover, agency served as an additional categorical predictor.

Linear model analysis on learning rates yielded a main effect of feedback valence, *t*(320) = -4.18, *p* < .001, *b* = -0.14 (95% CI = -0.20 – -0.07), with larger learning rates for positive versus negative feedback (i.e., $\alpha^{+}$ > $\alpha^{-}$), thus confirming optimistic belief updating, though no group differences reached statistical significance (*p*s ≥ .099). Moreover, there were no group differences for neither the CIPC parameter $\varepsilon$ (*p*s ≥ .139) nor the inverse temperature parameter $\beta$ (*p*s ≥ .115).

## Model-based P3 analysis

Inclusion of the SPE predictor increased the proportion of variance explained from conditional *R^2^ =*.263 to conditional *R^2^=* .317, ΔBIC = 1574. The P3 signaled general unexpectedness, reflected in a significant main effect of the SPE, *t*(55.02) = 4.69, *p* < .001, *b* = 1.48 (95% CI = 0.85 – 2.11), where P3 amplitudes were enhanced with increasing unexpectedness. Importantly, the SPE was further modulated by agency, *t*(63.48) = -2.04, *p* = .045, *b* = -1.32 (95% CI = -2.62 – -0.03), indicating that the P3 signaled the SPE in active, *t*(51.79) = 3.94, *p_adj_* <.001, *b* = 2.14 (95% CI = 0.89 – 3.39), and observational learning *t*(51.59) = 2.41, *p_adj_* =.020, *b* = 0.81 (95% CI = 0.34 – 1.60), though SPE coding was stronger for active learning (*p* = .045). Moreover, a significant feedback valence × SPE interaction effect, *t*(59.39) = -4.82, *p* < .001, *b* = -7.91 (95% CI = -11.20 – -4.62), indicated that the P3 signaled a positive PE (higher amplitudes for feedback better than expected), *t*(57.46) = 5.61, *p_adj_* < .001, *b* = 5.43 (95% CI = 3.21 – 7.66), and negative PE (decreased amplitudes for feedback worse than expected), *t*(59.81) = -3.17, *p_adj_* = .002, *b* = -2.48 (95% CI = -4.28 – -0.68). Finally, this effect was further modulated by agency, *t*(57.75) = 9.86, *p* = .002, *b* = 3.26 (95% CI = 3.80 – 15.91), suggesting both positive, *t*(50.18) = 5.17, *p_adj_* < .001, *b* = 8.56 (95% CI = 4.73 – 12.39), and negative PE coding, *t*(46.37) = -3.54, *p_adj_* < .001, *b* = -4.28 (95% CI = -7.08 – -1.48), for active learning, whereas for observational learning, the P3 only reflected a positive, *t*(55.43) = 2.63, *p_adj_* = .022, *b* = 2.31 (95% CI = 4.33 – 2.63) but not negative PE (*p_adj_* = .461). There were no significant group effects (*p*s ≥ .089). Detailed LMM results are reported in [Table S12.](#_Table_S12:_LMM)

## Relationship with depressive symptoms

### Choice behavior

#### Task performance in test trials

Transdiagnostic-dimensional GLMM analysis using the BDI-II revealed a significant main effect of BDI-II scores on choice accuracy where higher depressive symptoms predicted overall decreased task performance, *z* = -2.65, *p* = .008, *b* = -0.03 (95% CI = -0.05 – -0.01). Moreover, the BDI-II × contingency interaction effect reached statistical significance, *z* = 2.07, *p* = .039, *b* = 0.02 (95% CI = 0.00 – 0.03), suggesting that with higher reward contingency BDI-II scores more strongly affected learning performance by predicting decreased choice accuracy.

#### Win-stay/lose-shift behavior in learning trials

Depressive symptoms also modulated win-stay/lose-shift behavior. Accordingly, transdiagnostic-dimensional GLMM analysis yielded a significant main effect of BDI-II scores on shifting behavior, *z* = 3.36, *p* = .001, *b* = 0.02 (95% CI = 0.01 – 0.04), indicating that higher depressive symptoms predicted generally more choice shifting. Furthermore, there was a significant BDI-II × previous feedback valence × previous feedback authenticity interaction effect, *z* = 4.62, *p* < .001, *b* = 0.21 (95% CI = 0.12 – 0.30). Resolving this interaction revealed that the positive relationship between depressive symptoms and choice shifting was more pronounced following authentic compared to misleading wins, *z* = -3.06, *p_adj_* = .002, *b* = -0.02 (95% CI = -0.03 – -0.01). Conversely, for losses, the positive relationship between depressive symptoms and choice shifting was stronger following misleading compared to authentic losses, *z* = 2.93, *p_adj_* = .003, *b* = 0.09 (95% CI = 0.00 – 0.02). Thus, depressive symptoms particularly interfered with staying following authentic positive as well as misleading negative feedback.

### EEG

#### Feedback-related negativity (FRN)

Model-free transdiagnostic-dimensional FRN analysis indicated that feedback processing was also modulated by depressive symptoms. Accordingly, the LMM yielded a significant main effect of BDI-II scores, suggesting overall reduced, i.e., more positive, FRN amplitudes with higher depressive symptoms, *t*(78.20) = 2.53, *p* = .013, *b* = 0.11 (95% CI = 0.02 – 0.20). Dimensional model-based FRN analysis even revealed a significant BDI-II × agency × feedback valence × SPE interaction effect, *t*(71.42) = -2.04, *p* = .045, *b* = -0.46 (95% CI = -0.92 – -0.01). Probing this effect using a spotlight approach showed that while in active learning participants with low BDI-II scores showed intact FRN PE coding of both positive, *t*(60.65) = 22.99, *p_adj_* = .008, *b* = 2.05 (95% CI = 0.84 – 11.38), and negative PEs, *t*(58.01) = -4.12, *p_adj_* = .001, *b* = -5.47 (95% CI = -8.90 – 2.05), FRN amplitudes in participants scoring high on the BDI-II did not reflect a PE (*ps_adj_* ≥ .477). During observational learning, the FRN did not reflect PEs in either participants scoring low or high on the BDI-II (*ps_adj_* ≥ .658).

#### P3

Depressive symptoms also appeared to have a modulating effect on the P3. Specifically, transdiagnostic-dimensional LMM analysis revealed a significant main effect of BDI-II suggesting that P3 amplitudes were generally increased, i.e., more positive with higher depressive symptoms, *t*(79.28) = 2.16, *p* = .034, *b* = 0.11 (95% CI = 0.01 – 0.22). However, when adding the predictor SPE as a covariate, all BDI-II-related effects vanished (*ps* ≥ .153).

#### Correlation between obsessive-compulsive and depressive symptoms

To assess the relationship between obsessive-compulsive and depressive symptoms, we calculated Spearman’s rank correlation coefficient between participants’ OCI-R and BDI-II scores. Results revealed a moderate positive relationship, *r*(81) = .57, *p* < .001. Accordingly, OCI-R and BDI-II were not fully independent and/or may be influenced by shared, transdiagnostic mechanisms.

# References

Bellebaum, C., Rustemeier, M., & Daum, I. (2012). Positivity effect in healthy aging in observational but not active feedback-learning. *Aging, Neuropsychology, and Cognition 19*(3), 402-420.

Burnside, R., Fischer, A. G., & Ullsperger, M. (2019). The feedback‐related negativity indexes prediction error in active but not observational learning. *Psychophysiology, 56*(9), e13389.

Collins, A. G. (2018). The tortoise and the hare: Interactions between reinforcement learning and working memory. *Journal of Cognitive Neuroscience 30*(10), 1422-1432.

Colosio, M., Shestakova, A., Nikulin, V. V., Blagovechtchenski, E., & Klucharev, V. (2017). Neural mechanisms of cognitive dissonance (revised): An EEG study. *Journal of Neuroscience 37*(20), 5074-5083.

Dercon, Q., Mehrhof, S. Z., Sandhu, T. R., Hitchcock, C., Lawson, R. P., Pizzagalli, D. A., Dalgleish, T., & Nord, C. L. (2024). A core component of psychological therapy causes adaptive changes in computational learning mechanisms. *Psychological Medicine 54*(2), 327-337.

Enders, C. K., & Tofighi, D. (2007). Centering predictor variables in cross-sectional multilevel models: a new look at an old issue. *Psychological Methods 12*(2), 121.

Festinger, L. (1957). A theory of cognitive dissonance. *Evanston, IL: Row and Peterson*

Frank, M. J., Moustafa, A. A., Haughey, H. M., Curran, T., & Hutchison, K. E. (2007). Genetic triple dissociation reveals multiple roles for dopamine in reinforcement learning. *Proceedings of the National Academy of Sciences 104*(41), 16311-16316.

Frank, M. J., Seeberger, L. C., & O'reilly, R. C. (2004). By carrot or by stick: cognitive reinforcement learning in parkinsonism. *Science 306*(5703), 1940-1943.

Hochberg, Y. (1987). *Multiple comparison procedures*. John Wiley & Sons, Inc. .

Kurtenbach, H., Ort, E., Froböse, M. I., & Jocham, G. (2022). Removal of reinforcement improves instrumental performance in humans by decreasing a general action bias rather than unmasking learnt associations. *PLoS Computational Biology 18*(12), e1010201.

Lefebvre, G., Lebreton, M., Meyniel, F., Bourgeois-Gironde, S., & Palminteri, S. (2017). Behavioural and neural characterization of optimistic reinforcement learning. *Nature Human Behaviour 1*(4), 0067.

LoTemplio, S. B., Lopes, C. L., McDonnell, A. S., Scott, E. E., Payne, B. R., & Strayer, D. L. (2023). Updating the relationship of the Ne/ERN to task-related behavior: A brief review and suggestions for future research. *Frontiers in Human Neuroscience 17*, 1150244.

Luettgau, L., Tempelmann, C., Kaiser, L. F., & Jocham, G. (2020). Decisions bias future choices by modifying hippocampal associative memories. *Nature Communications 11*(1), 3318.

Moritz, S., & Pohl, R. F. (2009). Biased processing of threat-related information rather than knowledge deficits contributes to overestimation of threat in obsessive-compulsive disorder. *Behavior Modification 33*(6), 763-777.

Palminteri, S., Lefebvre, G., Kilford, E. J., & Blakemore, S.-J. (2017). Confirmation bias in human reinforcement learning: Evidence from counterfactual feedback processing. *PLoS Computational Biology 13*(8), e1005684.

Ptasczynski, L. E., Steinecker, I., Sterzer, P., & Guggenmos, M. (2022). The value of confidence: Confidence prediction errors drive value-based learning in the absence of external feedback. *PLoS Computational Biology 18*(10), e1010580.

Raftery, A. E. (1995). Bayesian model selection in social research. *Sociological Methodology* 111-163.

Scheffe, H. (1999). *The analysis of variance* (Vol. 72). John Wiley & Sons.

Schmider, E., Ziegler, M., Danay, E., Beyer, L., & Bühner, M. (2010). Is it really robust? *Methodology*

Schultner, D. T., Lindström, B. R., Cikara, M., & Amodio, D. M. (2024). Transmission of social bias through observational learning. *Science Advances 10*(26), eadk2030.

Sharot, T., & Garrett, N. (2016). Forming beliefs: Why valence matters. *Trends in Cognitive Sciences 20*(1), 25-33.

Sutton, R. S., & Barto, A. G. (2018). *Reinforcement learning: An introduction*. MIT press.

van Dinteren, R., Arns, M., Jongsma, M. L., & Kessels, R. P. (2014). P300 development across the lifespan: a systematic review and meta-analysis. *PloS One 9*(2), e87347.

Watkins, C. J., & Dayan, P. (1992). Q-learning. *Machine Learning 8*, 279-292.

Wilson, R. C., & Collins, A. G. (2019). Ten simple rules for the computational modeling of behavioral data. *Elife 8*, e49547.

Zetsche, U., Rief, W., & Exner, C. (2015). Individuals with OCD lack unrealistic optimism bias in threat estimation. *Behavior Therapy 46*(4), 510-520.

Zhu, J., Hashimoto, J., Katahira, K., Hirakawa, M., & Nakao, T. (2021). Computational modeling of choice-induced preference change: A Reinforcement-Learning-based approach. *PLoS One 16*(1), e0244434.

Zhu, J., Katahira, K., Hirakawa, M., & Nakao, T. (2024). Externally provided rewards increase internal preference, but not as much as preferred ones without extrinsic rewards. *Computational Brain & Behavior* 1-21.

# Table S1: Comorbid diagnoses

*Current and past comorbid diagnoses in patient groups.*

|  | | | OCD (*n* = 20) | SAD (*n* = 21) |
| --- | --- | --- | --- | --- |
| Current | | |  |  |
|  | *Affective/mood disorders* | |  |  |
|  |  | Major depressive disorder, single episode, mild | 3 | − |
|  |  | Major depressive disorder, single episode, moderate | − | 2 |
|  |  | Major depressive disorder, recurrent, mild | 2 | 1 |
|  |  | Major depressive disorder, recurrent, moderate | 1 | − |
|  |  | Dysthymic disorder | 5 | 3 |
|  |  | Mood disorder due to known physiological condition with major depressive-like episode | 1 | − |
|  | *Anxiety disorders* | |  |  |
|  |  | Panic disorder | 2 | 2 |
|  |  | Agoraphobia | − | 5 |
|  |  | Generalized anxiety disorder | 3 | 6 |
|  |  | Post-traumatic stress disorder | 1 | − |
|  | *Other* | |  |  |
|  |  | Attention-deficit hyperactivity disorder | 1 | 1 |
| Past | | |  |  |
|  | *Affective/mood disorders* | |  |  |
|  |  | Major depressive disorder, single episode, in partial remission | 2 | − |
|  |  | Major depressive disorder, single episode, in full remission | 1 | 2 |
|  |  | Major depressive disorder, recurrent, in partial remission | 3 | 4 |
|  |  | Major depressive disorder, recurrent, in full remission | 2 | 6 |
|  | *Anxiety disorders* | |  |  |
|  |  | Panic disorder | 3 | 6 |
|  |  | Post-traumatic stress disorder | − | 2 |

*Note*: Data are presented as the number of patients fulfilling the criteria for a diagnostic category. Diagnoses were assessed using the Structured Clinical Interview for DSM-5 – Clinician Version (DSM-5-CV). Abbreviations: OCD = obsessive-compulsive disorder; SAD = social anxiety disorder. Seven patients in the OCD group and eight patients in the SAD group did not meet the criteria for any comorbid diagnosis.

# Table S2: Medication

*Prescription and current use of psychotropic medication in patient groups.*

|  | | OCD (*n* = 18) | SAD (*n* = 14) |
| --- | --- | --- | --- |
| *Selective serotonin-reuptake-inhibitors (SSRIs)* | |  |  |
|  | Paroxetine | 2 | − |
|  | Sertraline | 8 | − |
|  | Fluoxetine | 1 | 2 |
|  | Escitalopram | 2 | 5 |
|  | Citalopram | 1 | 1 |
| *Norepinephrine-dopamine-reuptake-inhibitors (NDRIs)* | |  |  |
|  | Methylphenidate | − | 1 |
| *Selective serotonin-noradrenalin-reuptake-inhibitors (SSNRIs)* | |  |  |
|  | Venlafaxine | 2 | 3 |
| *Tricyclic antidepressants* | |  |  |
|  | Clomipramine | 1 | − |
| *Noradrenergic and specific serotonergic antidepressants (NaSSAs)* | |  |  |
|  | Mirtazapine | 1 | − |
| *Anxiolytics* | |  |  |
|  | Buspirone | − | 2 |
| *(Atypical) antipsychotics* | |  |  |
|  | Quetiapine | 4 | 2 |
|  | Clozapine | − | 1 |
|  | Promethazine | − | 1 |
| *Other* | |  |  |
|  | Oxycodone | 1 | − |
|  | Levodopa/Benserazide | 1 | − |

*Note*: Data are presented as the number of patients receiving each respective medication. Abbreviations: OCD = obsessive-compulsive disorder; SAD = social anxiety disorder. Nine patients in the OCD group and 15 patients in the SAD group reported that they were currently not taking any medication.

# Table S3: Artifact rejection during EEG preprocessing

| *Mean percent (%) excluded segments during artifact correction.* | | | |
| --- | --- | --- | --- |
|  | | Active Learning | Observational Learning |
| *Feedback-locked* | |  |  |
|  | win | 1.52 (2.53) | 2.20 (3.11) |
|  | loss | 1.55 (2.89) | 2.30 (3.19) |
| *Response-locked* | |  |  |
|  | correct | 1.74 (2.90) |  |
|  | incorrect | 1.53 (2.84) |  |
| *Note*: Data are presented as mean percent with standard deviations (*SD*) in parentheses. | | | |

# Table S4a: GLMM results on choice accuracy (test phase)

| *Inferential Statistics from Generalized Linear Mixed-Effects Model Analyses on Test Phase Performance* | | | | | | | | | | | | | | | | | | |  |
| --- | --- | --- | --- | --- | --- | --- | --- | --- | --- | --- | --- | --- | --- | --- | --- | --- | --- | --- | --- |
|  | | *1. Categorical Model* | | | | | | | |  | *2. Transdiagnostic-Dimensional Model* | | | | | | | | |
| *Model Formula* | | choice accuracy ~ group * agency * contingency * block + (1 + agency * contingency * block \| participant) | | | | | | | |  | choice accuracy ~ OCI-R * agency * contingency * block + (1 + agency * contingency * block \| participant) | | | | | | | | |
|  | |  | | | | | | | |  |  | | | | | | | | |
| *Sampling Units* | |  | | | | | | | |  |  | | | | | | | | |
|  | | *N* observations = 19910 | | | | | | | |  | *N* observations = 19910 | | | | | | | | |
|  | | *N* participants = 83 (27 OCD, 27 HC, 29 SAD) | | | | | | | |  | *N* participants = 83 (27 OCD, 27 HC, 29 SAD) | | | | | | | | |
|  | |  | | | | | | | |  |  | | | | | | | | |
| *Fixed Effects* | |  | | | | | | | |  |  | | | | | | | | |
|  | | *b* | 95% CI | | | | *z* | | *p* |  | *b* | 95% CI | | | | *z* | | *p* | |
| **Intercept** | | **1.65** | **1.42 – 1.89** | | | | **13.71** | | **< .001** |  | **1.65** | **1.40 – 1.89** | | | | **13.10** | | **< .001** | |
| Group/OCI-R | |  |  | | | |  | |  |  | -0.01 | -0.03 – 0.00 | | | | -1.42 | | .156 | |
|  | ***OCD vs. HC*** | **0.73** | **0.15 – 1.31** | | | | **2.47** | | **.014** |  |  |  | | | |  | |  | |
|  | *OCD vs. SAD* | -0.10 | -0.67 – 0.47 | | | | -0.34 | | .734 |  |  |  | | | |  | |  | |
| Agency | | 0.11 | -0.32 – 0.54 | | | | 0.51 | | .611 |  | 0.11 | -0.32 – 0.54 | | | | 0.50 | | .616 | |
| **Contingency** | | **-0.71** | **-0.89 – -0.53** | | | | **-7.65** | | **< .001** |  | **-0.71** | **-0.90 – -0.53** | | | | **-7.57** | | **< .001** | |
| **Block** | | **0.24** | **0.15 – 0.33** | | | | **5.19** | | **< .001** |  | **0.24** | **0.15 – 0.33** | | | | **5.18** | | **< .001** | |
| Group/OCI-R × Agency | |  |  | | | |  | |  |  | 0.01 | -0.02 – 0.04 | | | | 0.55 | | .586 | |
|  | *OCD vs. HC* | 0.05 | -0.99 – 1.10 | | | | 0.10 | | .918 |  |  |  | | | |  | |  | |
|  | *OCD vs. SAD* | 0.06 | -0.97 – 1.08 | | | | 0.11 | | .914 |  |  |  | | | |  | |  | |
| **Group/OCI-R × Contingency** | |  |  | | | |  | |  |  | **0.01** | **0.00 – 0.03** | | | | **2.09** | | **.037** | |
|  | ***OCD vs. HC*** | **-0.56** | **-1.01 – -0.11** | | | | **-2.46** | | **.014** |  |  |  | | | |  | |  | |
|  | *OCD vs. SAD* | -0.27 | -0.70 – 0.17 | | | | -1.21 | | .227 |  |  |  | | | |  | |  | |
| Agency × Contingency | | 0.25 | -0.12 – 0.63 | | | | 1.33 | | .183 |  | 0.25 | -0.13 – 0.63 | | | | 1.29 | | .196 | |
| Group/OCI-R × Block | |  |  | | | |  | |  |  | 0.00 | -0.01 – 0.00 | | | | -1.01 | | .311 | |
|  | *OCD vs. HC* | 0.09 | -0.13 – 0.31 | | | | 0.81 | | .419 |  |  |  | | | |  | |  | |
|  | *OCD vs. SAD* | 0.08 | -0.13 – 0.28 | | | | 0.72 | | .471 |  |  |  | | | |  | |  | |
| Agency × Block | | 0.00 | -0.19 – 0.19 | | | | 0.00 | | .997 |  | -0.01 | -0.20 – 0.19 | | | | -0.05 | | .958 | |
| Contingency × Block | | -0.06 | -0.16 – 0.05 | | | | -0.99 | | .324 |  | -0.06 | -0.17 – 0.05 | | | | -1.01 | | .312 | |
| Group/OCI-R × Agency × Contingency | |  |  | | | |  | |  |  | 0.02 | -0.01 – 0.04 | | | | 1.11 | | .226 | |
|  | *OCD vs. HC* | -0.43 | -1.34 – 0.49 | | | | -0.91 | | .363 |  |  |  | | | |  | |  | |
|  | *OCD vs. SAD* | -0.85 | -1.74 – 0.03 | | | | -1.90 | | .059 |  |  |  | | | |  | |  | |
| Group/OCI-R × Agency × Block | |  |  | | | |  | |  |  | 0.00 | -0.01 – 0.01 | | | | -0.08 | | .934 | |
|  | *OCD vs. HC* | -0.25 | -0.70 – 0.20 | | | | -1.09 | | .275 |  |  |  | | | |  | |  | |
|  | *OCD vs. SAD* | -0.05 | -0.48 – 0.38 | | | | -0.22 | | .824 |  |  |  | | | |  | |  | |
| Group/OCI-R × Contingency × Block | |  |  | | | |  | |  |  | 0.00 | -0.00 – 0.01 | | | | 0.77 | | .443 | |
|  | *OCD vs. HC* | 0.07 | -0.19 – 0.33 | | | | 0.52 | | .605 |  |  |  | | | |  | |  | |
|  | *OCD vs. SAD* | -0.08 | -0.33 – 0.17 | | | | -0.65 | | .518 |  |  |  | | | |  | |  | |
| Agency × Contingency × Block | | 0.08 | -0.14 – 0.30 | | | | 0.70 | | .485 |  | 0.08 | -0.15 – 0.30 | | | | 0.65 | | .513 | |
| Group/OCI-R × Agency × Contingency × Block | |  |  | | | |  | |  |  | -0.01 | -0.03 – 0.01 | | | | -1.12 | | .261 | |
|  | *OCD vs. HC* | 0.38 | -0.14 – 0.91 | | | | 1.42 | | .155 |  |  |  | | | |  | |  | |
|  | *OCD vs. SAD* | -0.30 | -0.80 – 0.21 | | | | -1.15 | | .252 |  |  |  | | | |  | |  | |
|  | | | | | | | | | |  |  | | | | | | | | |
| *Random Effects* | |  | | | | | | | |  |  | | | | | | | | |
|  | | Variance | *SD* | | Correlation | | | | |  | Variance | *SD* | | Correlation | | | | | |
| Participant (Intercept) | | 1.12 | 1.06 | |  | | | | |  | 1.23 | 1.11 | |  | | | | | |
| Participant (Agency) | | 3.61 | 1.90 | | 0.11 | | | | |  | 3.61 | 1.90 | | 0.12 | | | | | |
| Participant (Contingency) | | 0.61 | 0.78 | | -0.45 -0.26 | | | | |  | 0.62 | 0.79 | | -0.47 -0.26 | | | | | |
| Participant (Block) | | 0.12 | 0.35 | | 0.08 0.09 -0.01 | | | | |  | 0.12 | 0.35 | | 0.06 0.10 -0.01 | | | | | |
| Participant (Agency × Contingency) | | 2.56 | 1.60 | | 0.11 -0.03 0.14 -0.10 | | | | |  | 2.63 | 1.62 | | 0.14 -0.04 0.13 -0.12 | | | | | |
| Participant (Agency × Block) | | 0.55 | 0.74 | | -0.18 0.19 0.35 0.49 -0.03 | | | | |  | 0.57 | 0.75 | | -0.24 0.18 0.39 0.46 -0.04 | | | | | |
| Participant (Contingency × Block) | | 0.18 | 0.42 | | -0.01 0.09 -0.14 -0.14 0.06 -0.42 | | | | |  | 0.18 | 0.42 | | 0.07 0.09 -0.19 -0.12 0.07 -0.42 | | | | | |
| Participant (Agency × Contingency × Block) | | 0.72 | 0.85 | | -0.17 0.13 -0.19 -0.14 0.07 -0.06 -0.03 | | | | |  | 0.79 | 0.89 | | -0.06 0.14 -0.20 -0.16 0.12 -0.10 0.00 | | | | | |
|  | |  | | | | | | | |  |  | | | | | | | | |
| *Goodness of fit* | |  | | | | | | | |  |  | | | | | | | | |
|  | | AIC | | BIC | | LL | | Deviance | |  | AIC | | BIC | | LL | | Deviance | | |
|  | | 18496.68 | | 18970.62 | | -9188.34 | | 18376.68 | |  | 18494.94 | | 18905.69 | | -9195.47 | | 18390.94 | | |
|  | | Marginal R^2^ | | | | Conditional R^2^ | | | |  | Marginal R^2^ | | | | Conditional R^2^ | | | | |
|  | | 0.087 | | | | 0.556 | | | |  | 0.068 | | | | 0.557 | | | | |
| *Note:* Abbreviations: HC = healthy controls; OCD = obsessive-compulsive disorder; SAD = social anxiety disorder; CI = confidence interval; SD = standard deviation; AIC = Akaike information criterion; BIC = Bayesian information criterion; LL = log-likelihood. Significant effects are highlighted in bold. | | | | | | | | | | | | | | | | | | | |

# Table S4b: GLMM results on choice accuracy (learning phase in active learning)

| *Inferential Statistics from Generalized Linear Mixed-Effects Model Analyses on Learning Phase Performance in Active Learning* | | | | | | | | | | | | | | | | |
| --- | --- | --- | --- | --- | --- | --- | --- | --- | --- | --- | --- | --- | --- | --- | --- | --- |
|  | | *1. Categorical Model* | | | | | | |  | *2. Transdiagnostic-Dimensional Model* | | | | | | |
| *Model Formula* | | choice accuracy ~ group * contingency * block + (1 + contingency * block \| participant) | | | | | | |  | choice accuracy ~ OCI-R * contingency * block + (1 + contingency * block \| participant) | | | | | | |
|  | |  | | | | | | |  |  | | | | | | |
| *Sampling Units* | |  | | | | | | |  |  | | | | | | |
|  | | *N* observations = 19841 | | | | | | |  | *N* observations = 19841 | | | | | | |
|  | | *N* participants = 83 (27 OCD, 27 HC, 29 SAD) | | | | | | |  | *N* participants = 83 (27 OCD, 27 HC, 29 SAD) | | | | | | |
|  | |  | | | | | | |  |  | | | | | | |
| *Fixed Effects* | |  | | | | | | |  |  | | | | | | |
|  | | *b* | 95% CI | | | *z* | | *p* |  | *b* | 95% CI | | | *z* | | *p* |
| **Intercept** | | **1.17** | **0.96 – 1.37** | | | **11.01** | | **<.001** |  | **1.16** | **0.95 – 1.37** | | | **10.76** | | **<.001** |
| Group/OCI-R | |  |  | | |  | |  |  | -0.01 | -0.03 – 0.00 | | | -1.41 | | .159 |
|  | *OCD vs. HC* | 0.50 | -0.02 – 1.01 | | | 1.90 | | .058 |  |  |  | | |  | |  |
|  | *OCD vs. SAD* | -0.07 | -0.57 – 0.43 | | | -0.29 | | .773 |  |  |  | | |  | |  |
| **Contingency** | | **-0.62** | **-0.78 – -0.46** | | | **-7.76** | | **<.001** |  | **-0.62** | **-0.78 – -0.46** | | | **-7.59** | | **<.001** |
| **Block** | | **0.30** | **0.20 – 0.39** | | | **6.24** | | **<.001** |  | **0.30** | **0.20 – 0.39** | | | **5.99** | | **<.001** |
| Group/OCI-R × Contingency | |  |  | | |  | |  |  | 0.01 | -0.00 – 0.02 | | | 1.70 | | .089 |
|  | ***OCD vs. HC*** | **-0.39** | **-0.78 – -0.00** | | | **-1.97** | | **.049** |  |  |  | | |  | |  |
|  | *OCD vs. SAD* | 0.02 | -0.36 – 0.39 | | | 0.09 | | .927 |  |  |  | | |  | |  |
| **Group/OCI-R × Block** | |  |  | | |  | |  |  | 0.00 | -0.01 – 0.00 | | | -1.07 | | .282 |
|  | ***OCD vs. HC*** | **0.24** | **0.01 – 0.47** | | | **2.02** | | **.043** |  |  |  | | |  | |  |
|  | *OCD vs. SAD* | -0.04 | -0.27 – 0.18 | | | -0.38 | | .702 |  |  |  | | |  | |  |
| **Contingency × Block** | | **-0.14** | **-0.23 – -0.05** | | | **-2.94** | | **.003** |  | **-0.14** | **-0.23 – -0.04** | | | **-2.89** | | **.004** |
| Group/OCI-R × Contingency × Block | |  |  | | |  | |  |  | 0.00 | --0.01 – 0.01 | | | 0.00 | | .998 |
|  | *OCD vs. HC* | -0.01 | -0.23 – 0.22 | | | -0.08 | | .940 |  |  |  | | |  | |  |
|  | *OCD vs. SAD* | 0.12 | -0.10 – 0.33 | | | 1.07 | | .284 |  |  |  | | |  | |  |
|  | | | | | | | | |  |  | | | | | | |
| *Random Effects* | |  | | | | | | |  |  | | | | | | |
|  | | Variance | *SD* | Correlation | | | | |  | Variance | *SD* | Correlation | | | | |
| Participant (Intercept) | | 0.88 | 0.94 |  | | | | |  | 0.92 | 0.96 |  | | | | |
| Participant (Contingency) | | 0.46 | 0.68 | -0.34 | | | | |  | 0.48 | 0.69 | -0.37 | | | | |
| Participant (Block) | | 0.16 | 0.39 | 0.73 -0.19 | | | | |  | 0.17 | 0.41 | 0.74 -0.24 | | | | |
| Participant (Contingency × Block) | | 0.13 | 0.36 | -0.28 0.33 -0.32 | | | | |  | 0.14 | 0.37 | -0.29 0.35 -0.34 | | | | |
|  | | | | | | | | |  |  | | | | | | |
| *Goodness of fit* | |  | | | | | | |  |  | | | | | | |
|  | | AIC | BIC | | LL | | Deviance | |  | AIC | BIC | | LL | | Deviance | |
|  | | 21205.81 | 21379.51 | | -10580.9 | | 21161.81 | |  | 21204.77 | 21346.89 | | -10584.39 | | 21168.77 | |
|  | | Marginal R^2^ | | | | Conditional R^2^ | | |  | Marginal R^2^ | | | | Conditional R^2^ | | |
|  | | 0.093 | | | | 0.376 | | |  | 0.079 | | | | 0.376 | | |
| *Note:* Abbreviations: HC = healthy controls; OCD = obsessive-compulsive disorder; SAD = social anxiety disorder; CI = confidence interval; SD = standard deviation; AIC = Akaike information criterion; BIC = Bayesian information criterion; LL = log-likelihood. Significant effects are highlighted in bold. | | | | | | | | | | | | | | | | |

# Table S5: GLMM results on win-stay/lose-shift behavior

| *Inferential Statistics from Generalized Linear Mixed-Effects Model Analyses on Win-Stay/Lose-Shift Behavior* | | | | | | | | | | | | | | |
| --- | --- | --- | --- | --- | --- | --- | --- | --- | --- | --- | --- | --- | --- | --- |
|  | | *1. Categorical Model* | | | | | |  | *2. Transdiagnostic-Dimensional Model* | | | | | |
| *Model Formula* | | choice shift ~ group * previous feedback valence * previous feedback authenticity * block +  (1 + previous feedback valence * block \| participant) | | | | | |  | choice shift ~ OCI-R * previous feedback valence * previous feedback authenticity * block +  (1 + previous feedback valence * block \| participant) | | | | | |
|  | |  | | | | | |  |  | | | | | |
| *Sampling Units* | |  | | | | | |  |  | | | | | |
|  | | *N* observations = 18795 | | | | | |  | *N* observations = 18795 | | | | | |
|  | | *N* participants = 83 (27 OCD, 27 HC, 29 SAD) | | | | | |  | *N* participants = 83 (27 OCD, 27 HC, 29 SAD) | | | | | |
|  | |  | | | | | |  |  | | | | | |
| *Fixed Effects* | |  | | | | | |  |  | | | | | |
|  | | *b* | 95% CI | | | *z* | *p* |  | *b* | 95% CI | | *z* | | *p* |
| **Intercept** | | **-0.86** | **-1.01 – -0.71** | | | **-11.14** | **<.001** |  | **-0.86** | **-1.02 – -0.71** | | **-10.78** | | **<.001** |
| **Group/OCI-R** | |  |  | | |  |  |  | **0.01** | **0.00 – 0.02** | | **2.14** | | **.032** |
|  | ***OCD vs. HC*** | **-0.47** | **-0.85 – -0.10** | | | **-2.48** | **.013** |  |  |  | |  | |  |
|  | *OCD vs. SAD* | 0.04 | -0.32 – 0.41 | | | 0.24 | .813 |  |  |  | |  | |  |
| **Previous Feedback Valence** | | **0.86** | **0.70 – 1.02** | | | **10.30** | **<.001** |  | **0.86** | **0.70 – 1.02** | | **10.51** | | **<.001** |
| Previous Feedback Authenticity | | -0.06 | -0.13 – 0.02 | | | -1.37 | .170 |  | -0.06 | -0.14 – 0.02 | | -1.40 | | .161 |
| **Block** | | **-0.13** | **-0.20 – -0.06** | | | **-3.49** | **<.001** |  | **-0.13** | **-0.20 – -0.06** | | **-3.58** | | **<.001** |
| **Group/OCI-R × Previous Feedback Valence** | |  |  | | |  |  |  | **-0.01** | **-0.02 – -0.00** | | **-2.07** | | **.038** |
|  | *OCD vs. HC* | 0.30 | -0.10 – 0.70 | | | 1.46 | .144 |  |  |  | |  | |  |
|  | *OCD vs. SAD* | 0.23 | -0.15 – 0.61 | | | 1.18 | .239 |  |  |  | |  | |  |
| Group/OCI-R ×Previous Feedback Authenticity | |  |  | | |  |  |  | 0.00 | -0.01 – 0.00 | | -0.61 | | .539 |
|  | *OCD vs. HC* | -0.01 | -0.21 – 0.19 | | | -0.07 | .943 |  |  |  | |  | |  |
|  | *OCD vs. SAD* | -0.03 | -0.21 – 0.15 | | | -0.34 | .734 |  |  |  | |  | |  |
| Previous Feedback Valence × Previous Feedback Authenticity | | **-1.75** | **-1.91 – -1.59** | | | **-21.57** | **<.001** |  | **-1.71** | **-1.87 – -1.56** | | **-21.27** | | **<.001** |
| Group/OCI-R × Block | |  |  | | |  |  |  | 0.00 | -0.00 – 0.01 | | 0.90 | | .369 |
|  | *OCD vs. HC* | -0.04 | -0.22 – 0.13 | | | -0.49 | .624 |  |  |  | |  | |  |
|  | *OCD vs. SAD* | 0.00 | -0.17 – 0.17 | | | -0.01 | .992 |  |  |  | |  | |  |
| Previous Feedback Valence × Block | | -0.04 | -0.12 – 0.05 | | | -0.88 | .378 |  | -0.03 | -0.12 – 0.05 | | -0.79 | | .429 |
| Previous Feedback Authenticity × Block | | 0.05 | -0.02 – 0.12 | | | 1.37 | .172 |  | 0.05 | -0.02 – 0.12 | | 1.36 | | .174 |
| Group/OCI-R × Previous Feedback Valence × Previous Feedback Authenticity | |  |  | | |  |  |  | 0.00 | -0.01 – 0.01 | | 0.54 | | .589 |
|  | *OCD vs. HC* | -0.14 | -0.54 – 0.26 | | | -0.67 | .500 |  |  |  | |  | |  |
|  | ***OCD vs. SAD*** | **0.58** | **0.21 – 0.94** | | | **3.07** | **.002** |  |  |  | |  | |  |
| Group/OCI-R × Previous Feedback Valence × Block | |  |  | | |  |  |  | 0.00 | -0.00 – 0.01 | | 0.36 | | .718 |
|  | *OCD vs. HC* | -0.08 | -0.29 – 0.12 | | | -0.78 | .435 |  |  |  | |  | |  |
|  | *OCD vs. SAD* | -0.06 | -0.25 – 0.13 | | | -0.63 | .528 |  |  |  | |  | |  |
| Group/OCI-R × Previous Feedback Authenticity × Block | |  |  | | |  |  |  | 0.00 | -0.00 – 0.01 | | 0.47 | | .641 |
|  | *OCD vs. HC* | -0.06 | -0.23 – 0.12 | | | -0.63 | .531 |  |  |  | |  | |  |
|  | *OCD vs. SAD* | 0.01 | -0.15 – 0.18 | | | 0.18 | .857 |  |  |  | |  | |  |
| **Previous Feedback Valence × Previous Feedback Authenticity × Block** | | **-0.22** | **-0.37 – -0.08** | | | **-3.11** | **.002** |  | **-0.20** | **-0.34 – -0.06** | | **-2.81** | | **.005** |
| Group/OCI-R × Previous Feedback Valence × Previous Feedback Authenticity × Block | |  |  | | |  |  |  | 0.00 | -0.01 – 0.01 | | 0.51 | | .613 |
|  | *OCD vs. HC* | -0.18 | -0.53 – 0.18 | | | -0.98 | .329 |  |  |  | |  | |  |
|  | *OCD vs. SAD* | 0.20 | -0.13 – 0.52 | | | 1.17 | .244 |  |  |  | |  | |  |
|  |  | | | | | | |  |  | | | | | |
| *Random Effects* | |  | | | | | |  |  | | | | | |
|  | | Variance | *SD* | Correlation | | | |  | Variance | *SD* | Correlation | | | |
| Participant (Intercept) | | 0.45 | 0.67 |  | | | |  | 0.49 | 0.70 |  | | | |
| Participant (Previous Feedback Valence) | | 0.41 | 0.64 | -0.71 | | | |  | 0.40 | 0.63 | -0.69 | | | |
| Participant (Block) | | 0.08 | 0.28 | 0.66 -0.41 | | | |  | 0.08 | 0.28 | 0.65 -0.41 | | | |
| Participant (Previous Feedback Valence × Block) | | 0.03 | 0.18 | 0.30 -0.02 -0.37 | | | |  | 0.03 | 0.18 | 0.25 -0.01 -0.38 | | | |
|  | | | | | | | |  |  | | | | | |
| *Goodness of fit* | |  | | | | | |  |  | | | | | |
|  | | AIC | BIC | | LL | | Deviance |  | AIC | BIC | | LL | Deviance | |
|  | | 19952.68 | 20219.29 | | -9942.34 | | 19884.68 |  | 19966.38 | 20170.26 | | -9957.19 | 19914.38 | |
|  | | Marginal R^2^ | | | Conditional R^2^ | | |  | Marginal R^2^ | | | Conditional R^2^ | | |
|  | | 0.131 | | | 0.287 | | |  | 0.121 | | | 0.284 | | |
| *Note:* Abbreviations: HC = healthy controls; OCD = obsessive-compulsive disorder; SAD = social anxiety disorder; CI = confidence interval; SD = standard deviation; AIC = Akaike information criterion; BIC = Bayesian information criterion; LL = log-likelihood. Significant effects are highlighted in bold. | | | | | | | | | | | | | | |

# Table S6: GLMM results on learning propensity

| *Inferential Statistics from Generalized Linear Mixed-Effects Model Analyses of Transfer Phase Performance* | | | | | | |
| --- | --- | --- | --- | --- | --- | --- |
| *Model Formula* | | choice accuracy ~ group * agency * trial type  (1 + agency * trial type \| participant) | | | | |
|  |  |  | | | | |
| *Sampling Units* | |  | | | | |
|  | | *N* observations = 6078 | | | | |
|  | | *N* participants = 83 (27 OCD, 27 HC, 29 SAD) | | | | |
|  | |  | | | | |
| *Fixed Effects* | |  | | | | |
|  | | *b* | 95% CI | *z* | *p* | |
| **Intercept** | | **0.92** | **0.75 – 1.10** | **10.42** | **<.001** | |
| Group | |  |  |  |  | |
|  | *OCD vs. HC* | 0.09 | -0.34 – 0.52 | 0.42 | .677 | |
|  | *OCD vs. SAD* | -0.06 | -0.48 – 0.36 | -0.28 | .781 | |
| Agency | | 0.02 | -0.24 – 0.28 | 0.15 | .882 | |
| **Trial Type** | | **-0.37** | **-0.64 – -0.09** | **-2.59** | **.010** | |
| Group × Agency | |  |  |  |  | |
|  | *OCD vs. HC* | 0.14 | -0.50 – 0.78 | 0.43 | .666 | |
|  | *OCD vs. SAD* | 0.40 | -0.23 – 1.02 | 1.24 | .216 | |
| Group ×Trial Type | |  |  |  |  | |
|  | *OCD vs. HC* | 0.17 | -0.51 – 0.85 | 0.48 | .628 | |
|  | *OCD vs. SAD* | -0.01 | -0.68 – 0.65 | -0.04 | .967 | |
| Agency × Trial Type | | -0.16 | -0.67 – 0.36 | -0.59 | .553 | |
| Group × Agency × Trial Type | |  |  |  |  | |
|  | *OCD vs. HC* | -1.13 | -2.40 – 0.14 | -1.74 | .082 | |
|  | *OCD vs. SAD* | -1.07 | -2.31 – 0.17 | -1.69 | .091 | |
|  | | | | | | |
| *Random Effects* | |  | | | | |
|  | | Variance | *SD* | Correlation | | |
| Participant (Intercept) | | 0.52 | 0.72 |  | | |
| Participant (Agency) | | 0.95 | 0.97 | -0.09 | | |
| Participant (Trial Type) | | 1.17 | 1.08 | -0.25 -0.05 | | |
| Participant (Agency × Trial Type) | | 3.84 | 1.96 | -0.01 0.06 -0.07 | | |
|  | | | | | | |
| *Goodness of fit* | |  | | | | |
|  | | AIC | BIC | LL | | Deviance |
|  | | 6983.11 | 7130.78 | -3469.56 | | 6939.11 |
|  | | Marginal R^2^ | | Conditional R^2^ | | |
|  | | 0.014 | | 0.293 | | |
| Note: Abbreviations: OCD = obsessive compulsive disorder; HC = healthy controls; CI = confidence interval; SD = standard deviation; AIC = Akaike information criterion; BIC = Bayesian information criterion; LL = log-likelihood. Significant effects are highlighted in bold | | | | | | |

# Table S7: LMM results for the FRN (model-free)

| *Inferential Statistics of Model-Free FRN Linear Mixed-Effects Model Analyses* | | | | | | | | | | | | | | | | |
| --- | --- | --- | --- | --- | --- | --- | --- | --- | --- | --- | --- | --- | --- | --- | --- | --- |
|  | | *1. Categorical Model* | | | | | | | |  | *2. Transdiagnostic-Dimensional Model* | | | | | |
| *Model Formula* | | FRN ~ group * agency * feedback valence + (1 + agency * feedback valence \| participant) +  (1 \| electrode) | | | | | | | |  | FRN ~ OCI-R * agency * feedback valence + (1 + agency * feedback valence \| participant) +  (1 \| electrode) | | | | | |
|  |  |  | | | | | | | |  |  | | | | | |
| *Sampling Units* | |  | | | | | | | |  |  | | | | | |
|  | | *N* observations = 185974 | | | | | | | |  | *N* observations = 185974 | | | | | |
|  | | *N* participants = 81 (27 OCD, 27 HC, 27 SAD) | | | | | | | |  | *N* participants = 81 (27 OCD, 27 HC, 29 SAD) | | | | | |
|  | | *N* electrodes = 5 (Fz, FCz, FC1, FC2, Cz) | | | | | | | |  | *N* electrodes = 5 (Fz, FCz, FC1, FC2, Cz) | | | | | |
|  | |  | | | | | | | |  |  | | | | | |
| *Fixed Effects* | |  | | | | | | | |  |  | | | | | |
|  | | *b* | 95% CI | | | *t* | *df* | | *p* |  | *b* | 95% CI | | *t* | *df* | *p* |
| **Intercept** | | **3.41** | **2.02 – 4.79** | | | **5.24** | **14.83** | | **<.001** |  | **3.41** | **2.01 – 4.81** | | **5.18** | **5.18** | **<.001** |
| Group/OCI-R | |  |  | | |  |  | |  |  | 0.04 | -0.03 – 0.11 | | 1.20 | 1.20 | .235 |
|  | ***OCD vs. HC*** | **-2.87** | **-5.11 – -0.64** | | | **-2.56** | **77.17** | | **.012** |  |  |  | |  |  |  |
|  | *OCD vs. SAD* | -1.82 | -4.04 – 0.41 | | | -1.62 | 76.48 | | .109 |  |  |  | |  |  |  |
| **Agency** | | **-3.81** | **-4.64 – -2.97** | | | **-9.05** | **75.21** | | **<.001** |  | **-3.80** | **-4.64 – -2.96** | | **-9.03** | **-9.03** | **<.001** |
| **Feedback Valence** | | **-1.62** | **-2.09 – -1.16** | | | **-6.95** | **77.78** | | **<.001** |  | **-1.62** | **-2.09 – -1.16** | | **-6.92** | **-6.92** | **<.001** |
| Group/OCI-R × Agency | |  |  | | |  |  | |  |  | 0.00 | -0.06 – 0.06 | | -0.02 | -0.02 | .987 |
|  | *OCD vs. HC* | 1.11 | -0.96 – 3.17 | | | 1.07 | 75.86 | | .288 |  |  |  | |  |  |  |
|  | *OCD vs. SAD* | 0.21 | -1.83 – 2.25 | | | 0.21 | 74.53 | | .838 |  |  |  | |  |  |  |
| Group/OCI-R ×Feedback Valence | |  |  | | |  |  | |  |  | -0.03 | -0.06 – 0.00 | | -1.74 | -1.74 | .085 |
|  | ***OCD vs. HC*** | **1.20** | **0.06 – 2.34** | | | **2.10** | **78.18** | | **.039** |  |  |  | |  |  |  |
|  | *OCD vs. SAD* | 0.83 | -0.31 – 1.96 | | | 1.45 | 77.37 | | .151 |  |  |  | |  |  |  |
| **Agency × Feedback Valence** | | **1.86** | **1.21 – 2.51** | | | **6.70** | **77.43** | | **<.001** |  | **1.86** | **1.19 – 2.53** | | **5.54** | **5.54** | **<.001** |
| Group/OCI-R × Agency × Feedback Valence | |  |  | | |  |  | |  |  | 0.03 | -0.02 – 0.08 | | 1.15 | 1.15 | .252 |
|  | ***OCD vs. HC*** | **-2.10** | **-3.70 – -0.50** | | | **-2.61** | **78.10** | | **.011** |  |  |  | |  |  |  |
|  | *OCD vs. SAD* | -1.53 | -3.12 – 0.06 | | | -1.92 | 76.76 | | .059 |  |  |  | |  |  |  |
|  | | | | | | | | | |  |  | | | | | |
| *Random Effects* | |  | | | | | | | |  |  | | | | | |
|  | | Variance | *SD* | Correlation | | | | | |  | Variance | *SD* | Correlation | | | |
| Participant (Intercept) | | 16.80 | 4.10 |  | | | | | |  | 17.69 | 4.21 |  | | | |
| Participant (Agency) | | 13.79 | 3.71 | -0.27 | | | | | |  | 13.87 | 3.72 | -0.29 | | | |
| Participant (Feedback Valence) | | 4.20 | 2.05 | 0.01 0.01 | | | | | |  | 4.24 | 2.06 | -0.03 0.04 | | | |
| Participant (Agency × Feedback Valence) | | 7.84 | 2.80 | -0.06 -0.15 -0.60 | | | | | |  | 8.34 | 2.89 | 0.02 -0.18 -0.61 | | | |
| Electrode (Intercept) | | 1.07 | 1.03 |  | | |  | |  |  | 1.07 | 1.03 |  | |  |  |
| Residual | | 101.51 | 10.08 |  | | |  | |  |  | 101.51 | 10.08 |  | |  |  |
|  | | | | | | | | | |  |  | | | | | |
| *Goodness of fit* | |  | | | | | | | |  |  | | | | | |
|  | | AIC | BIC | | LL | | | Deviance | |  | AIC | BIC | | LL | Deviance | |
|  | | 1388281 | 1388524 | | -694116.4 | | | 1388233 | |  | 1388316 | 1388518 | | -694137.8 | 1388276 | |
|  | | Marginal R^2^ | | | Conditional R^2^ | | | | |  | Marginal R^2^ | | | Conditional R^2^ | | |
|  | | 0.049 | | | 0.225 | | | | |  | 0.040 | | | 0.224 | | |
| *Note:* Abbreviations: HC = healthy controls; OCD = obsessive-compulsive disorder; SAD = social anxiety disorder; CI = confidence interval; SD = standard deviation; AIC = Akaike information criterion; BIC = Bayesian information criterion; LL = log-likelihood. Significant effects are highlighted in bold. | | | | | | | | | | | | | | | | |

# Table S8: LMM results for the ERN/CRN (model-free)

| *Inferential Statistics of Model-Free ERN/CRN Linear Mixed-Effects Model Analyses* | | | | | | | | |
| --- | --- | --- | --- | --- | --- | --- | --- | --- |
| *Model Formula* | | ERN/CRN ~ group * accuracy * learning status +  (1 + accuracy * learning status \| participant) +  (1 \| electrode) | | | | | | |
|  |  |  | | | | | | |
| *Sampling Units* | |  | | | | | | |
|  | | *N* observations = 148590 | | | | | | |
|  | | *N* participants = 76 (26 OCD, 26 HC, 24 SAD) | | | | | | |
|  | | *N* electrodes = 5 (Fz, FCz, FC1, FC2, Cz) | | | | | | |
|  | |  | | | | | | |
| *Fixed Effects* | |  | | | | | | |
|  | | *b* | 95% CI | | *t* | *df* | | *p* |
| **Intercept** | | **-0.66** | **-0.99 – -0.33** | | **-3.97** | **64.57** | | **<.001** |
| Group | |  |  | |  |  | |  |
|  | *OCD vs. HC* | 0.29 | -0.46 – 1.04 | | 0.77 | 71.07 | | .445 |
|  | *OCD vs. SAD* | 0.37 | -0.39 – 1.13 | | 0.97 | 69.66 | | .336 |
| Accuracy | | -0.27 | -0.59 – 0.06 | | -1.65 | 65.48 | | .103 |
| **Learning Status** | | **-0.27** | **-0.53 – -0.00** | | **-2.03** | **60.34** | | **.047** |
| Group × Accuracy | |  |  | |  |  | |  |
|  | *OCD vs. HC* | -0.07 | -0.86 – 0.72 | | -0.18 | 66.40 | | .861 |
|  | *OCD vs. SAD* | 0.31 | -0.48 – 1.10 | | 0.78 | 63.81 | | .438 |
| Group ×Learning Status | |  |  | |  |  | |  |
|  | *OCD vs. HC* | 0.27 | -0.37 – 0.90 | | 0.83 | 61.40 | | .409 |
|  | *OCD vs. SAD* | 0.40 | -0.23 – 1.03 | | 1.27 | 59.26 | | .211 |
| **Accuracy × Learning Status** | | **-0.59** | **-1.17 – -0.00** | | **-2.01** | **64.13** | | **.049** |
| Group × Accuracy × Learning Status | |  |  | |  |  | |  |
|  | *OCD vs. HC* | -0.61 | -2.03 – 0.81 | | -0.86 | 65.32 | | .394 |
|  | *OCD vs. SAD* | 0.65 | -0.77 – 2.06 | | 0.91 | 62.49 | | .366 |
|  |  |  |  | |  |  | |  |
| *Random Effects* | |  |  | |  |  | |  |
|  | | Variance | *SD* | | Correlation | | | |
| Participant (Intercept) | | 1.74 | 1.32 | |  | | | |
| Participant (Accuracy) | | 1.60 | 1.27 | | 0.25 | | | |
| Participant (Learning Status) | | 0.90 | 0.95 | | 0.30 0.53 | | | |
| Participant (Accuracy × Learning Status) | | 4.86 | 2.21 | | 0.20 0.73 0.53 | | | |
| Electrode (Intercept) | | 0.02 | 0.13 | |  |  | |  |
| Residual | | 50.41 | 7.10 | |  |  | |  |
|  | |  |  | |  |  | |  |
| *Goodness of fit* | |  |  | |  |  | |  |
|  | | AIC | | BIC | LL | | Deviance | |
|  | | 1004926 | | 1005164 | -502439.2 | | 1004878 | |
|  | | Marginal R^2^ | | | Conditional R^2^ | | | |
|  | | 0.001 | | | 0.043 | | | |
| *Note:* Abbreviations: HC = healthy controls; OCD = obsessive-compulsive disorder; SAD = social anxiety disorder; CI = confidence interval; SD = standard deviation; AIC = Akaike information criterion; BIC = Bayesian information criterion; LL = log-likelihood. Significant effects are highlighted in bold. | | | | | | | | |

# Table S9: LMM results for the FRN (model-based)

| *Inferential Statistics of Model-Based FRN Linear Mixed-Effects Model Analyses* | | | | | | | | | | | | | | | | | | | | | | |  |
| --- | --- | --- | --- | --- | --- | --- | --- | --- | --- | --- | --- | --- | --- | --- | --- | --- | --- | --- | --- | --- | --- | --- | --- |
|  | | *1. Categorical Model* | | | | | | | | | |  | *2. Transdiagnostic-Dimensional Model* | | | | | | | | | | |
| *Model Formula* | | FRN ~ group * agency * feedback valence * SPE +  (1 + agency * feedback valence * SPE \| participant) +  (1 \| electrode) | | | | | | | | | |  | FRN ~ OCI-R * agency * feedback valence + (1 + agency * feedback valence * SPE \| participant) +  (1 \| electrode) | | | | | | | | | | |
|  |  |  | | | | | | | | | |  |  | | | | | | | | | | |
| *Sampling Units* | |  | | | | | | | | | |  |  | | | | | | | | | | |
|  | | *N* observations = 185974 | | | | | | | | | |  | *N* observations = 185974 | | | | | | | | | | |
|  | | *N* participants = 81 (27 OCD, 27 HC, 27 SAD) | | | | | | | | | |  | *N* participants = 81 (27 OCD, 27 HC, 29 SAD) | | | | | | | | | | |
|  | | *N* electrodes = 5 (Fz, FCz, FC1, FC2, Cz) | | | | | | | | | |  | *N* electrodes = 5 (Fz, FCz, FC1, FC2, Cz) | | | | | | | | | | |
|  | |  | | | | | | | | | |  |  | | | | | | | | | | |
| *Fixed Effects* | |  | | | | | | | | | |  |  | | | | | | | | | | |
|  | | *b* | 95% CI | | | | *t* | | *df* | | *p* |  | *b* | 95% CI | | | | *t* | | *df* | | *p* | |
| **Intercept** | | **3.88** | **2.46 – 5.31** | | | | **5.75** | | **16.98** | | **<.001** |  | **3.89** | **2.45 – 5.32** | | | | **5.70** | | **17.57** | | **<.001** | |
| Group/OCI-R | |  |  | | | |  | |  | |  |  | 0.05 | -0.03 – 0.12 | | | | 1.24 | | 79.99 | | .217 | |
|  | ***OCD vs. HC*** | **-2.74** | **-5.14 – -0.34** | | | | **-2.27** | | **77.69** | | **.026** |  |  |  | | | |  | |  | |  | |
|  | *OCD vs. SAD* | -1.91 | -4.31 – 0.49 | | | | -1.59 | | 77.06 | | .117 |  |  |  | | | |  | |  | |  | |
| **Agency** | | **-4.66** | **-5.72 – -3.59** | | | | **-8.69** | | **78.83** | | **<.001** |  | **-4.65** | **-5.71 – -3.59** | | | | **-8.73** | | **79.42** | | **<.001** | |
| **Feedback Valence** | | **-1.86** | **-2.35 – -1.37** | | | | **-7.58** | | **69.50** | | **<.001** |  | **-1.86** | **-2.36 – -1.37** | | | | **-7.57** | | **70.97** | | **<.001** | |
| SPE | | 0.37 | -0.21 – 0.96 | | | | 1.28 | | 56.91 | | .207 |  | 0.44 | -0.13 – 1.02 | | | | 1.55 | | 57.55 | | .126 | |
| Group/OCI-R × Agency | |  |  | | | |  | |  | |  |  | 0.01 | -0.07 – 0.09 | | | | 0.29 | | 81.58 | | .770 | |
|  | *OCD vs. HC* | 0.35 | -2.27 – 2.97 | | | | 0.27 | | 79.4 | | .791 |  |  |  | | | |  | |  | |  | |
|  | *OCD vs. SAD* | 0.91 | -1.69 – 3.51 | | | | 0.70 | | 78.26 | | .487 |  |  |  | | | |  | |  | |  | |
| **Group/OCI-R ×Feedback Valence** | |  |  | | | |  | |  | |  |  | **-0.04** | **-0.07 – -0.00** | | | | **-2.03** | | **71.2** | | **.046** | |
|  | ***OCD vs. HC*** | **1.28** | **0.08 – 2.48** | | | | **2.13** | | **69.64** | | **.037** |  |  |  | | | |  | |  | |  | |
|  | ***OCD vs. SAD*** | **1.34** | **0.14 – 2.53** | | | | **2.23** | | **69.05** | | **.029** |  |  |  | | | |  | |  | |  | |
| **Agency × Feedback Valence** | | **2.32** | **1.45 – 3.19** | | | | **5.35** | | **64.8** | | **<.001** |  | **2.32** | **1.46 – 3.19** | | | | **5.34** | | **66.38** | | **<.001** | |
| Group/OCI-R × SPE | |  |  | | | |  | |  | |  |  | 0.03 | -0.01 – 0.08 | | | | 1.37 | | 65.71 | | .174 | |
|  | *OCD vs. HC* | -0.45 | -1.90 – 1.00 | | | | -0.62 | | 58.67 | | .538 |  |  |  | | | |  | |  | |  | |
|  | *OCD vs. SAD* | -0.16 | -1.58 – 1.27 | | | | -0.22 | | 54.42 | | .825 |  |  |  | | | |  | |  | |  | |
| Agency × SPE | | -0.43 | -1.75 – 0.89 | | | | -0.65 | | 62.92 | | .516 |  | -0.47 | -1.78 – 0.84 | | | | -0.71 | | 64.83 | | .479 | |
| **Feedback Valence × SPE** | | **-3.73** | **-6.29 – -1.17** | | | | **-2.91** | | **67.29** | | **.005** |  | **-3.85** | **-6.43 – -1.27** | | | | **-2.98** | | **68.74** | | **.004** | |
| Group/OCI-R × Agency × Feedback Valence | |  |  | | | |  | |  | |  |  | 0.03 | -0.03 – 0.10 | | | | 1.04 | | 66.76 | | .304 | |
|  | *OCD vs. HC* | -1.69 | -3.82 – 0.44 | | | | -1.59 | | 65.32 | | .118 |  |  |  | | | |  | |  | |  | |
|  | *OCD vs. SAD* | -1.35 | -3.46 – 0.77 | | | | -1.27 | | 63.92 | | .208 |  |  |  | | | |  | |  | |  | |
| Group/OCI-R × Agency × SPE | |  |  | | | |  | |  | |  |  | -0.04 | -0.14 – 0.06 | | | | -0.88 | | 72.24 | | .380 | |
|  | *OCD vs. HC* | -1.64 | -4.90 – 1.62 | | | | -1.01 | | 64.54 | | .318 |  |  |  | | | |  | |  | |  | |
|  | *OCD vs. SAD* | -0.87 | -4.09 – 2.34 | | | | -0.55 | | 60.85 | | .588 |  |  |  | | | |  | |  | |  | |
| Group/OCI-R × Feedback Valence × SPE | |  |  | | | |  | |  | |  |  | -0.08 | -0.27 – 0.12 | | | | -0.78 | | 76.13 | | .436 | |
|  | *OCD vs. HC* | -3.42 | -9.72 – 2.88 | | | | -1.08 | | 68.2 | | .283 |  |  |  | | | |  | |  | |  | |
|  | *OCD vs. SAD* | -1.55 | -7.81 – 4.71 | | | | -0.49 | | 66.34 | | .623 |  |  |  | | | |  | |  | |  | |
| **Agency × Feedback Valence × SPE** | | **7.52** | **2.73 – 12.31** | | | | **3.13** | | **67.22** | | **.003** |  | **7.7** | **2.89 – 12.52** | | | | **3.19** | | **70.18** | | **.002** | |
| Group/OCI-R × Agency × Feedback Valence × SPE | |  |  | | | |  | |  | |  |  | -0.01 | -0.38 – 0.36 | | | | -0.06 | | 78.5 | | .956 | |
|  | *OCD vs. HC* | 3.94 | -7.84 – 15.73 | | | | 0.67 | | 68.19 | | .507 |  |  |  | | | |  | |  | |  | |
|  | *OCD vs. SAD* | -4.61 | -16.32 – 7.11 | | | | -0.79 | | 66.23 | | .435 |  |  |  | | | |  | |  | |  | |
|  |  |  |  | | | |  | |  | |  |  |  |  | | | |  | |  | |  | |
| *Random Effects* | |  | | | | | | | | | |  |  | | | | | | | | | | |
|  | | Variance | | *SD* | | Correlation | | | | | |  | Variance | | *SD* | | Correlation | | | | | | |
| Participant (Intercept) | | 19.32 | | 4.40 | |  | | | | | |  | 20.02 | | 4.48 | |  | | | | | | |
| Participant (Agency) | | 22.07 | | 4.70 | | -0.20 | | | | | |  | 21.83 | | 4.67 | | -0.21 | | | | | | |
| Participant (Feedback Valence) | | 4.21 | | 2.05 | | -0.03 0.10 | | | | | |  | 4.26 | | 2.07 | | -0.06 0.13 | | | | | | |
| Participant (SPE) | | 5.15 | | 2.27 | | 0.20 -0.20 -0.19 | | | | | |  | 4.81 | | 2.19 | | 0.20 -0.20 -0.14 | | | | | | |
| Participant (Agency × Feedback Valence) | | 12.67 | | 3.56 | | 0.00 -0.20 -0.46 0.04 | | | | | |  | 12.74 | | 3.57 | | 0.03 -0.21 -0.48 0.02 | | | | | | |
| Participant (Agency × SPE) | | 28.15 | | 5.31 | | -0.05 0.09 0.20 -0.41 -0.54 | | | | | |  | 27.81 | | 5.27 | | 0.01 0.10 0.15 -0.39 -0.48 | | | | | | |
| Participant (Feedback Valence × SPE) | | 120.27 | | 10.97 | | -0.31 0.39 0.19 -0.55 -0.19 0.43 | | | | | |  | 122.16 | | 11.05 | | -0.26 0.38 0.13 -0.54 -0.15 0.44 | | | | | | |
| Participant (Agency × Feedback Valence × SPE) | | 417.06 | | 20.42 | | 0.00 -0.59 0.01 0.34 -0.03 -0.26 -0.49 | | | | | |  | 421.87 | | 20.54 | | -0.01 -0.58 0.01 0.32 -0.04 -0.28 -0.50 | | | | | | |
| Electrode (Intercept) | | 1.07 | | 1.03 | |  | | |  | |  |  | 1.07 | | 1.03 | |  | | |  | |  | |
| Residual | | 100.07 | | 10.00 | |  | | |  | |  |  | 100.07 | | 10.00 | |  | | |  | |  | |
|  | |  |  | | | |  | |  | |  |  |  |  | | | |  | |  | |  | |
| *Goodness of fit* | |  | | | | | | | | | |  |  | | | | | | | | | | |
|  | | AIC | | | BIC | | | LL | | Deviance | |  | AIC | | | BIC | | | LL | | Deviance | | |
|  | | 1386376 | | | 1387004 | | | -693125.8 | | 1386252 | |  | 1386449 | | | 1386996 | | | -693170.4 | | 1386341 | | |
|  | | Marginal R^2^ | | | | | | Conditional R^2^ | | | |  | Marginal R^2^ | | | | | | Conditional R^2^ | | | | |
|  | | 0.055 | | | | | | 0.280 | | | |  | 0.043 | | | | | | 0.278 | | | | |
| *Note:* Abbreviations: HC = healthy controls; OCD = obsessive-compulsive disorder; SAD = social anxiety disorder; CI = confidence interval; SD = standard deviation; AIC = Akaike information criterion; BIC = Bayesian information criterion; LL = log-likelihood. Significant effects are highlighted in bold. | | | | | | | | | | | | | | | | | | | | | | | |

# Table S10: LMM results for the ERN/CRN (model-based)

| *Inferential Statistics of Model-Based ERN/CRN Linear Mixed-Effects Model Analyses* | | | | | | | | |
| --- | --- | --- | --- | --- | --- | --- | --- | --- |
| *Model Formula* | | ERN/CRN ~ group * ΔQ * learning status +  (1 + ΔQ * learning status \| participant) +  (1 \| electrode) | | | | | | |
|  |  |  | | | | | | |
| *Sampling Units* | |  | | | | | | |
|  | | *N* observations = 148590 | | | | | | |
|  | | *N* participants = 76 (26 OCD, 26 HC, 24 SAD) | | | | | | |
|  | | *N* electrodes = 5 (Fz, FCz, FC1, FC2, Cz) | | | | | | |
|  | |  | | | | | | |
| *Fixed Effects* | |  | | | | | | |
|  | | *b* | 95% CI | | *t* | *df* | | *p* |
| **Intercept** | | **-0.63** | **-0.96 – -0.30** | | **-3.77** | **65.01** | | **<.001** |
| Group | |  |  | |  |  | |  |
|  | *OCD vs. HC* | 0.42 | -0.34 – 1.17 | | 1.10 | 70.96 | | .274 |
|  | *OCD vs. SAD* | 0.43 | -0.34 – 1.20 | | 1.11 | 70.21 | | .271 |
| **ΔQ** | | **0.63** | **0.05 – 1.22** | | **2.22** | **29.13** | | **.034** |
| Learning Status | | -0.07 | -0.32 – 0.19 | | -0.52 | 56.14 | | .602 |
| Group × ΔQ | |  |  | |  |  | |  |
|  | *OCD vs. HC* | -0.44 | -1.84 – 0.97 | | -0.64 | 29.10 | | .530 |
|  | *OCD vs. SAD* | -0.65 | -2.10 – 0.80 | | -0.92 | 28.87 | | .368 |
| Group ×Learning Status | |  |  | |  |  | |  |
|  | *OCD vs. HC* | 0.43 | -0.19 – 1.06 | | 1.39 | 57.43 | | .171 |
|  | *OCD vs. SAD* | 0.36 | -0.27 – 0.99 | | 1.15 | 54.37 | | .255 |
| ΔQ × Learning Status | | 0.04 | -0.82 – 0.91 | | 0.10 | 49.18 | | .919 |
| Group × ΔQ × Learning Status | |  |  | |  |  | |  |
|  | *OCD vs. HC* | 0.02 | -2.07 – 2.11 | | 0.02 | 49.71 | | .984 |
|  | *OCD vs. SAD* | -1.05 | -3.20 – 1.10 | | -0.98 | 48.18 | | .332 |
|  |  |  |  | |  |  | |  |
| *Random Effects* | |  |  | |  |  | |  |
|  | | Variance | *SD* | Correlation | | | | |
| Participant (Intercept) | | 1.78 | 1.33 |  | | | | |
| Participant (ΔQ) | | 5.16 | 2.27 | -0.17 | | | | |
| Participant (Learning Status) | | 0.91 | 0.96 | 0.20 0.02 | | | | |
| Participant (ΔQ × Learning Status) | | 10.87 | 3.30 | 0.00 0.20 -0.41 | | | | |
| Electrode (Intercept) | | 0.02 | 0.13 |  | |  | |  |
| Residual | | 50.41 | 7.10 |  | |  | |  |
|  | | | | | | | | |
| *Goodness of fit* | |  | | | | | | |
|  | | AIC | BIC | | LL | | Deviance | |
|  | | 1004748 | 1004986 | | -502350.2 | | 1004700 | |
|  | | Marginal R^2^ | | | Conditional R^2^ | | | |
|  | | 0.002 | | | 0.068 | | | |
| *Note:* Abbreviations: HC = healthy controls; OCD = obsessive-compulsive disorder; SAD = social anxiety disorder; CI = confidence interval; SD = standard deviation; AIC = Akaike information criterion; BIC = Bayesian information criterion; LL = log-likelihood. Significant effects are highlighted in bold. | | | | | | | | |

# Table S11: LMM results for the P3 (model-free)

| *Inferential Statistics of Model-Free P3 Linear Mixed-Effects Model Analyses* | | | | | | | | | | | | | | | | | | | | |
| --- | --- | --- | --- | --- | --- | --- | --- | --- | --- | --- | --- | --- | --- | --- | --- | --- | --- | --- | --- | --- |
|  | | *1. Categorical Model* | | | | | | | | |  | *2. Transdiagnostic-Dimensional Model* | | | | | | | | |
| *Model Formula* | | P3 ~ group * agency * feedback valence +  (1 + agency * feedback valence \| participant) +  (1 \| electrode) | | | | | | | | |  | P3 ~ OCI-R * agency * feedback valence + (1 + agency * feedback valence \| participant) +  (1 \| electrode) | | | | | | | | |
|  |  |  | | | | | | | | |  |  | | | | | | | | |
| *Sampling Units* | |  | | | | | | | | |  |  | | | | | | | | |
|  | | *N* observations = 185974 | | | | | | | | |  | *N* observations = 147052 | | | | | | | | |
|  | | *N* participants = 81 (27 OCD, 27 HC, 27 SAD) | | | | | | | | |  | *N* participants = 81 (27 OCD, 27 HC, 29 SAD) | | | | | | | | |
|  | | *N* electrodes = 4 (Cz, CP1, CP2, Pz) | | | | | | | | |  | *N* electrodes = 4 (Cz, CP1, CP2, Pz) | | | | | | | | |
|  | |  | | | | | | | | |  |  | | | | | | | | |
| *Fixed Effects* | |  | | | | | | | | |  |  | | | | | | | | |
|  | | *b* | 95% CI | | | | *t* | *df* | | *p* |  | *b* | 95% CI | | | | | *t* | *df* | *p* |
| **Intercept** | | **11.05** | **9.92 – 12.18** | | | | **19.47** | **80.22** | | **<.001** |  | **11.05** | **9.91 – 12.20** | | | | | **19.20** | **81.49** | **<.001** |
| Group/OCI-R | |  |  | | | |  |  | |  |  | 0.03 | -0.05 – 0.12 | | | | | 0.81 | 80.48 | .419 |
|  | ***OCD vs. HC*** | **-2.72** | **-5.42 – -0.01** | | | | **-2.00** | **78.25** | | **.049** |  |  |  | | | | |  |  |  |
|  | *OCD vs. SAD* | -1.69 | -4.39 – 1.01 | | | | -1.25 | 77.59 | | .216 |  |  |  | | | | |  |  |  |
| **Agency** | | **-6.74** | **-7.86 – -5.62** | | | | **-11.99** | **77.22** | | **<.001** |  | **-6.73** | **-7.85 – -5.62** | | | | | **-12.01** | **78.75** | **<.001** |
| Feedback Valence | | 0.37 | -0.10 – 0.83 | | | | 1.58 | 78.32 | | .118 |  | 0.37 | -0.09 – 0.83 | | | | | 1.60 | 78.18 | .114 |
| Group/OCI-R × Agency | |  |  | | | |  |  | |  |  | 0.02 | -0.07 – 0.10 | | | | | 0.37 | 81.65 | .716 |
|  | *OCD vs. HC* | 0.95 | -1.80 – 3.71 | | | | 0.69 | 77.94 | | .494 |  |  |  | | | | |  |  |  |
|  | *OCD vs. SAD* | -0.09 | -2.82 – 2.64 | | | | -0.06 | 76.47 | | .950 |  |  |  | | | | |  |  |  |
| Group/OCI-R ×Feedback Valence | |  |  | | | |  |  | |  |  | -0.02 | -0.05 – 0.01 | | | | | -1.12 | 80.60 | .268 |
|  | *OCD vs. HC* | 0.48 | -0.66 – 1.62 | | | | 0.84 | 78.69 | | .405 |  |  |  | | | | |  |  |  |
|  | *OCD vs. SAD* | 0.29 | -0.84 – 1.43 | | | | 0.51 | 77.88 | | .609 |  |  |  | | | | |  |  |  |
| **Agency × Feedback Valence** | | **-0.75** | **-1.44 – -0.05** | | | | **-2.12** | **78.61** | | **.037** |  | **-0.74** | **-1.44 – -0.04** | | | | | **-2.12** | **79.43** | **.038** |
| **Group/OCI-R × Agency × Feedback Valence** | |  |  | | | |  |  | |  |  | **0.06** | **0.01 – 0.11** | | | | | **2.32** | **81.55** | **.023** |
|  | ***OCD vs. HC*** | **-2.02** | **-3.74 – -0.30** | | | | **-2.34** | **79.19** | | **.022** |  |  |  | | | | |  |  |  |
|  | ***OCD vs. SAD*** | **-1.80** | **-3.51 – -0.09** | | | | **-2.10** | **77.90** | | **.039** |  |  |  | | | | |  |  |  |
|  | | | | | | | | | | |  |  | | | | | | | | |
| *Random Effects* | |  | | | | | | | | |  |  | | | | | | | | |
|  | | Variance | | SD | Correlation | | | | | |  | Variance | | SD | | Correlation | | | | |
| Participant (Intercept) | | 24.71 | | 4.97 |  | | | | | |  | 25.48 | | 5.05 | |  | | | | |
| Participant (Agency) | | 24.85 | | 4.98 | -0.44 | | | | | |  | 24.71 | | 4.97 | | -0.45 | | | | |
| Participant (Feedback Valence) | | 4.09 | | 2.02 | 0.26 -0.10 | | | | | |  | 4.01 | | 2.00 | | 0.24 -0.08 | | | | |
| Participant (Agency × Feedback Valence) | | 8.75 | | 2.96 | 0.09 0.04 -0.67 | | | | | |  | 8.76 | | 2.96 | | 0.12 0.01 -0.66 | | | | |
| Electrode (Intercept) | | 0.06 | | 0.24 |  | | |  | |  |  | 0.06 | | 0.24 | |  | | |  |  |
| Residual | | 126.48 | | 11.25 |  | | |  | |  |  | 126.48 | | 11.25 | |  | | |  |  |
|  | | | | | | | | | | |  |  | | | | | | | | |
| *Goodness of fit* | |  |  | | | |  |  | |  |  |  |  | | | | |  |  |  |
|  | | AIC | | BIC | | LL | | | Deviance | |  | AIC | | | BIC | | LL | | Deviance | |
|  | | 1130246 | | 1130484 | | -565099.10 | | | 1130198 | |  | 1130276 | | | 1130474 | | -565117.9 | | 1130236 | |
|  | | Marginal R^2^ | | | | | Conditional R^2^ | | | |  | Marginal R^2^ | | | | | | Conditional R^2^ | | |
|  | | 0.074 | | | | | 0.263 |  | |  |  | 0.067 | | | | | | 0.261 | | |
| *Note:* Abbreviations: HC = healthy controls; OCD = obsessive-compulsive disorder; SAD = social anxiety disorder; CI = confidence interval; SD = standard deviation; AIC = Akaike information criterion; BIC = Bayesian information criterion; LL = log-likelihood. Significant effects are highlighted in bold. | | | | | | | | | | | | | | | | | | | | |

# Table S12: LMM results for the P3 (model-based)

| *Inferential Statistics of Model-Based P3 Linear Mixed-Effects Model Analyses* | | | | | | | | | | | | | | | | | | | | |
| --- | --- | --- | --- | --- | --- | --- | --- | --- | --- | --- | --- | --- | --- | --- | --- | --- | --- | --- | --- | --- |
|  | | *1. Categorical Model* | | | | | | | | |  | *2. Transdiagnostic-Dimensional Model^a^* | | | | | | | | |
| *Model Formula* | | P3 ~ group * agency * feedback valence * SPE +  (1 + agency * feedback valence * SPE \| participant) + (1 \| electrode) | | | | | | | | |  | P3 ~ OCI-R * agency * feedback valence + (1 + agency * feedback valence * SPE \| participant) + (1 \| electrode) | | | | | | | | |
|  |  |  | | | | | | | | |  |  | | | | | | | | |
| *Sampling Units* | |  | | | | | | | | |  |  | | | | | | | | |
|  | | *N* observations = 147052 | | | | | | | | |  | *N* observations = 147052 | | | | | | | | |
|  | | *N* participants = 81 (27 OCD, 27 HC, 27 SAD) | | | | | | | | |  | *N* participants = 81 (27 OCD, 27 HC, 29 SAD) | | | | | | | | |
|  | | *N* electrodes = 4 (Cz, CP1, CP2, Pz) | | | | | | | | |  | *N* electrodes = 4 (Cz, CP1, CP2, Pz) | | | | | | | | |
|  | |  | | | | | | | | |  |  | | | | | | | | |
| *Fixed Effects* | |  | | | | | | | | |  |  | | | | | | | | |
|  | | *b* | 95% CI | | | | *t* | *df* | | *p* |  | *b* | 95% CI | | | | *t* | *df* | | *p* |
| **Intercept** | | **11.96** | **10.82 – 13.10** | | | | **20.87** | **80.56** | | **<.001** |  | **11.97** | **10.82 – 13.13** | | | | **20.66** | **81.69** | | **<.001** |
| Group/OCI-R | |  |  | | | |  |  | |  |  | 0.03 | -0.06 – 0.11 | | | | 0.65 | 80.63 | | .517 |
|  | *OCD vs. HC* | -2.41 | -5.14 – 0.33 | | | | -1.75 | 78.55 | | .084 |  |  |  | | | |  |  | |  |
|  | *OCD vs. SAD* | -1.45 | -4.17 – 1.28 | | | | -1.06 | 77.89 | | .294 |  |  |  | | | |  |  | |  |
| **Agency** | | **-7.42** | **-8.67 – -6.18** | | | | **-11.87** | **77.38** | | **<.001** |  | **-7.40** | **-8.64 – -6.17** | | | | **-11.94** | **78.51** | | **<.001** |
| Feedback Valence | | -0.47 | -0.99 – 0.05 | | | | -1.79 | 74.97 | | .077 |  | -0.46 | -0.98 – 0.05 | | | | -1.78 | 74.91 | | .079 |
| **SPE** | | **1.48** | **0.85 – 2.11** | | | | **4.69** | **55.02** | | **<.001** |  | **1.55** | **0.92 – 2.19** | | | | **4.94** | **56.5** | | **<.001** |
| Group/OCI-R × Agency | |  |  | | | |  |  | |  |  | 0.02 | -0.07 – 0.11 | | | | 0.41 | 80.58 | | .683 |
|  | *OCD vs. HC* | -0.39 | -3.45 – 2.67 | | | | -0.25 | 77.97 | | .800 |  |  |  | | | |  |  | |  |
|  | *OCD vs. SAD* | -0.57 | -3.61 – 2.46 | | | | -0.38 | 76.77 | | .708 |  |  |  | | | |  |  | |  |
| Group/OCI-R ×Feedback Valence | |  |  | | | |  |  | |  |  | -0.02 | -0.06 – 0.02 | | | | -1.15 | 74.7 | | .254 |
|  | *OCD vs. HC* | 0.50 | -0.77 – 1.77 | | | | 0.78 | 75.08 | | .437 |  |  |  | | | |  |  | |  |
|  | *OCD vs. SAD* | -0.27 | -1.55 – 1.00 | | | | -0.43 | 74.23 | | .669 |  |  |  | | | |  |  | |  |
| Agency × Feedback Valence | | 0.38 | -0.59 – 1.36 | | | | 0.78 | 71.85 | | .435 |  | 0.39 | -0.57 – 1.35 | | | | 0.81 | 71.68 | | .418 |
| Group/OCI-R × SPE | |  |  | | | |  |  | |  |  | 0.03 | -0.01 – 0.08 | | | | 1.40 | 62.46 | | .167 |
|  | *OCD vs. HC* | -0.35 | -1.91 – 1.22 | | | | -0.44 | 56.88 | | .658 |  |  |  | | | |  |  | |  |
|  | *OCD vs. SAD* | 1.31 | -0.22 – 2.85 | | | | 1.72 | 52.38 | | .092 |  |  |  | | | |  |  | |  |
| **Agency × SPE** | | **-1.32** | **-2.62 – -0.03** | | | | **-2.04** | **63.48** | | **.045** |  | -1.29 | -2.59 – 0.01 | | | | -1.98 | 64.34 | | .052 |
| **Feedback Valence × SPE** | | **-7.91** | **-11.20 – -4.62** | | | | **-4.82** | **59.39** | | **<.001** |  | **-7.96** | **-11.28 – -4.64** | | | | **-4.79** | **60.58** | | **<.001** |
| Group/OCI-R × Agency × Feedback Valence | |  |  | | | |  |  | |  |  | 0.05 | -0.02 – 0.12 | | | | 1.55 | 71.37 | | .127 |
|  | *OCD vs. HC* | -1.76 | -4.15 – 0.64 | | | | -1.46 | 72.26 | | .148 |  |  |  | | | |  |  | |  |
|  | *OCD vs. SAD* | -0.65 | -3.04 – 1.73 | | | | -0.55 | 70.77 | | .586 |  |  |  | | | |  |  | |  |
| Group/OCI-R × Agency × SPE | |  |  | | | |  |  | |  |  | -0.02 | -0.12 – 0.07 | | | | -0.50 | 71.55 | | .621 |
|  | *OCD vs. HC* | -1.33 | -4.53 – 1.88 | | | | -0.83 | 65.51 | | .411 |  |  |  | | | |  |  | |  |
|  | *OCD vs. SAD* | -1.76 | -4.91 – 1.38 | | | | -1.12 | 60.63 | | .267 |  |  |  | | | |  |  | |  |
| Group/OCI-R × Feedback Valence × SPE | |  |  | | | |  |  | |  |  | -0.04 | -0.30 – 0.21 | | | | -0.34 | 66.77 | | .736 |
|  | *OCD vs. HC* | -3.79 | -11.89 – 4.31 | | | | -0.94 | 60.2 | | .353 |  |  |  | | | |  |  | |  |
|  | *OCD vs. SAD* | -1 | -9.06 – 7.07 | | | | -0.25 | 58.37 | | .805 |  |  |  | | | |  |  | |  |
| **Agency × Feedback Valence × SPE** | | **9.86** | **3.80 – 15.91** | | | | **3.26** | **57.75** | | **.002** |  | **10.26** | **4.28 – 16.24** | | | | **3.43** | **60.42** | | **.001** |
| Group/OCI-R × Agency × Feedback Valence × SPE | |  |  | | | |  |  | |  |  | 0.4 | -0.06 – 0.86 | | | | 1.73 | 67.53 | | .089 |
|  | *OCD vs. HC* | -1.59 | -16.51 – 13.33 | | | | -0.21 | 58.65 | | .832 |  |  |  | | | |  |  | |  |
|  | *OCD vs. SAD* | -3.64 | -18.48 – 11.20 | | | | -0.49 | 56.51 | | .625 |  |  |  | | | |  |  | |  |
|  |  |  |  | | | |  |  | |  |  |  |  | | | |  |  | |  |
| *Random Effects* | |  | | | | | | | | |  |  | | | | | | | | |
|  | | Variance | | *SD* | | Correlation | | | | |  | Variance | | *SD* | | Correlation | | | | |
| Participant (Intercept) | | 24.96 | | 5.00 | |  | | | | |  | 25.58 | | 5.06 | |  | | | | |
| Participant (Agency) | | 29.9 | | 5.47 | | -0.37 | | | | |  | 29.38 | | 5.42 | | -0.36 | | | | |
| Participant (Feedback Valence) | | 4.63 | | 2.15 | | 0.18 0.08 | | | | |  | 4.53 | | 2.14 | | 0.18 0.09 | | | | |
| Participant (SPE) | | 5.69 | | 2.39 | | 0.23 -0.20 -0.08 | | | | |  | 5.68 | | 2.40 | | 0.19 -0.20 -0.10 | | | | |
| Participant (Agency × Feedback Valence) | | 15.91 | | 3.99 | | 0.09 -0.28 -0.63 0.18 | | | | |  | 15.24 | | 3.88 | | 0.11 -0.28 -0.62 0.15 | | | | |
| Participant (Agency × SPE) | | 24.88 | | 4.99 | | -0.20 0.36 0.36 -0.55 -0.62 | | | | |  | 24.81 | | 4.97 | | -0.14 0.35 0.38 -0.54 -0.61 | | | | |
| Participant (Feedback Valence × SPE) | | 197.21 | | 14.04 | | -0.14 0.16 0.14 -0.39 0.03 0.26 | | | | |  | 201.58 | | 14.13 | | -0.11 0.16 0.13 -0.37 0.05 0.27 | | | | |
| Participant (Agency × Feedback Valence × SPE) | | 655.89 | | 25.61 | | -0.06 -0.23 -0.18 0.55 0.23 -0.36 -0.49 | | | | |  | 636.30 | | 25.24 | | -0.06 -0.24 -0.15 0.50 0.19 -0.35 -0.51 | | | | |
| Electrode (Intercept) | | 0.06 | | 0.24 | |  | | | | |  | 0.06 | | 0.24 | |  | | | | |
| Residual | | 124.22 | | 11.15 | |  | | | | |  | 124.22 | | 11.15 | |  | | | | |
|  | |  |  | | | |  |  | |  |  |  |  | | | |  |  | |  |
| *Goodness of fit* | |  | | | | | | | | |  |  | | | | | | | | |
|  | | AIC | | | BIC | | LL | | Deviance | |  | AIC | | | BIC | | LL | | Deviance | |
|  | | 1128297 | | | 1128910 | | -564086.4 | | 1128173 | |  | 1128336 | | | 1128870 | | -564113.9 | | 1128228 | |
|  | | Marginal R2 | | | | | Conditional R2 | | | |  | Marginal R2 | | | | | Conditional R2 | | | |
|  | | 0.075 | | | | | 0.317 | | | |  | 0.069 | | | | | 0.316 | | | |
| *Note:* Abbreviations: HC = healthy controls; OCD = obsessive-compulsive disorder; SAD = social anxiety disorder; CI = confidence interval; SD = standard deviation; AIC = Akaike information criterion; BIC = Bayesian information criterion; LL = log-likelihood. Significant effects are highlighted in bold.  ^a^ Model failed to converge with max\|grad\| = 0.00228523 (tol = 0.002, component 1) | | | | | | | | | | | | | | | | | | | | |

# Table S13: GLMM results on brain-behavior relationships

| *Inferential Statistics of Generalized Linear Mixed-Effects Model (GLMM) Analyses on Brain Behavior Relationships* | | | | | | | | | | | | | |
| --- | --- | --- | --- | --- | --- | --- | --- | --- | --- | --- | --- | --- | --- |
|  | | *1. FRN Model* | | | | | |  | *2. P3 Model* | | | | |
| *Model Formula* | | choice shift ~ group * previous feedback authenticity * block * previous FRN +  (1 + previous authenticity * block \| participant) | | | | | |  | choice shift ~ group * previous feedback authenticity * block * previous P3 +  (1 + previous authenticity * block \| participant) | | | | |
|  |  |  | | | | | |  |  | | | | |
| *Sampling Units* | |  | | | | | |  |  | | | | |
|  | | *N* observations = 7343 | | | | | |  | *N* observations = 7343 | | | | |
|  | | *N participants* = 78 (27 OCD, 25 HC, 26 SAD) | | | | | |  | *N participants* = 78 (27 OCD, 25 HC, 26 SAD) | | | | |
|  | |  | | | | | |  |  | | | | |
| *Fixed Effects* | |  | | | | | |  |  | | | | |
|  | | *b* | 95% CI | | *z* | | *p* |  | *b* | 95% CI | *z* | *p* | |
| **Intercept** | | **-0.31** | **-0.42 – -0.20** | | **-5.64** | | **<.001** |  | **-0.32** | **-0.43 – -0.21** | **-5.71** | **<.001** | |
| Group/OCI-R | |  |  | |  | |  |  |  |  |  |  | |
|  | ***OCD vs. HC*** | **-0.26** | **-0.52 – -0.00** | | **-1.97** | | **.049** |  | -0.26 | -0.52 – 0.00 | -1.94 | .052 | |
|  | *OCD vs. SAD* | 0.18 | -0.07 – 0.44 | | 1.44 | | .151 |  | 0.18 | -0.07 – 0.43 | 1.4 | .162 | |
| **Previous Feedback Authenticity** | | **-1.26** | **-1.50 – -1.02** | | **-10.29** | | **<.001** |  | **-1.26** | **-1.50 – -1.02** | **-10.32** | **<.001** | |
| **Block** | | **-0.10** | **-0.16 – -0.03** | | **-2.8** | | **.005** |  | **-0.09** | **-0.16 – -0.02** | **-2.67** | **.007** | |
| **Previous FRN/P3** | | **0.07** | **0.01 – 0.12** | | **2.5** | | **.013** |  | **0.06** | **0.01 – 0.10** | **2.35** | **.019** | |
| Group × Previous Feedback Authenticity | |  |  | |  | |  |  |  |  |  |  | |
|  | *OCD vs. HC* | -0.33 | -0.91 – 0.25 | | -1.11 | | .268 |  | -0.33 | -0.91 – 0.25 | -1.12 | .264 | |
|  | *OCD vs. SAD* | 0.05 | -0.51 – 0.61 | | 0.18 | | .855 |  | 0.03 | -0.53 – 0.59 | 0.1 | .917 | |
| Group × Block | |  |  | |  | |  |  |  |  |  |  | |
|  | *OCD vs. HC* | -0.04 | -0.20 – 0.11 | | -0.55 | | .581 |  | -0.04 | -0.20 – 0.11 | -0.55 | .580 | |
|  | *OCD vs. SAD* | 0.01 | -0.14 – 0.16 | | 0.16 | | .876 |  | 0.03 | -0.12 – 0.17 | 0.36 | .715 | |
| **Previous Feedback Authenticity × Block** | | **-0.20** | **-0.34 – -0.05** | | **-2.72** | | **.006** |  | **-0.19** | **-0.33 – -0.05** | **-2.65** | **.007** | |
| Group × Previous FRN/P3 | |  |  | |  | |  |  |  |  |  |  | |
|  | *OCD vs. HC* | -0.12 | -0.26 – 0.01 | | -1.81 | | .071 |  | -0.09 | -0.21 – 0.03 | -1.44 | .150 | |
|  | *OCD vs. SAD* | -0.06 | -0.18 – 0.07 | | -0.9 | | .368 |  | 0.04 | -0.07 – 0.14 | 0.66 | .510 | |
| Previous Feedback Authenticity × Previous FRN/P3 | | 0.06 | -0.04 – 0.17 | | 1.19 | | .235 |  | 0.06 | -0.04 – 0.15 | 1.19 | .235 | |
| Block × Previous FRN/P3 | | -0.03 | -0.08 – 0.02 | | -1.33 | | .186 |  | -0.02 | -0.06 – 0.03 | -0.72 | .476 | |
| Group × Previous Feedback Authenticity × Block | |  |  | |  | |  |  |  |  |  |  | |
|  | *OCD vs. HC* | -0.23 | -0.57 – 0.10 | | -1.37 | | .171 |  | -0.23 | -0.57 – 0.10 | -1.35 | .177 | |
|  | *OCD vs. SAD* | 0.07 | -0.25 – 0.39 | | 0.45 | | .656 |  | 0.09 | -0.24 – 0.41 | 0.52 | .600 | |
| Group × Previous Authenticity × Previous FRN/P3 | |  |  | |  | |  |  |  |  |  |  | |
|  | *OCD vs. HC* | 0.00 | -0.27 – 0.26 | | -0.02 | | .981 |  | -0.09 | -0.33 – 0.15 | -0.71 | .480 | |
|  | *OCD vs. SAD* | 0.05 | -0.21 – 0.30 | | 0.37 | | .713 |  | 0.00 | -0.22 – 0.22 | 0 | .996 | |
| Group × Block × Previous FRN/P3 | |  |  | |  | |  |  |  |  |  |  | |
|  | *OCD vs. HC* | 0.09 | -0.03 – 0.21 | | 1.49 | | .135 |  | 0.00 | -0.11 – 0.11 | 0.01 | .989 | |
|  | *OCD vs. SAD* | 0.03 | -0.08 – 0.15 | | 0.57 | | .571 |  | -0.03 | -0.12 – 0.07 | -0.54 | .591 | |
| Previous Feedback Authenticity × Block × Previous FRN/P3 | | 0.09 | -0.00 – 0.19 | | 1.94 | | .053 |  | 0.00 | -0.09 – 0.08 | -0.02 | .981 | |
| Group × Previous Feedback Authenticity × Block × Previous FRN/P3 | |  |  | |  | |  |  |  |  |  |  | |
|  | *OCD vs. HC* | -0.08 | -0.31 – 0.16 | | -0.62 | | .537 |  | -0.12 | -0.33 – 0.10 | -1.05 | .296 | |
|  | *OCD vs. SAD* | 0.02 | -0.21 – 0.25 | | 0.14 | | .886 |  | -0.16 | -0.35 – 0.03 | -1.62 | .105 | |
|  |  | | | | | | |  |  | | | | |
| *Random Effects* | |  | | | | | |  |  | | | | |
|  | | Variance | *SD* | | Correlation | | |  | Variance | *SD* | Correlation | | |
| Participant (Intercept) | | 0.16 | 0.40 | |  | | |  | 0.16 | 0.40 |  | | |
| Participant (Previous Feedback Authenticity) | | 0.86 | 0.93 | | 0.43 | | |  | 0.86 | 0.93 | 0.43 | | |
| Participant (Block) | | 0.03 | 0.17 | | 0.85 0.30 | | |  | 0.03 | 0.17 | 0.85 0.30 | | |
| Participant (Previous Feedback Authenticity × Block) | | 0.18 | 0.42 | | 0.33 0.61 -0.10 | | |  | 0.18 | 0.42 | 0.31 0.61 -0.11 | | |
|  | | | | | | | |  |  | | | | |
| *Goodness of fit* | |  | | | | | |  |  | | | | |
|  | | AIC | BIC | LL | | Deviance | |  | AIC | BIC | LL | | Deviance |
|  | | 9137.35 | 9372.001 | -4534.68 | | 9069.35 | |  | 9142.34 | 9376.99 | -4537.17 | | 9074.34 |
|  | | Marginal R^2^ | | | Conditional R^2^ | | |  | Marginal R^2^ | | Conditional R^2^ | | |
|  | | 0.116 | | | 0.226 | | |  | 0.115 | | 0.225 | | |
| *Note:* Abbreviations: HC = healthy controls; OCD = obsessive-compulsive disorder; SAD = social anxiety disorder; CI = confidence interval; SD = standard deviation; AIC = Akaike information criterion; BIC = Bayesian information criterion; LL = log-likelihood. Significant effects are highlighted in bold. | | | | | | | | | | | | | |

# Table S14a: Supplementary BDI-II analyses (choice accuracy during test trials)

| *Inferential Statistics of Generalized Linear Mixed-Effects Model (GLMM) Analyses on Choice Accuracy (Test Phase)* | | | | | |
| --- | --- | --- | --- | --- | --- |
| *Model Formula* | choice accuracy ~ BDI-II * agency * contingency * block + | | | | |
|  | (1 + agency * contingency * block \| participant) | | | | |
| *Sampling Units* |  | | | | |
|  | *N* observations = 19910 | | | | |
|  | *N* participants = 83 (27 OCD, 27 HC, 29 SAD) | | | | |
|  |  | | | | |
| *Fixed Effects* |  | | | | |
|  | *b* | 95% CI | *z* | | *p* |
| **Intercept** | **1.65** | **1.41 – 1.89** | **13.39** | | **<.001** |
| **BDI-II** | **-0.03** | **-0.05 – -0.01** | **-2.65** | | **.008** |
| Agency | 0.11 | -0.31 – 0.53 | 0.51 | | .609 |
| **Contingency** | **-0.71** | **-0.90 – -0.53** | **-7.52** | | **<.001** |
| **Block** | **0.24** | **0.15 – 0.33** | **5.14** | | **<.001** |
| BDI-II × Agency | 0.02 | -0.02 – 0.06 | 1.09 | | .276 |
| **BDI-II × Contingency** | **0.02** | **0.00 – 0.03** | **2.07** | | **.039** |
| Agency × Contingency | 0.24 | -0.14 – 0.62 | 1.26 | | .209 |
| BDI-II × Block | 0.00 | -0.01 – 0.01 | -0.68 | | .496 |
| Agency × Block | 0.00 | -0.20 – 0.19 | -0.04 | | .968 |
| Contingency × Block | -0.06 | -0.17 – 0.05 | -0.98 | | .326 |
| BDI-II × Agency × Contingency | 0.01 | -0.02 – 0.04 | 0.58 | | .565 |
| BDI-II × Agency × Block | 0.00 | -0.01 – 0.02 | 0.31 | | .758 |
| BDI-II × Contingency × Block | 0.00 | -0.01 – 0.01 | 0.04 | | .970 |
| Agency × Contingency × Block | 0.08 | -0.15 – 0.30 | 0.66 | | .506 |
| BDI-II × Agency × Contingency × Block | -0.02 | -0.04 – 0.00 | -1.63 | | .103 |
|  | | | | | |
| *Random Effects* |  | | | | |
|  | Variance | *SD* | Correlation | | |
| Participant (Intercept) | 1.17 | 1.08 |  | | |
| Participant (Agency) | 3.55 | 1.88 | 0.15 | | |
| Participant (Contingency) | 0.64 | 0.80 | -0.45 -0.29 | | |
| Participant (Block) | 0.12 | 0.35 | 0.06 0.11 -0.03 | | |
| Participant (Agency × Contingency) | 2.65 | 1.63 | 0.14 -0.04 0.14 -0.12 | | |
| Participant (Agency × Block) | 0.56 | 0.75 | -0.23 0.17 0.38 0.46 -0.04 | | |
| Participant (Contingency × Block) | 0.18 | 0.43 | 0.06 0.09 -0.17 -0.13 0.08 -0.43 | | |
| Participant (Agency × Contingency × Block) | 0.77 | 0.88 | -0.10 0.17 -0.19 -0.15 0.11 -0.09 0.03 | | |
|  | | | | | |
| *Goodness of fit* |  | | | | |
|  | AIC | BIC | LL | Deviance | |
|  | 18488.56 | 18899.30 | -9192.27 | 18384.56 | |
|  | Marginal R^2^ | | Conditional R^2^ | | |
|  | 0.078 | | 0.558 | | |

*Note:* Abbreviations: HC = healthy controls; OCD = obsessive-compulsive disorder; SAD = social anxiety disorder; CI = confidence interval; SD = standard deviation; AIC = Akaike information criterion; BIC = Bayesian information criterion; LL = log-likelihood. Significant effects are highlighted in bold.

# Table S14b: Supplementary BDI-II analyses (choice accuracy during learning trials)

| *Inferential Statistics from Generalized Linear Mixed-Effects Model Analyses on Learning Phase Performance* | | | | | |
| --- | --- | --- | --- | --- | --- |
| *Model Formula* | choice accuracy ~ BDI-II * contingency * block + | | | | |
|  | (1 + contingency * block \| participant) | | | | |
| *Sampling Units* |  | | | | |
|  | *N* observations = 19841 | | | | |
|  | *N* participants = 83 (27 OCD, 27 HC, 29 SAD) | | | | |
|  |  | | | | |
| *Fixed Effects* |  | | | | |
|  | *b* | 95% CI | *z* | | *p* |
| **Intercept** | **1.16** | **0.96 – 1.37** | **11.07** | | **<.001** |
| **BDI-II** | **-0.03** | **-0.04 – -0.01** | **-2.68** | | **.007** |
| **Contingency** | **-0.62** | **-0.78 – -0.46** | **-7.61** | | **<.001** |
| **Block** | **0.30** | **0.20 – 0.39** | **6.07** | | **<.001** |
| BDI-II × Contingency | 0.01 | -0.00 – 0.03 | 1.84 | | .066 |
| BDI-II × Block | -0.01 | -0.02 – 0.00 | -1.88 | | .060 |
| **Contingency × Block** | **-0.14** | **-0.23 – -0.04** | **-2.89** | | **.004** |
| BDI-II × Contingency × Block | 0.00 | -0.01 – 0.01 | 0.29 | | .769 |
|  | | | | | |
| *Random Effects* |  |  |  |  |  |
|  | Variance | *SD* | Correlation | | |
| Participant (Intercept) | 0.86 | 0.93 |  | | |
| Participant (Contingency) | 0.48 | 0.69 | -0.35 | | |
| Participant (Block) | 0.16 | 0.40 | 0.73 -0.22 | | |
| Participant (Contingency × Block) | 0.14 | 0.37 | -0.29 0.34 -0.34 | | |
|  | | | | | |
| *Goodness of fit* |  |  |  |  |  |
|  | AIC | BIC | LL | Deviance | |
|  | 21200.61 | 21342.73 | -10582.30 | 21164.61 | |
|  | Marginal R^2^ | | Conditional R^2^ | | |
|  | 0.092 | | 0.376 | | |

*Note:* Abbreviations: HC = healthy controls; OCD = obsessive-compulsive disorder; SAD = social anxiety disorder; CI = confidence interval; SD = standard deviation; AIC = Akaike information criterion; BIC = Bayesian information criterion; LL = log-likelihood. Significant effects are highlighted in bold.

# Table S15: Supplementary BDI-II analyses (win-stay/lose-shift behavior)

| *Inferential Statistics from Generalized Linear Mixed-Effects Model Analyses on Win-Stay/Lose-Shift Behavior* | | | | | | | | | | |
| --- | --- | --- | --- | --- | --- | --- | --- | --- | --- | --- |
| *Model Formula* | | choice shift ~ BDI-II * previous feedback valence * previous feedback authenticity * block +  (1 + previous feedback valence * previous authenticity * block \| participant) | | | | | | | | |
|  |  |  | | | | | | | | |
| *Sampling Units* | |  | | | | | | | | |
|  | | *N* observations = 18795 | | | | | | | | |
|  | | *N* participants = 83 (27 OCD, 27 HC, 29 SAD) | | | | | | | | |
|  | |  | | | | | | | | |
| *Fixed Effects* | |  | | | | | | | | |
|  | | *b* | 95% CI | | | | *z* | | *p* | |
| **Intercept** | | **-0.85** | **-1.00 – -0.71** | | | | **-11.30** | | **<.001** | |
| **BDI-II** | | **0.02** | **0.01 – 0.04** | | | | **3.36** | | **.001** | |
| **Previous Feedback Valence** | | **0.86** | **0.69 – 1.02** | | | | **10.35** | | **<.001** | |
| Previous Feedback Authenticity | | -0.05 | -0.13 – 0.03 | | | | -1.20 | | .231 | |
| **Block** | | **-0.13** | **-0.20 – -0.06** | | | | **-3.47** | | **.001** | |
| BDI-II × Previous Feedback Valence | | -0.01 | -0.02 – 0.01 | | | | -1.25 | | .212 | |
| BDI-II ×Previous Feedback Authenticity | | 0.00 | -0.01 – 0.01 | | | | -0.48 | | .628 | |
| **Previous Feedback Valence × Previous Feedback Authenticity** | | **-1.76** | **-1.92 – -1.60** | | | | **-21.68** | | **<.001** | |
| BDI-II × Block | | 0.00 | -0.00 – 0.01 | | | | 0.58 | | .563 | |
| Previous Feedback Valence × Block | | -0.04 | -0.12 – 0.05 | | | | -0.90 | | .368 | |
| Previous Feedback Authenticity × Block | | 0.05 | -0.02 – 0.12 | | | | 1.50 | | .133 | |
| **BDI-II × Previous Feedback Valence × Previous Feedback Authenticity** | | **0.03** | **0.02 – 0.05** | | | | **4.21** | | **<.001** | |
| BDI-II × Previous Feedback Valence × Block | | 0.00 | -0.00 – 0.01 | | | | 0.70 | | .481 | |
| BDI-II × Previous Feedback Authenticity × Block | | 0.00 | -0.01 – 0.00 | | | | -0.59 | | .556 | |
| **Previous Feedback Valence × Previous Feedback Authenticity × Block** | | **-0.22** | **-0.36 – -0.08** | | | | **-3.11** | | **.002** | |
| BDI-II × Previous Feedback Valence × Previous Feedback Authenticity × Block | | 0.01 | -0.00 – 0.02 | | | | 1.56 | | .119 | |
|  |  |  |  | | | |  |  | |  |
| *Random Effects* | |  |  | | | |  |  | |  |
|  | | Variance | *SD* | | Correlation | | | | | |
| Participant (Intercept) | | 0.43 | 0.66 | |  | | | | | |
| Participant (Previous Feedback Valence) | | 0.40 | 0.64 | | -0.68 | | | | | |
| Participant (Block) | | 0.08 | 0.28 | | 0.66 -0.39 | | | | | |
| Participant (Previous Feedback Valence × Block) | | 0.03 | 0.18 | | 0.25 -0.01 -0.38 | | | | | |
|  | |  |  | |  | | |  | |  |
| *Goodness of fit* | |  | | | | | | | | |
|  | | AIC | | BIC | | LL | | Deviance | | |
|  | | 19939.12 | | 20143 | | -9943.56 | | 19887.12 | | |
|  | | Marginal R^2^ | | | | Conditional R^2^ | | | | |
|  | | 0.137 | | | | 0.287 | | | | |

*Note:* Abbreviations: HC = healthy controls; OCD = obsessive-compulsive disorder; SAD = social anxiety disorder; CI = confidence interval; SD = standard deviation; AIC = Akaike information criterion; BIC = Bayesian information criterion; LL = log-likelihood. Significant effects are highlighted in bold.

# Table S16: Supplementary BDI-II analyses (model-free FRN analysis)

| *Inferential Statistics of Model-Free FRN Linear Mixed-Effects Model Analyses* | | | | | | | | |
| --- | --- | --- | --- | --- | --- | --- | --- | --- |
|  | |  | | | | | | |
| *Model Formula* | | FRN ~ BDI-II * agency * feedback valence (1 + agency * feedback valence \| participant) + (1 \| electrode) | | | | | | |
|  |  |  | | | | | | |
| *Sampling Units* | |  | | | | | | |
|  | | *N* observations = 185974 | | | | | | |
|  | | *N* participants = 81 (27 OCD, 27 HC, 27 SAD) | | | | | | |
|  | | *N* electrodes = 5 (Fz, FCz, FC1, FC2, Cz) | | | | | | |
|  | |  | | | | | | |
| *Fixed Effects* | |  | | | | | | |
|  | | *b* | 95% CI | | *t* | *df* | | *p* |
| **Intercept** | | **3.40** | **2.02 – 4.79** | | **5.25** | **14.73** | | **<.001** |
| **BDI-II** | | **0.11** | **0.02 – 0.20** | | **2.53** | **78.2** | | **.013** |
| **Agency** | | **-3.81** | **-4.65 – -2.98** | | **-9.10** | **76.25** | | **<.001** |
| **Feedback Valence** | | **-1.62** | **-2.09 – -1.15** | | **-6.92** | **78.66** | | **<.001** |
| BDI-II × Agency | | -0.04 | -0.12 – 0.04 | | -0.98 | 76.87 | | .330 |
| BDI-II ×Feedback Valence | | -0.04 | -0.08 – 0.01 | | -1.70 | 79.06 | | .093 |
| **Agency × Feedback Valence** | | **1.86** | **1.20 – 2.53** | | **5.59** | **78.38** | | **<.001** |
| BDI-II × Agency × Feedback Valence | | 0.05 | -0.01 – 0.12 | | 1.68 | 79.03 | | .097 |
|  |  |  |  | |  |  | |  |
| *Random Effects* | |  |  | |  |  | |  |
|  | | Variance | *SD* | Correlation | | | | |
| Participant (Intercept) | | 16.66 | 4.08 |  | | | | |
| Participant (Agency) | | 13.68 | 3.70 | -0.27 | | | | |
| Participant (Feedback Valence) | | 4.25 | 2.06 | 0.00 0.02 | | | | |
| Participant (Agency × Feedback Valence) | | 8.19 | 2.86 | -0.02 -0.16 -0.61 | | | | |
| Electrode (Intercept) | | 1.07 | 1.03 |  | |  | |  |
| Residual | | 101.51 | 10.08 |  | |  | |  |
|  | |  |  | |  |  | |  |
| *Goodness of fit* | |  |  | |  |  | |  |
|  | | AIC | BIC | | LL | | Deviance | |
|  | | 1388308 | 1388511 | | -694134.2 | | 1388268 | |
|  | | Marginal R^2^ | | | Conditional R^2^ | | | |
|  | | 0.048 | | | 0.223 | | | |

*Note:* Abbreviations: HC = healthy controls; OCD = obsessive-compulsive disorder; SAD = social anxiety disorder; CI = confidence interval; SD = standard deviation; AIC = Akaike information criterion; BIC = Bayesian information criterion; LL = log-likelihood. Significant effects are highlighted in bold.

# Table S17: Supplementary BDI-II analyses (model-free P3 analysis)

| *Inferential Statistics of Model-Free P3 Linear Mixed-Effects Model Analyses* | | | | | | | | |
| --- | --- | --- | --- | --- | --- | --- | --- | --- |
|  | | *1. Categorical Model* | | | | | | |
| *Model Formula* | | P3 ~ BDI-II * agency * feedback valence + (1 + agency * feedback valence \| participant) +  (1 \| electrode) | | | | | | |
|  |  |  | | | | | | |
| *Sampling Units* | |  | | | | | | |
|  | | *N* observations = 185974 | | | | | | |
|  | | *N* participants = 81 (27 OCD, 27 HC, 27 SAD) | | | | | | |
|  | | *N* electrodes = 4 (Cz, CP1, CP2, Pz) | | | | | | |
|  | |  | | | | | | |
| *Fixed Effects* | |  | | | | | | |
|  | | *b* | 95% CI | | *t* | *df* | | *p* |
| **Intercept** | | **11.05** | **9.93 – 12.17** | | **19.66** | **81.18** | | **<.001** |
| **BDI-II** | | **0.11** | **0.01 – 0.22** | | **2.16** | **79.28** | | **.034** |
| **Agency** | | **-6.75** | **-7.86 – -5.63** | | **-12.01** | **78.37** | | **<.001** |
| Feedback Valence | | 0.37 | -0.10 – 0.83 | | 1.58 | 79.32 | | .118 |
| BDI-II × Agency | | -0.01 | -0.12 – 0.10 | | -0.20 | 79.04 | | .845 |
| BDI-II ×Feedback Valence | | -0.01 | -0.05 – 0.03 | | -0.46 | 79.67 | | .648 |
| **Agency × Feedback Valence** | | **-0.75** | **-1.46 – -0.04** | | **-2.10** | **79.66** | | **.039** |
| BDI-II × Agency × Feedback Valence | | 0.06 | -0.01 – 0.13 | | 1.74 | 80.17 | | .085 |
|  |  |  |  | |  |  | |  |
| *Random Effects* | |  |  | |  |  | |  |
|  | | Variance | *SD* | Correlation | | | | |
| Participant (Intercept) | | 24.21 | 4.92 |  | | | | |
| Participant (Agency) | | 24.80 | 4.98 | -0.45 | | | | |
| Participant (Feedback Valence) | | 4.06 | 2.02 | 0.25 -0.09 | | | | |
| Participant (Agency × Feedback Valence) | | 9.07 | 3.01 | 0.10 0.03 -0.67 | | | | |
| Electrode (Intercept) | | 0.06 | 0.24 |  | |  | |  |
| Residual | | 126.48 | 11.25 |  | |  | |  |
|  | |  |  | |  |  | |  |
| *Goodness of fit* | |  |  | |  |  | |  |
|  | | AIC | BIC | | LL | | Deviance | |
|  | | 1130272 | 1130470 | | -565116.2 | | 1130232 | |
|  | | Marginal R^2^ | | | Conditional R^2^ | | | |
|  | | 0.074 | | | 0.261 | | | |

*Note:* Abbreviations: HC = healthy controls; OCD = obsessive-compulsive disorder; SAD = social anxiety disorder; CI = confidence interval; SD = standard deviation; AIC = Akaike information criterion; BIC = Bayesian information criterion; LL = log-likelihood. Significant effects are highlighted in bold.

# Table S18: Supplementary BDI-II analyses (model-based FRN analysis)

| *Inferential Statistics of Model-Based FRN Linear Mixed-Effects Model Analyses* | | | | | | | | |
| --- | --- | --- | --- | --- | --- | --- | --- | --- |
| *Model Formula* | | FRN ~ BDI-II * agency * feedback valence * SPE + (1 + agency * feedback valence * SPE \| participant) + (1 \| electrode) | | | | | | |
|  |  |  | | | | | | |
|  | |  | | | | | | |
| *Sampling Units* | |  | | | | | | |
|  | | *N* observations = 185974 | | | | | | |
|  | | *N* participants = 81 (27 OCD, 27 HC, 27 SAD) | | | | | | |
|  | | *N* electrodes = 5 (Fz, FCz, FC1, FC2, Cz) | | | | | | |
|  | |  | | | | | | |
| *Fixed Effects* | |  | | | | | | |
|  | | *b* | 95% CI | | *t* | *df* | | *p* |
| **Intercept** | | **3.88** | **2.45 – 5.31** | | **5.71** | **17.35** | | **<.001** |
| BDI-II | | 0.08 | -0.02 – 0.17 | | 1.63 | 78.67 | | .107 |
| **Agency** | | **-4.66** | **-5.72 – -3.60** | | **-8.75** | **79.13** | | **<.001** |
| **Feedback Valence** | | **-1.86** | **-2.36 – -1.36** | | **-7.45** | **70.6** | | **<.001** |
| SPE | | 0.44 | -0.15 – 1.02 | | 1.50 | 56.83 | | .139 |
| BDI-II × Agency | | 0.03 | -0.07 – 0.13 | | 0.54 | 79.34 | | .591 |
| BDI-II ×Feedback Valence | | -0.04 | -0.09 – 0.01 | | -1.70 | 70.16 | | .094 |
| **Agency × Feedback Valence** | | **2.32** | **1.45 – 3.19** | | **5.31** | **66.62** | | **<.001** |
| BDI-II × SPE | | 0.04 | -0.02 – 0.10 | | 1.33 | 61.16 | | .190 |
| Agency × SPE | | -0.44 | -1.76 – 0.88 | | -0.66 | 63.02 | | .511 |
| **Feedback Valence × SPE** | | **-3.65** | **-6.17 – -1.13** | | **-2.89** | **67.11** | | **.005** |
| BDI-II × Agency × Feedback Valence | | 0.02 | -0.06 – 0.10 | | 0.44 | 66.29 | | .664 |
| BDI-II × Agency × SPE | | 0.02 | -0.11 – 0.15 | | 0.32 | 66.92 | | .754 |
| BDI-II × Feedback Valence × SPE | | 0.13 | -0.12 – 0.37 | | 1.02 | 70.05 | | .313 |
| **Agency × Feedback Valence × SPE** | | **7.31** | **2.66 – 11.96** | | **3.14** | **67.99** | | **.003** |
| **BDI-II × Agency × Feedback Valence × SPE** | | **-0.46** | **-****0.92 – -0.01** | | **-2.04** | **71.42** | | **.045** |
|  |  |  |  | |  |  | |  |
| *Random Effects* | |  |  | |  |  | |  |
|  | | Variance | *SD* | Correlation | | | | |
| Participant (Intercept) | | 19.76 | 4.45 |  | | | | |
| Participant (Agency) | | 21.83 | 4.67 | -0.22 | | | | |
| Participant (Feedback Valence) | | 4.43 | 2.10 | -0.05 0.13 | | | | |
| Participant (SPE) | | 5.07 | 2.25 | 0.19 -0.21 -0.17 | | | | |
| Participant (Agency × Feedback Valence) | | 12.83 | 3.58 | 0.04 -0.21 -0.48 0.04 | | | | |
| Participant (Agency × SPE) | | 28.31 | 5.32 | -0.01 0.09 0.18 -0.42 -0.49 | | | | |
| Participant (Feedback Valence × SPE) | | 116.83 | 10.81 | -0.29 0.38 0.18 -0.58 -0.18 0.45 | | | | |
| Participant (Agency × Feedback Valence × SPE) | | 390.55 | 19.76 | 0.03 -0.60 -0.04 0.37 -0.03 -0.26 -0.47 | | | | |
| Electrode (Intercept) | | 1.07 | 1.03 |  | |  | |  |
| Residual | | 100.07 | 10.00 |  | |  | |  |
|  | |  |  | |  |  | |  |
| *Goodness of fit* | |  |  | |  |  | |  |
|  | | AIC | BIC | | LL | | Deviance | |
|  | | 1386438 | 1386985 | | -693165 | | 1386330 | |
|  | | Marginal R^2^ | | | Conditional R^2^ | | | |
|  | | 0.049 | | | 0.277 | | | |

*Note:* Abbreviations: HC = healthy controls; OCD = obsessive-compulsive disorder; SAD = social anxiety disorder; CI = confidence interval; SD = standard deviation; AIC = Akaike information criterion; BIC = Bayesian information criterion; LL = log-likelihood. Significant effects are highlighted in bold.

# Table S19: Supplementary BDI-II analyses (model-based P3 analysis)

| *Inferential Statistics of Model-Based P3 Linear Mixed-Effects Model Analyses* | | | | | | | | | |
| --- | --- | --- | --- | --- | --- | --- | --- | --- | --- |
| *Model Formula* | | | P3 ~ BDI-II * agency * feedback valence * SPE +  (1 + agency * feedback valence * SPE \| participant) +  (1 \| electrode) | | | | | | |
|  |  |  |  | | | | | | |
| *Sampling Units* | | |  | | | | | | |
|  | | | *N* observations = 147052 | | | | | | |
|  | | | *N* participants = 81 (27 OCD, 27 HC, 27 SAD) | | | | | | |
|  | | | *N* electrodes = 4 (Cz, CP1, CP2, Pz) | | | | | | |
|  | | |  | | | | | | |
| *Fixed Effects* | | |  | | | | | | |
|  | | | *b* | 95% CI | | *t* | *df* | | *p* |
| **Intercept** | | | **11.96** | **10.82 – 13.10** | | **20.87** | **81.57** | | **<.001** |
| BDI-II | | | 0.08 | -0.03 – 0.18 | | 1.44 | 79.45 | | .153 |
| **Agency** | | | **-7.43** | **-8.66 – -6.20** | | **-12.03** | **78.42** | | **<.001** |
| Feedback Valence | | | -0.47 | -0.99 – 0.05 | | -1.79 | 75.63 | | .078 |
| **SPE** | | | **1.56** | **0.92 – 2.20** | | **4.88** | **54.89** | | **<.001** |
| BDI-II × Agency | | | 0.05 | -0.07 – 0.17 | | 0.85 | 78.57 | | .398 |
| BDI-II ×Feedback Valence | | | -0.02 | -0.07 – 0.03 | | -0.68 | 75.06 | | .502 |
| Agency × Feedback Valence | | | 0.36 | -0.62 – 1.34 | | 0.74 | 72.39 | | .464 |
| BDI-II × SPE | | | 0.04 | -0.02 – 0.11 | | 1.40 | 57.99 | | .166 |
| Agency × SPE | | | -1.27 | -2.58 – 0.04 | | -1.93 | 62.44 | | .058 |
| **Feedback Valence × SPE** | | | **-7.74** | **-10.98 – -4.51** | | **-4.79** | **59.57** | | **<.001** |
| BDI-II × Agency × Feedback Valence | | | 0.02 | -0.08 – 0.11 | | 0.37 | 71.67 | | .716 |
| BDI-II × Agency × SPE | | | 0.02 | -0.11 – 0.15 | | 0.28 | 66.4 | | .783 |
| BDI-II × Feedback Valence × SPE | | | 0.18 | -0.14 – 0.50 | | 1.13 | 61.83 | | .264 |
| **Agency × Feedback Valence × SPE** | | | **9.75** | **3.84 – 15.66** | | **3.31** | **57.14** | | **.002** |
| BDI-II × Agency × Feedback Valence × SPE | | | -0.18 | -0.76 – 0.40 | | -0.62 | 59.45 | | .540 |
|  |  |  | |  | |  |  | |  |
| *Random Effects* | | |  |  | |  |  | |  |
|  | | | Variance | *SD* | Correlation | | | | |
| Participant (Intercept) | | | 24.99 | 5.00 |  | | | | |
| Participant (Agency) | | | 29.17 | 5.40 | -0.38 | | | | |
| Participant (Feedback Valence) | | | 4.63 | 2.15 | 0.19 0.08 | | | | |
| Participant (SPE) | | | 5.94 | 2.44 | 0.18 -0.21 -0.10 | | | | |
| Participant (Agency × Feedback Valence) | | | 16.07 | 4.01 | 0.11 -0.26 -0.63 0.17 | | | | |
| Participant (Agency × SPE) | | | 25.50 | 5.05 | -0.15 0.34 0.39 -0.55 -0.61 | | | | |
| Participant (Feedback Valence × SPE) | | | 190.35 | 13.80 | -0.13 0.15 0.13 -0.39 0.04 0.27 | | | | |
| Participant (Agency × Feedback Valence × SPE) | | | 619.99 | 24.90 | -0.05 -0.23 -0.17 0.53 0.23 -0.36 -0.48 | | | | |
| Electrode (Intercept) | | | 0.06 | 0.24 |  | |  | |  |
| Residual | | | 124.22 | 11.15 |  | |  | |  |
|  | | |  |  | |  |  | |  |
| *Goodness of fit* | | |  |  | |  |  | |  |
|  | | | AIC | BIC | | LL | | Deviance | |
|  | | | 1128361 | 1128896 | | -564126.7 | | 1128253 | |
|  | | | Marginal R^2^ | | | Conditional R^2^ | | | |
|  | | | 0.072 | | | 0.314 | | | |

*Note:* Abbreviations: HC = healthy controls; OCD = obsessive-compulsive disorder; SAD = social anxiety disorder; CI = confidence interval; SD = standard deviation; AIC = Akaike information criterion; BIC = Bayesian information criterion; LL = log-likelihood. Significant effects are highlighted in bold.

# Figure S1: Task performance across blocks


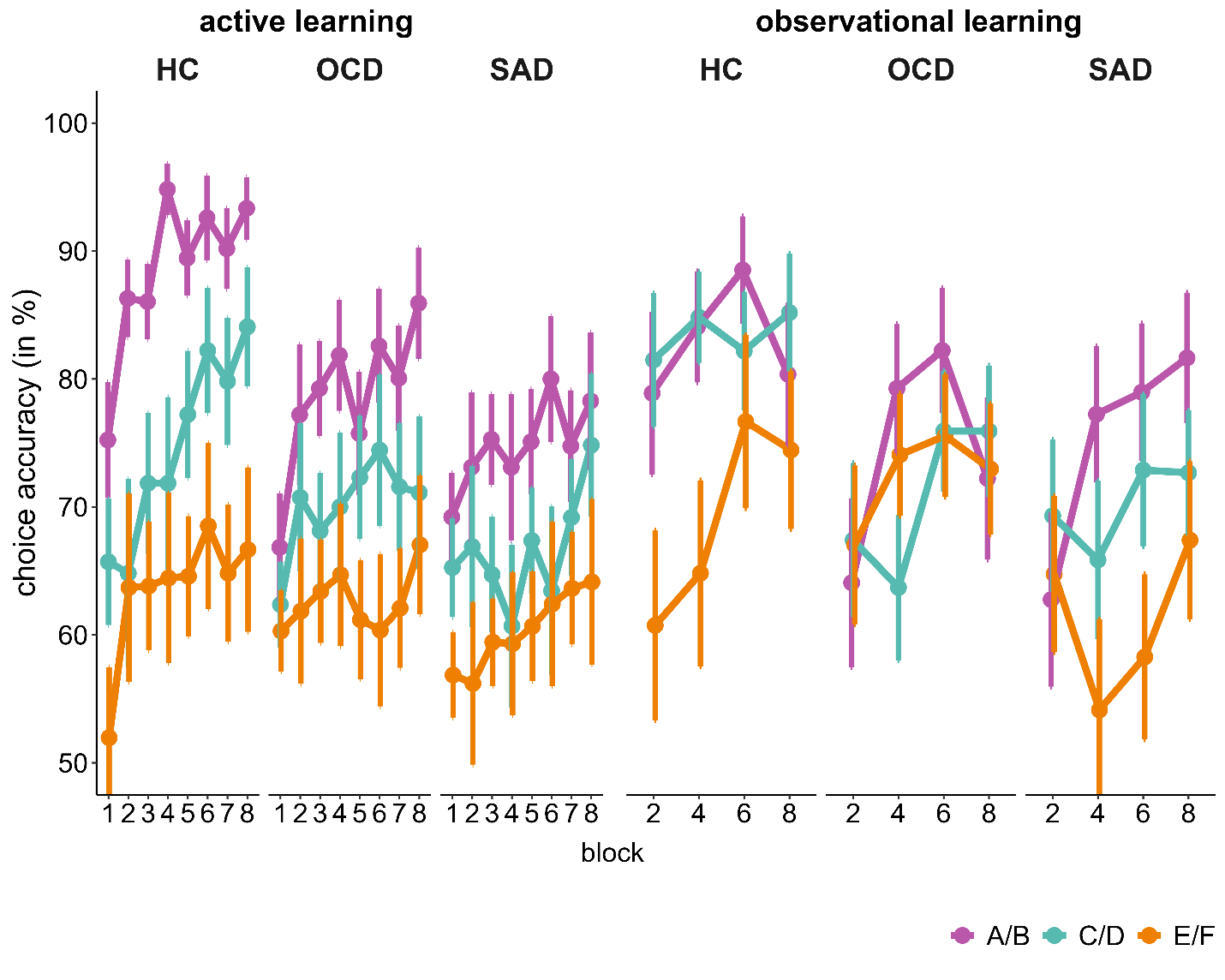


*Note*: Mean choice accuracy per experimental block in active and observational learning for each group and stimulus pair. Error bars represent the standard error of the mean. Abbreviations: HC = healthy controls; OCD = obsessive-compulsive disorder; SAD = social anxiety disorder.

# Figure S2a: Active reinforcement learning model


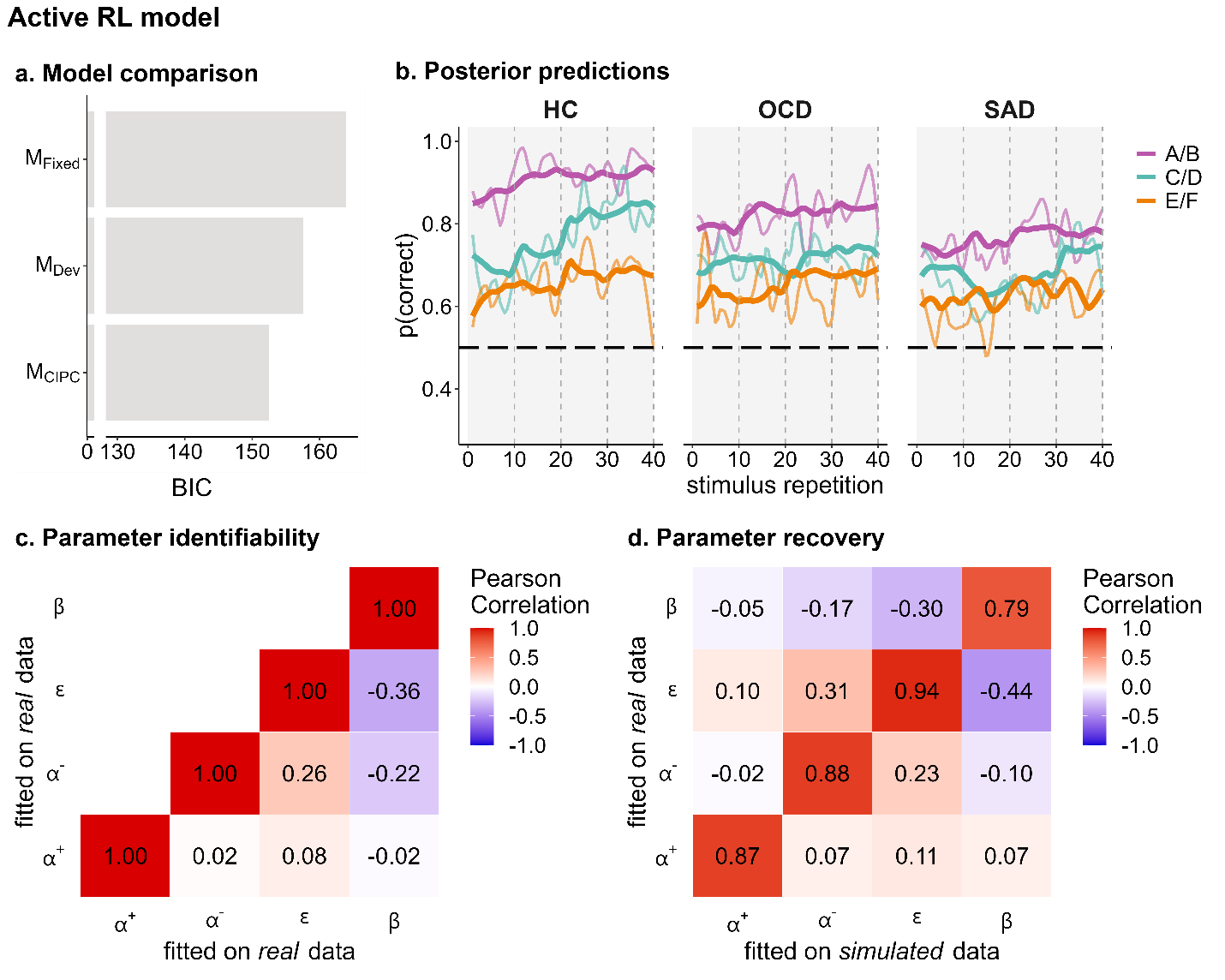


*Note*: Active RL model results. **a.** displays results from model comparison showing that the winning active RL model included choice-induced preference change parameter. **b.** presents posterior predictions for the winning model, with smoothed empirical data (using locally estimated scatterplot smoothing) shown in lighter color intensity. **c.** and **d.** show model validation results for the winning active model showing overall good parameter identifiability and parameter recovery. Abbreviations: RL = reinforcement learning; HC = healthy controls; OCD = obsessive-compulsive disorder; SAD = social anxiety disorder.

# Figure S2b: Active reinforcement learning model


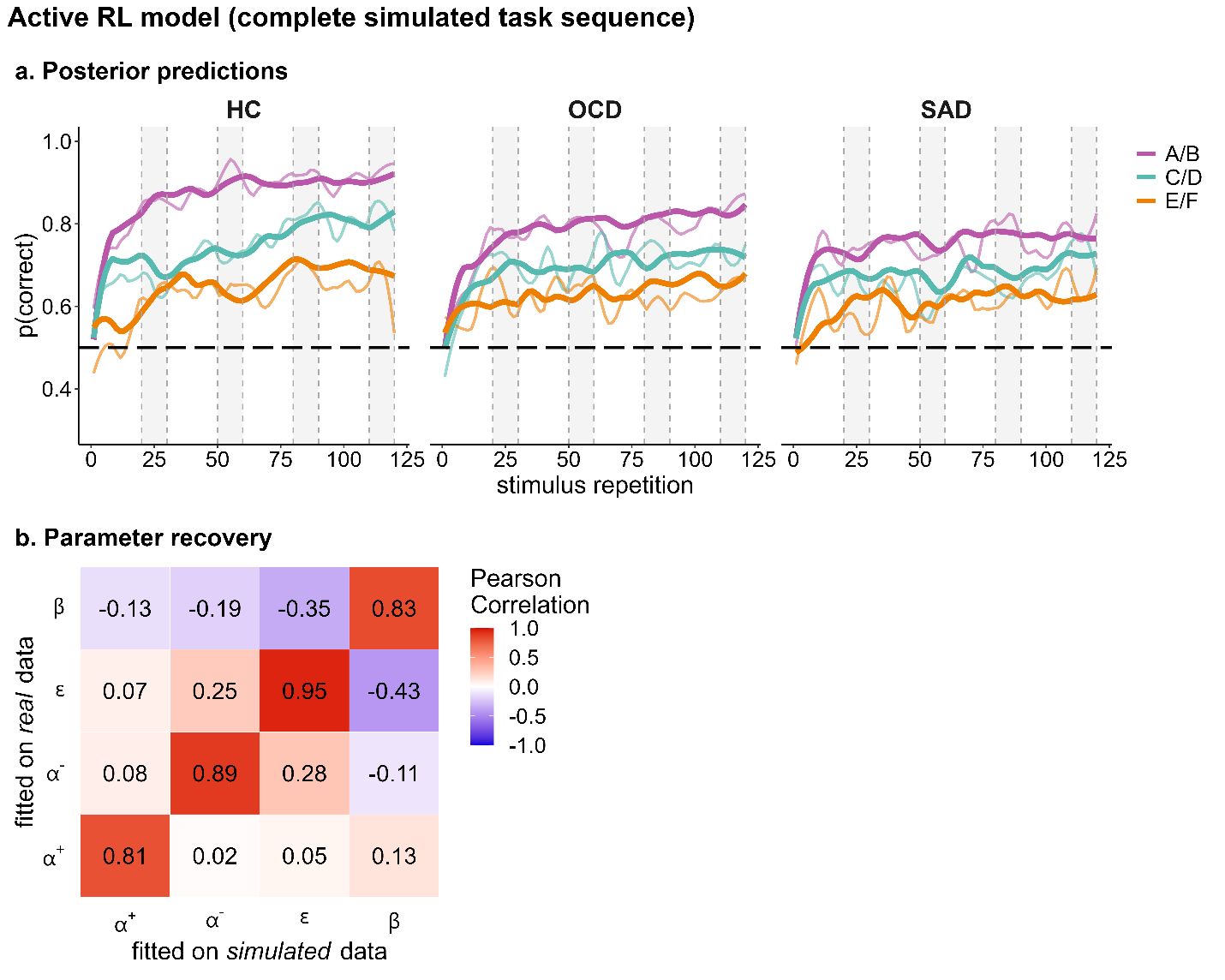


*Note*: Active RL model results when simulating the complete task sequence. **a.** presents posterior predictions for the winning model, with smoothed empirical data (using locally estimated scatterplot smoothing) shown in lighter color intensity. Gray-shaded areas highlight test trials where no feedback was provided. **b.** shows model validation results with overall good parameter recovery when simulating the whole task sequence. Abbreviations: RL = reinforcement learning; HC = healthy controls; OCD = obsessive-compulsive disorder; SAD = social anxiety disorder.

# Figure S3: Observational reinforcement learning model


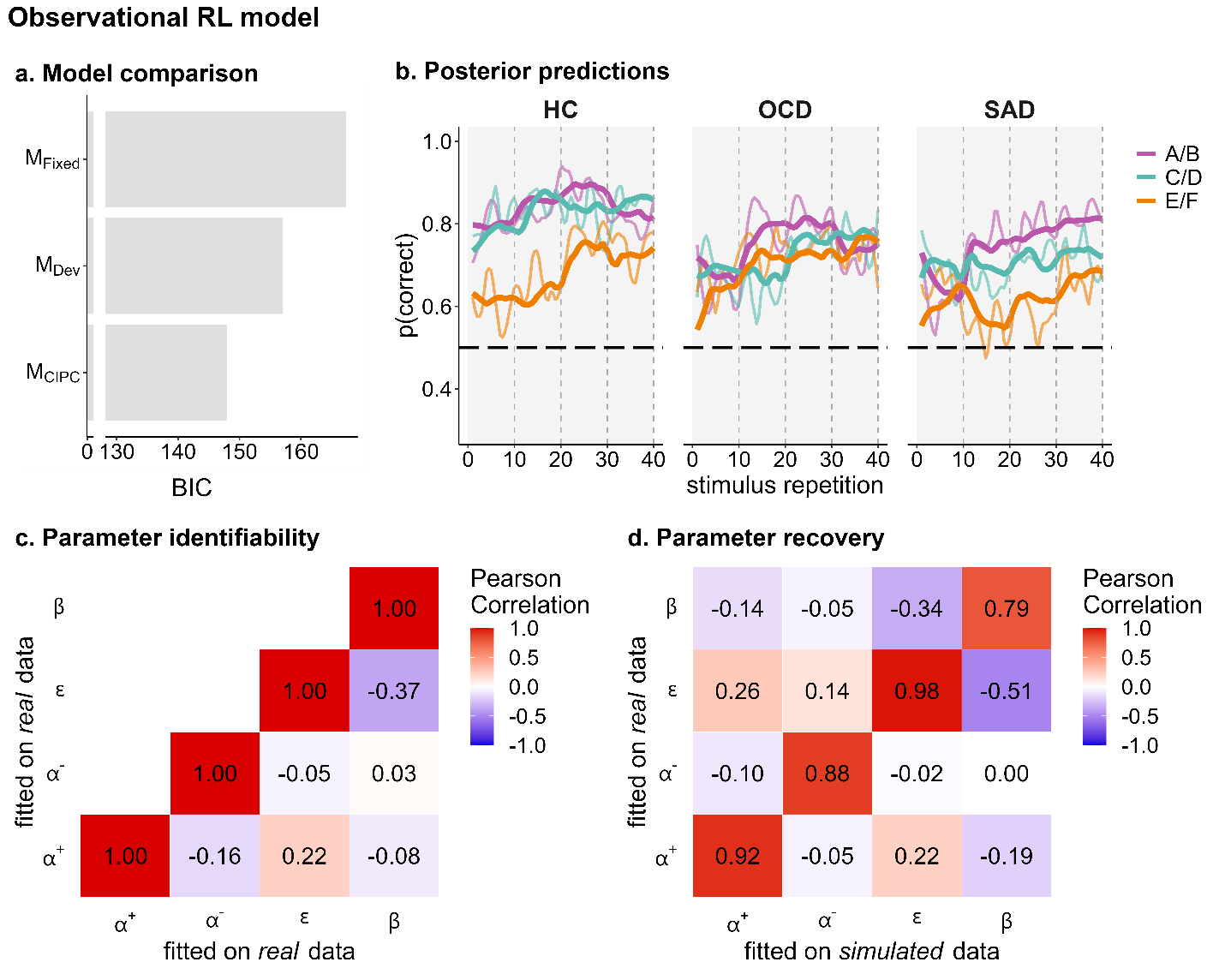


*Note*: Observational RL model results. **a.** displays results from model comparison showing that the winning observational RL model included a choice-induced preference change parameter. **b.** presents posterior predictions for the winning model, with smoothed empirical data (using locally estimated scatterplot smoothing) shown in lighter color intensity. **c.** and **d.** show model validation results for the winning observational model showing overall good parameter identifiability and parameter recovery. Abbreviations: RL = reinforcement learning; HC = healthy controls; OCD = obsessive-compulsive disorder; SAD = social anxiety disorder.
